# Natural Products for Pesticides Discovery: Structural Diversity Derivation and Biological Activities of Naphthoquinones Plumbagin and Juglone

**DOI:** 10.3390/molecules28083328

**Published:** 2023-04-09

**Authors:** Kaihua Wang, Beibei Wang, Henan Ma, Ziwen Wang, Yuxiu Liu, Qingmin Wang

**Affiliations:** 1 State Key Laboratory of Elemento-Organic Chemistry, Research Institute of Elemento-Organic Chemistry, College of Chemistry, Collaborative Innovation Center of Chemical Science and Engineering (Tianjin), Nankai University, Tianjin 300071, China; 2 Tianjin Key Laboratory of Structure and Performance for Functional Molecules, College of Chemistry, Tianjin Normal University, Tianjin 300387, China

**Keywords:** naphthoquinone analogues, plumbagin, juglone, fungicidal activity, lead discovery, structural diversity derivation, antiviral activity, insecticidal activity

## Abstract

Plant diseases and insect pests seriously affect the yield and quality of crops and are difficult to control. Natural products are an important source for the discovery of new pesticides. In this work, naphthoquinones plumbagin and juglone were selected as parent structures, and a series of their derivatives were designed, synthesized and evaluated for their fungicidal activities, antiviral activities and insecticidal activities. We found that the naphthoquinones have broad-spectrum anti-fungal activities against 14 types of fungus for the first time. Some of the naphthoquinones showed higher fungicidal activities than pyrimethanil. Compounds **I**, **I-1e** and **II-1a** emerged as new anti-fungal lead compounds with excellent fungicidal activities (EC_50_ values: 11.35–17.70 µg/mL) against *Cercospora*, *arachidicola Hori*. Some compounds also displayed good to excellent antiviral activities against the tobacco mosaic virus (TMV). Compounds **I-1f** and **II-1f** showed similar level of anti-TMV activities with ribavirin, and could be used as new antiviral candidates. These compound also exhibited good to excellent insecticidal activities. Compounds **II-1d** and **III-1c** displayed a similar level of insecticidal activities with matrine, hexaflumuron and rotenone against *Plutella xylostella*. In current study, plumbagin and juglone were discovered as parent structures, which lays a foundation for their application in plant protection.

## 1. Introduction

With the continuous growth of the population, the improvement of crop yields and quality has gradually become a very challenging issue [1]. It is estimated that the annual economic losses caused by plant diseases exceed $220 billion worldwide, that almost all crops are attacked by diseases and pests, and about one third of agricultural products depend on the use of agricultural chemicals [2,3,4]. In addition, traditional pesticides are prone to produce drug resistance in the long-term use process. Therefore, the continuous innovation of modern agrochemicals has always been an important strategy to deal with this challenge [5,6].

Botanical natural products refer to derivatives of plants, crude plant extracts or active ingredients. Due to their advantages such as biodegradability, diverse structures, extensive sources and low susceptibility to drug resistance, developing botanical natural products into pesticides is recommended as an eco-chemical and sustainable strategy for agricultural pest management. At the same time, using natural products as active leads and optimizing their molecular structure through structural diversity synthesis has also gradually become a direction in the creation of green and efficient new pesticides [7,8,9,10,11,12,13].

**Figure 1:**
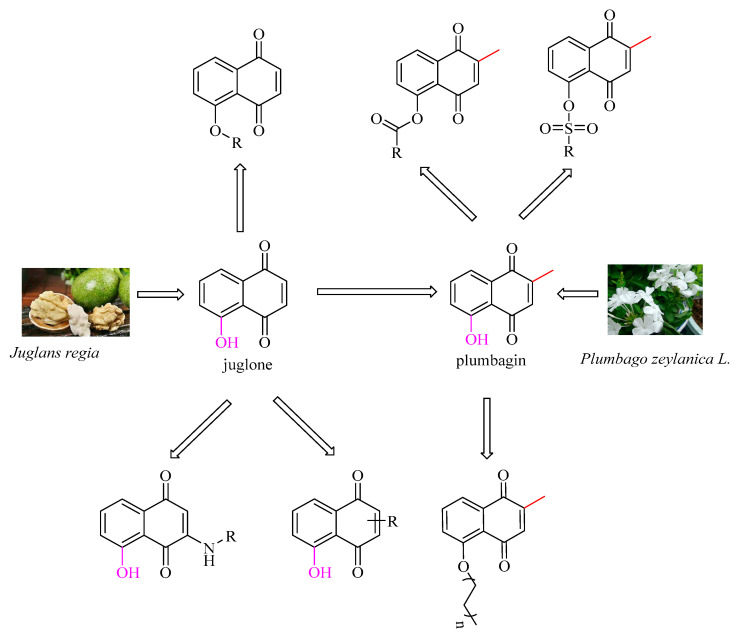
Molecular design and structural optimization based on plumbagin (**I**) and juglone (**II**).

1,4-Naphthoquinones represent a wide group of natural products and are found in several plant families (*Juglans regia*, *Arnebia euchroma*, *P. zeylanica*, etc.), fungi and bacteria. Juglone and plumbagin (Figure 1, existing in herbs such as *Juglans regia* and *Plumbago zeylanica* L.) are a special class of 1,4-naphthoquinone natural products. The phytochemical and pharmacological properties of juglone and plumbagin have been systematically reviewed recently [14,15]. Due to their advantages such as simple structure, good water solubility, simple synthesis process and low cost, they have become a research topic in drug- and pesticide-developing field [16].

Our research group has been committed to the discovery of new and efficient agrochemical lead compounds based on natural products for a long time. Through our research, a series of alkaloids, sesquiterpenes, amino acids and quaternary ammonium salts were found to have good to excellent fungicidal activities, antiviral activities or insecticidal activities, by which we accumulated rich experience in natural product selection and structural optimization [17,18,19,20,21].

In this work, plumbagin and juglone were selected as parent structures, and a series of their derivatives were designed (Figure 1), synthesized and evaluated for their fungicidal activities, antiviral activities and insecticidal activities.

## 2. Results and Discussion

### 2.1. Chemistry

**Scheme 1:**
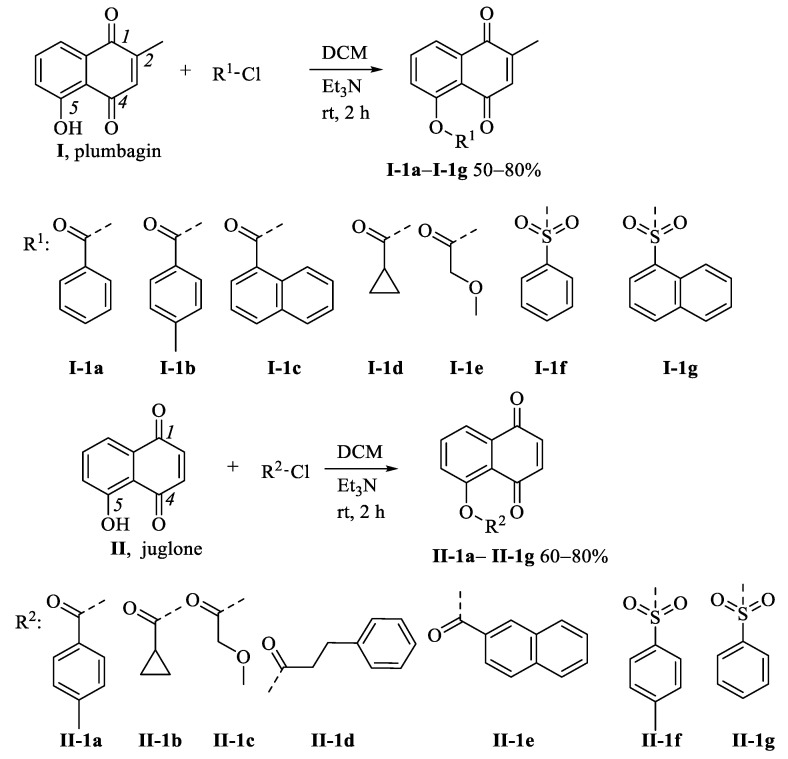
Synthesis of compounds **I-1a**–**I-1g** and **II-1a**–**II-1g**.

**Scheme 2:**
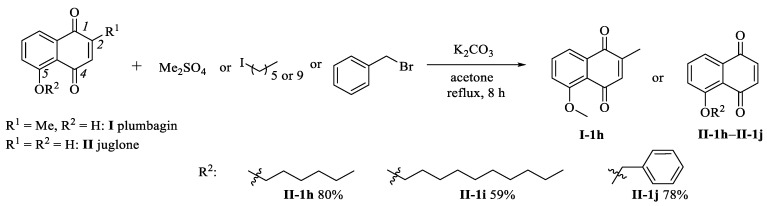
Synthesis of **I-1h** and **II-1h**–**II-1j**.

**Scheme 3:**
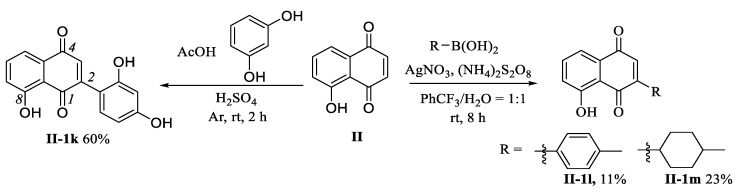
Synthesis of **II-1k**–**II-1m**.

**Scheme 4:**
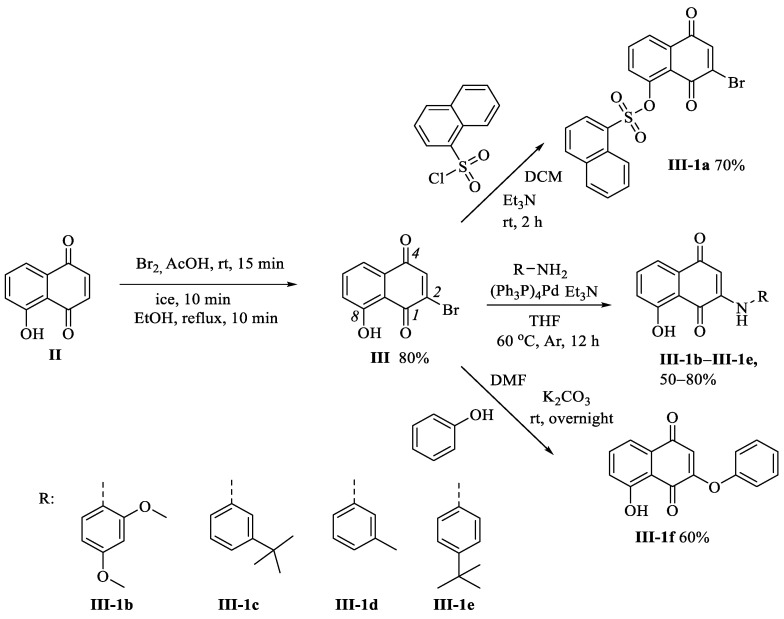
Synthesis of **III-1a**–**III-1f**.

Based on the scaffold hopping strategy [22], a series of structure modified derivatives of juglone and plumbagin were designed and synthesized. The selection of substituents was mainly based on aromatic rings, supplemented by long-chain alkyl, cycloalkyl and oxyalkyl. In the *O*-acylation reaction of juglone and plumbagin, different acyl chlorides and sulfonyl chlorides were used, with triethylamine as a base to give the corresponding esters **I-1a**–**I-1g** and **II-1a**–**II-1g** in good to high yields (Scheme 1). In order to study the effect of chain-like alkyl substituents at the 5-OH on the activities of juglone and plumbagin derivatives, these *O*-alkalation products (**I-1h**, **II-1h**–**II-1j**) were obtained by reacting juglone and plumbagin with corresponding haloalkanes or Me_2_SO_4_ (Scheme 2). In order to study the effect of different substituents at the 3-position of juglone on the activity, compounds **II-1k**–**II-1m**, **III** and **III-1b**–**III-1f** with different substituents were designed and synthesized (Scheme 3 and Scheme 4).

### 2.2. Fungicidal Activity Result and Structure-Activity Relationship (SAR)

The aforementioned process is an effective approach to find new type fungicidal lead compounds based on natural products [23]. We first investigated the fungicidal activities of naphthoquinones **I**–**III**, **I-1a**–**I-1h**, **II-1a**–**II-1m** and **III-1a**–**III-1f** with commercial fungicides carbendazim, chlorothalonil and pyrimethanil as controls. These naphthoquinones exhibited broad-spectrum fungicidal activities against 14 types of phytopathogenic fungi at 50 μg/mL (Table 1). Some derivatives displayed more than 60% inhibitory effects. Compounds **I** and **I-1e** both had excellent inhibitory rates against *Fusarium oxysporum* f. sp. *cucumeris*. **I**, **I-1e** and **II-1a** showed a better inhibition rate than three commercial fungicides against *Cercospora arachidicola Hori*. **I-1e** also showed a comparable activity to commercial fungicides against *Rhizoctonia cerealis* and *Fusarium moniliforme*. Plumbagin (**I**) was effective against *Cercospora arachidicola Hori*, *Phytophthora capsici* and *Rhizoctonia solani* with a 100% inhibitory rate.

Compounds **I**, **I-1e** and **II-1a** with excellent antifungal activities against *Cercospora arachidicola Hori* were further tested to determine their EC_50_ values, with pyrimethanil as a control (Table 2). It can be seen that the EC_50_ values of **I**, **I-1e** and **II-1a** (11.35–17.70 µg/mL) are lower than that of pyrimethanil (EC_50_ value: 19.17 µg/mL), which indicates that **I**, **I-1e** and **II-1a** have better fungicidal activities than pyrimethanil and can be used as novel antifungal candidates for further investigation.

SAR against *Cercospora arachidicola Hori*: Natural product plumbagin (**I**) displayed excellent fungicidal activity. Jonlone (**II**) or the introduction of bromine at the 3-position of **II** (i.e., **III**) leads to extremely reduced activity (inhibition effect: **I** > **II**, **III**). The introduction of an aliphatic functional group onto the 5-hydroxyl of plumbagin (**I**) is more advantageous than the introduction of an aromatic functional group, as compound **I-1e** containing a methoxyacetyl group displayed better antifungal activity than **I** (EC_50_ values: **I** > **I-1e**). On the other hand, among ten 5-hydroxy modified derivatives of juglone (**II**), all showed higher fungicidal activities than **II**, except compound **II-1i**, having a long decyl chain (inhibition effect: **II-1a**–**II-1h**, **II-1j** > **II** > **II-1i**). The activities of the aryl formyl-substituted derivatives **II-1a** and **II-1e** were more prominent, and the 3-position of **II** substituted derivatives **II-1k**–**II-1m** showed higher antifungal activities than **II**, but the activity level was moderate, except for compound **II-1d**. The overall activities of the derivatives containing bromine at the 3-position of **II** (**III** and **III-1a**) are poor, indicating that the introduction of a bromine atom at the 3-position of **II** is very unfavorable to the activity.

### 2.3. Antiviral Activity Result and Structure-Activity Relationship (SAR)

There are many types of plant viruses, and their control is extremely difficult since one virus can infect one or several plants, and one plant can be infected by one or several viruses. There are few practical and efficient antiviral agents for plants. As a commonly used anti-plant virus agent, ribavirin can only give an inhibition effect of less than 50% at 500 µg/mL. Therefore, it is particularly important to find new antiviral lead compounds [24,25].

Tobacco mosaic virus (TMV) is the earliest discovered and most deeply studied plant virus. It can infect a variety of *Solanaceae* plants, and is often used as a model virus for screening new plant antiviral agents [17,18,19].

As shown in Table 3, naphthoquinones **I**–**III**, **I-1a**–**I-1h**, **II-1a**–**II-1m** and **III-1a**–**III-1f** were also found to have good anti-TMV activities for the first time. Compounds **I-1f**, **II-1f**, **II-1g**, **II-1l**, **III-1b** and **III-1d** exhibited a similar or slightly higher level of antiviral activities. The introduction of benzenesulfonyl on the 8-hydroxyl can greatly improve the antiviral activities of these compounds. **I-1f** and **II-1f** emerged as new antiviral candidates with excellent inhibitory effects.

### 2.4. Insecticidal Activity Result and Structure-Activity Relationship (SAR)

In order to further expand the application range of these compounds, we studied the insecticidal activities of **I–III**, **I-1a**–**I-1h**, **II-1a**–**II-1m** and **III-1a**–**III-1f** against seven common crop pests (*Mythimna separata*; *Helicoverpa armigera*; *Spodoptera frugiperda*; *Ostrinia nubilalis*; *Aphis craccivora*; *Tetranychus cinnabarinus*; *Plutella xylostella*) and on sanitary pests (*Culex pipiens*) in China [20], with commercial synthetic insecticide hexaflumuron and two natural insecticides matrine and rotenone as controls.

It can be seen from Table 4 that naphthoquinones **I–III**, **I-1a**–**I-1h**, **II-1a**–**II-1m** and **III-1a**–**III-1f** showed different degrees of insecticidal activities against various pests, especially against *Plutella xylostella*. Compounds **I-1h**, **II-1b**, **II-1e**, **II-1h**, **II-1j** and **III-1c** exhibited good mortality against *Tetranychus cinnabarinus* at a specific concentration (600 μg/mL or 200 μg/mL). The insecticidal activities of **II-1b**, **II-1d**, **II-1h**, **III-1c** and **III-1d** against *Plutella xylostella* were better than those of matrine and rotenone, furthermore, **II-1d** (phenylpropionionic acid ester of **II**) and **III-1c**, with a similar level of insecticidal activities as hexaflumuron, emerged as new insecticide candidates against *Plutella xylostella*.

SAR against *Plutella xylostella*: The modification of the 5-hydroxyl of plumbagin (**I**) is disadvantageous to its activity. However, the introduction of *p*-methylbenzoyl, cyclopropyl, phenylpropionyl, *n*-hexyl or benzyl into the 5-hydroxyl of juglone (**II**) is beneficial to the improvement of its activity. The introduction of an aryl, alkyl, bromine or *o*/*p*-substituted aniline group at the 3-position of juglone (**II**) leads to the reduction of its activity, the introduction of *m*-substituted phenyl amino group (**III-1c**, **III-1d**) can improve the activity, and the introduction of phenoxy group (**III-1f**) basically maintains the activity.

## 3. Materials and Methods

### 3.1. Synthetic Procedures

#### 3.1.1. Chemicals

The reagents (including ultra-dry dichloromethane) were purchased from commercial sources and were used as received. All anhydrous solvents were dried and purified by standard techniques prior to use. Naphthoquinones plumbagin (**I**) and juglone (**II**) were prepared using reported method [16]. 

#### 3.1.2. Instruments

The melting points of the compounds were tested on an X-4 melting point apparatus (Beijing Tech Instruments Company, Beijing, China) without correction. NMR spectra were obtained through a 400 MHz (100 MHz for ^13^C) instrument (Bruker, Billerica, MA, USA) at room temperature with either CDCl_3_ or DMSO-*d*_6_ as the solvent. Chemical shifts were referenced to tetramethylsilane as an internal standard, or to solvent peaks of DMSO-*d*_6_ (^1^H: *δ* = 2.50 ppm; ^13^C: *δ* = 39.52 ppm) or CDCl_3_ (^1^H: *δ* = 7.26 ppm; ^13^C: *δ* = 77.16 ppm). The following abbreviations are used to designate chemical shift multiplicities: s = singlet, d = doublet, dd = doublet of doublets, t = triplet, m = multiplet and brs = broad singlet. High-resolution mass spectra were obtained with an Ionspec, 7.0 T Fourier transform ion cyclotron resonance mass spectrometer (Bruker, Saarbrucken, Germany).

#### 3.1.3. Preparation of 2-Bromo-8-hydroxynaphthalene-1,4-dione (**III**)

A mixture of juglone (4.00 g, 23 mmol), glacial AcOH (60 mL), Br_2_ (1.2 mL, 23.4 mmol) in a 250 mL single-neck flask was stirred at room temperature for 15 min, then quenched with crushed ice and filtered. The solid was immediately transferred to a 100 mL single-neck bottle containing preheated ethanol. Then, the reaction mixture was refluxed for 10 min, cooled to room temperature, filtered and air-dried to obtain a reddish-brown solid, yield 80%, mp 172–174 °C. ^1^H NMR (400 MHz, CDCl_3_) *δ* 11.72 (s, 1H), 7.69–7.61 (m, 2H), 7.48 (s, 1H), 7.30 (dd, *J* = 8.0, 1.4 Hz, 1H). ^13^C NMR (100 MHz, CDCl_3_) *δ* 182.9, 181.6, 162.1, 141.2, 139.3, 137.2, 131.7, 124.8, 120.0, 114.0.

#### 3.1.4. Preparation of Compounds **I-1a**–**I-1g**, **II-1a**–**II-1g**, **III-1a**

A 0.10 mol/L solution of triethylamine (0.6 mL, 2 equiv.) in ultra-dry dichloromethane was added to a 50 mL round bottom flask containing substrate **I**, **II** or **III**. Then corresponding acyl chloride (2.6 mmol, 1.2 equiv.) was slowly added under ice bath condition. The mixture was stirred at room temperature for 1 h, quenched by H_2_O (100 mL) and then extracted with dichloromethane (50 mL × 3). The combined organic layer was washed with brine, dried over anhydrous Na_2_SO_4_ and concentrated. The residue was subjected to column chromatography (petroleum ether: ethyl acetate, 10:1, *v*/*v*) to obtain the corresponding product.

**I-1a** (6-Methyl-5,8-dioxo-5,8-dihydronaphthalen-1-yl benzoate): Red brown solid, yield 60%, mp 148–150 °C. ^1^H NMR (400 MHz, CDCl_3_) *δ* 8.25 (d, *J* = 7.5 Hz, 2H), 8.11 (d, *J* = 7.7 Hz, 1H), 7.78 (t, *J* = 7.9 Hz, 1H), 7.66 (t, *J* = 7.4 Hz, 1H), 7.58–7.47 (m, 3H), 6.68 (d, *J* = 1.1 Hz, 1H), 2.16 (d, *J* = 0.8 Hz, 3H). ^13^C NMR (100 MHz, CDCl_3_) *δ* 184.9, 183.5, 165.1, 149.5, 146.9, 136.9, 134.4, 133.9, 133.7, 130.4, 129.7, 129.4, 128.6, 125.2, 123.7, 16.1.

**I-1b** (6-Methyl-5,8-dioxo-5,8-dihydronaphthalen-1-yl 4-methylbenzoate): Yellow oil, yield 67%. ^1^H NMR (400 MHz, CDCl_3_) *δ* 8.11 (d, *J* = 8.2 Hz, 2H), 8.08 (dd, *J* = 7.8, 1.2 Hz, 1H), 7.75 (t, *J* = 7.9 Hz, 1H), 7.47 (dd, *J* = 8.1, 1.2 Hz, 1H), 7.32 (d, *J* = 8.0 Hz, 2H), 6.66 (q, *J* = 1.5 Hz, 1H), 2.45 (s, 3H), 2.14 (d, *J* = 1.5 Hz, 3H). ^13^C NMR (100 MHz, CDCl_3_) *δ* 185.0, 183.5, 165.2, 149.7, 146.8, 144.6, 136.9, 134.4, 133.9, 130.5, 129.7, 129.4, 126.7, 125.1, 124.1, 21.8, 16.0.

**I-1c** (6-Methyl-5,8-dioxo-5,8-dihydronaphthalen-1-yl 2-naphthoate): Yellow oil, yield 50%. ^1^H NMR (400 MHz, CDCl_3_) *δ* 8.85 (s, 1H), 8.23 (dd, *J* = 8.6, 1.7 Hz, 1H), 8.14 (dd, *J* = 7.8, 1.2 Hz, 1H), 8.05–7.90 (m, 2H), 7.81 (t, *J* = 7.9 Hz, 1H), 7.69–7.52 (m, 2H), 6.69 (q, *J* = 1.5 Hz, 1H), 2.17 (d, *J* = 1.5 Hz, 3H). ^13^C NMR (100 MHz, CDCl_3_) *δ* 184.9, 183.5, 165.3, 149.6, 146.9, 136.9, 136.0, 134.4, 134.0, 132.6, 132.3, 129.7 128.6, 127.9, 126.7, 125.6, 125.2, 123.8, 16.1.

**I-1d** (6-Methyl-5,8-dioxo-5,8-dihydronaphthalen-1-yl cyclopropanecarboxylate): Yellow brown crystals, yield 70%, mp 125−127 °C. ^1^H NMR (400 MHz, CDCl_3_) *δ* 8.07 (d, *J* = 7.7 Hz, 1H), 7.73 (t, *J* = 7.9 Hz, 1H), 7.39 (d, *J* = 8.1 Hz, 1H), 6.73 (s, 1H), 2.18 (s, 3H), 2.05–1.96 (m, 1H), 1.30 (dd, *J* = 7.6, 3.8 Hz, 2H), 1.14 (dd, *J* = 7.8, 3.1 Hz, 2H). ^13^C NMR (100 MHz, CDCl_3_) *δ* 184.9, 183.6, 173.0, 149.4, 146.8, 136.9, 134.3, 133.8, 129.5, 125.0, 123.8, 16.1, 13.3, 9.3. HRMS C_15_H_12_NaO_4_ [M + Na]^+^ cald. 279.0628, found 279.0626.

**I-1e** (6-Methyl-5,8-dioxo-5,8-dihydronaphthalen-1-yl 2-methoxyacetate): Yellow solid, yield 80%, mp 98–100 °C. ^1^H NMR (400 MHz, CDCl_3_) *δ* 8.09 (d, *J* = 7.7 Hz, 1H), 7.75 (t, *J* = 7.9 Hz, 1H), 7.39 (d, *J* = 8.0 Hz, 1H), 6.71 (s, 1H), 4.49 (s, 2H), 3.61 (s, 3H), 2.17 (s, 3H). ^13^C NMR (100 MHz, CDCl_3_) *δ* 184.7, 183.5, 168.9, 148.7, 147.1, 136.8, 134.6, 133.9, 129.3, 125.4, 123.3, 69.9, 59.7, 16.1. HRMS C_14_H_12_NaO_5_ [M + Na]^+^ cald. 283.0577, found 283.0575.

**I-1f** (6-Methyl-5,8-dioxo-5,8-dihydronaphthalen-1-yl benzenesulfonate): Red brown solid, yield 60%, mp 105–107 °C. ^1^H NMR (400 MHz, CDCl_3_) *δ* 7.99–7.93 (m, 2H), 7.69–7.63 (m, 1H), 7.55–7.50 (m, 2H), 7.25–7.20 (m, 2H), 6.77 (dd, *J* = 6.4, 2.0 Hz, 1H), 6.65 (s, 1H), 2.28 (s, 3H). ^13^C NMR (100 MHz, CDCl_3_) *δ* 147.8, 146.3, 137.5, 137.0, 134.2, 131.1, 129.3, 128.6, 128.2, 115.0, 112.7, 111.4, 108.9, 102.1, 25.2. HRMS C_17_H_13_O_5_S [M + H]^+^ cald. 329.0478, found 329.0477.

**I-1g** (6-Methyl-5,8-dioxo-5,8-dihydronaphthalen-1-yl naphthalene-1-sulfonate): Brown solid, yield 60%, mp 140–142 °C. ^1^H NMR (400 MHz, CDCl_3_) *δ* 8.92 (d, *J* = 8.7 Hz, 1H), 8.20 (dd, *J* = 11.8, 7.9 Hz, 2H), 8.01 (d, *J* = 8.1 Hz, 1H), 7.75 (dd, *J* = 15.6, 7.4 Hz, 1H), 7.69–7.65 (m, 1H), 7.56–7.48 (m, 1H), 7.16–7.09 (m, 2H), 6.73 (d, *J* = 7.1 Hz, 1H), 6.63 (s, 1H), 2.18 (s, 3H). ^13^C NMR (100 MHz, CDCl_3_) *δ* 147.8, 146.3, 137.9, 135.5, 134.2, 133.5, 133.2, 130.9, 129.7, 128.2, 128.9, 128.7, 127.4, 125.4, 124.2, 114.8, 112.6, 111.3, 108.8, 102.1, 25.2. HRMS C_21_H_15_O_5_S [M + H]^+^ cald. 379.0635, found 379.0633.

**II-1a** (5,8-Dioxo-5,8-dihydronaphthalen-1-yl 4-methylbenzoate): Light yellow crystals, yield 70%, mp 100–102 °C. ^1^H NMR (400 MHz, CDCl_3_) *δ* 8.14 (d, *J* = 8.2 Hz, 2H), 8.09 (dd, *J* = 7.8, 1.2 Hz, 1H), 7.83–7.79 (m, 1H), 7.53 (dd, *J* = 8.1, 1.2 Hz, 1H), 7.35 (d, *J* = 8.0 Hz, 2H), 6.94 (d, *J* = 10.3 Hz, 1H), 6.82 (d, *J* = 10.3 Hz, 1H), 2.47 (s, 3H). ^13^C NMR (100 MHz, CDCl_3_) *δ* 184.3, 183.6, 165.1, 149.9, 144.7, 140.0, 137.3, 134.8, 133.6, 130.5, 130.3, 130.1, 129.4, 129.2, 125.0, 21.8. HRMS C_18_H_13_O_4_ [M + H]^+^ cald. 293.0808, found 293.0804.

**II-1b** (5,8-Dioxo-5,8-dihydronaphthalen-1-yl cyclopropanecarboxylate): Red brown solid, yield 60%, mp 94–96 °C. ^1^H NMR (400 MHz, CDCl_3_) *δ* 8.04 (dd, *J* = 7.8, 1.2 Hz, 1H), 7.76 (t, *J* = 7.9 Hz, 1H), 7.41 (dd, *J* = 8.1, 1.2 Hz, 1H), 6.94 (d, *J* = 10.3 Hz, 1H), 6.86 (d, *J* = 10.3 Hz, 1H), 1.99 (tt, *J* = 8.1, 4.6 Hz, 1H), 1.30–1.12 (m, 4H). ^13^C NMR (100 MHz, CDCl_3_) *δ* 184.3, 183.7, 173.0, 149.6, 139.94, 137.3, 134.7, 133.5, 129.9, 124.9, 123.6, 13.3, 9.3. HRMS C_14_H_10_NaO_4_ [M + Na]^+^ cald. 265.0471, found 265.0471.

**II-1c** (5,8-Dioxo-5,8-dihydronaphthalen-1-yl 2-methoxyacetate): Yellow brown solid, yield 80%, mp 85–87 °C. ^1^H NMR (400 MHz, CDCl_3_) *δ* 8.08 (d, *J* = 7.7 Hz, 1H), 7.79 (t, *J* = 7.9 Hz, 1H), 7.43 (d, *J* = 8.1 Hz, 1H), 6.95 (d, *J* = 10.3 Hz, 1H), 6.86 (d, *J* = 10.3 Hz, 1H), 4.49 (s, 2H), 3.61 (s, 3H). ^13^C NMR (100 MHz, CDCl_3_) *δ* 184.1, 183.6, 168.9, 148.9, 139.8, 137.5, 135.0, 133.6, 129.7, 125.3, 123.1, 69.9, 59.7. HRMS C_13_H_10_NaO_5_ [M + Na]^+^ cald. 269.0420, found 269.0419.

**II-1d** (5,8-Dioxo-5,8-dihydronaphthalen-1-yl 3-phenylpropanoate): Red brown solid, yield 70%, mp 57–59 °C. ^1^H NMR (400 MHz, CDCl_3_) *δ* 8.03 (d, *J* = 7.7 Hz, 1H), 7.73 (dd, *J* = 8.3 Hz, *J* = 7.7 Hz, 1H), 7.20–7.40 (m, 6H), 6.92 (d, *J* = 10.3 Hz, 1H), 6.83 (d, *J* = 10.3 Hz, 1H), 3.15 (t, *J* = 7.4 Hz, 2H), 3.07 (t, *J* = 7.4 Hz, 2H). ^13^C NMR (100 MHz, CDCl_3_) *δ* 184.2, 183.7, 171.3, 149.5, 140.3, 139.9, 137.3, 134.8, 133.5, 129.8, 128.6, 128.5, 126.4, 125.0, 123.3, 35.8, 30.6. HRMS C_19_H_14_NaO_4_ [M + Na]^+^ cald. 329.0784, found 329.0783.

**II-1e** (5,8-Dioxo-5,8-dihydronaphthalen-1-yl 2-naphthoate): Yellow crystals, yield 70%, mp 102–104 °C. ^1^H NMR (400 MHz, CDCl_3_) *δ* 8.85 (s, 1H), 8.23 (d, *J* = 8.4 Hz, 1H), 8.11 (d, *J* = 7.6 Hz, 1H), 8.05–7.90 (m, 3H), 7.84 (t, *J* = 7.8 Hz, 1H), 7.68–7.55 (m, 3H), 6.95 (d, *J* = 10.3 Hz, 1H), 6.83 (d, *J* = 10.2 Hz, 1H). ^13^C NMR (100 MHz, CDCl_3_) *δ* 184.3, 183.6, 165.3, 149.9, 140.0, 137.4, 136.0, 134.9, 133.6, 132.6, 132.4, 131.2, 130.1, 129.6, 128.8, 128.5, 127.9, 126.9, 125.6, 125.1. HRMS C_21_H_12_NaO_4_ [M + H] ^+^ cald. 351.0628, found 351.0626.

**II-1f** (5,8-Dioxo-5,8-dihydronaphthalen-1-yl 4-methylbenzenesulfonate): Yellow crystals, yield 80%, mp 149–151 °C. ^1^H NMR (400 MHz, CDCl_3_) *δ* 8.12–8.05 (m, 1H), 7.89 (dd, *J* = 8.0, 4.7 Hz, 2H), 7.74 (td, *J* = 7.9, 4.7 Hz, 1H), 7.57 (dd, *J* = 7.1, 4.5 Hz, 1H), 7.36 (d, *J* = 7.8 Hz, 2H), 6.92 (dd, *J* = 10.3, 4.6 Hz, 1H), 6.83 (dd, *J* = 10.3, 4.6 Hz, 1H), 2.47 (d, *J* = 4.5 Hz, 3H). ^13^C NMR (100 MHz, CDCl_3_) *δ* 183.8, 182.4, 147.0, 145.8, 140.1, 136.9, 134.5, 133.9, 132.6, 130.0, 129.8, 128.8, 125.8, 124.5, 21.8.

**II-1g** (5,8-Dioxo-5,8-dihydronaphthalen-1-yl benzenesulfonate): Yellow solid, yield 70%, mp 145–149 °C. ^1^H NMR (400 MHz, CDCl_3_) *δ* 8.10 (d, *J* = 7.7 Hz, 1H), 8.01 (d, *J* = 7.5 Hz, 2H), 7.74 (dt, *J* = 11.4, 7.8 Hz, 2H), 7.58 (dd, *J* = 17.0, 8.2 Hz, 3H), 6.93 (d, *J* = 10.3 Hz, 1H), 6.82 (d, *J* = 10.3 Hz, 1H). ^13^C NMR (100 MHz, CDCl_3_) *δ* 183.8, 182.5, 146.8, 140.0, 137.0, 135.5, 134.6, 133.8, 131.5, 130.0, 129.2, 128.8, 126.0, 124.5. HRMS C_16_H_10_NaO_5_S [M + Na] ^+^ cald. 337.0141, found 337.0141.

**III-1a** (7-Bromo-5,8-dioxo-5,8-dihydronaphthalen-1-yl naphthalene-1-sulfonate): Yellow brown solid, yield 70%, mp 74–76 °C. ^1^H NMR (400 MHz, CDCl_3_) *δ* 8.82 (d, *J* = 8.2 Hz, 1H), 8.19 (dd, *J* = 7.7, 4.5 Hz, 2H), 8.02 (dd, *J* = 13.3, 5.4 Hz, 2H), 7.78–7.72 (m, 1H), 7.68 (dd, *J* = 12.7, 5.5 Hz, 1H), 7.62–7.54 (m, 2H), 7.45 (s, 1H), 7.09 (dd, *J* = 8.2, 1.0 Hz, 1H). ^13^C NMR (100 MHz, CDCl_3_) *δ* 181.2, 174.9, 148.0, 141.6, 138.8, 136.2, 134.9, 134.3, 134.1, 133.7, 131.6, 130.9, 129.3, 129.1, 129.0, 128.5, 127.5, 126.1, 125.2, 124.1. HRMS C_20_H_11_BrNaO_5_S [M + Na]^+^ cald. 464.9403, found 464.9400.

#### 3.1.5. Preparation of Compounds **I-1h** and **II-1h**–**II-1j**

To a solution of **I** or **II** (0.6 mmol) in acetone was added the corresponding iodide, Me_2_SO_4_ or benzyl bromide (2 equiv.) and potassium carbonate (2 equiv.). The mixture was stirred at room temperature overnight and then concentrated. The residue was taken into water (100 mL) and extracted with ethyl acetate (30 mL × 3). The combined organic layer was washed with brine, dried over anhydrous Na_2_SO_4_ and concentrated. The residue was subjected to column chromatography (petroleum ether: ethyl acetate, 10:1, *v*/*v*) to obtain the corresponding product.

**I-1h** (5-Methoxy-2-methylnaphthalene-1,4-dione): Pale yellow solid, yield 56%, mp 189–191 °C. ^1^H NMR (400 MHz, CDCl_3_) *δ* 7.78 (d, *J* = 7.6 Hz, 1H), 7.68 (t, *J* = 8.0 Hz, 1H), 7.31 (d, *J* = 8.4 Hz, 1H), 6.76 (q, *J* = 1.2 Hz, 1H), 4.02 (s, 3H), 2.16 (d, *J* = 1.2 Hz, 3H). ^13^C NMR (100 MHz, CDCl_3_) *δ* 185.8, 184.58, 159.48, 145.4, 137.9, 134.69, 134.49, 120.09, 119.4, 117.7, 56.5, 15.8.

**II-1h** (5-(Hexyloxy)naphthalene-1,4-dione): Pale yellow solid, yield 80%, mp 52–54 °C. ^1^H NMR (400 MHz, CDCl_3_) *δ* 8.91 (d, *J* = 10.1 Hz, 1H), 7.93 (dd, *J* = 7.6, 1.2 Hz, 1H), 7.58 (t, *J* = 8.0 Hz, 1H), 7.51 (d, *J* = 8.2 Hz, 1H), 6.53 (d, *J* = 10.1 Hz, 1H), 4.37 (t, *J* = 6.7 Hz, 2H), 1.84–1.75 (m, 2H), 1.49–1.32 (m, 6H), 0.91 (t, *J* = 7.0 Hz, 3H). ^13^C NMR (100 MHz, CDCl_3_) *δ* 165.7, 159.7, 154.7, 141.3, 130.8, 128.1, 127.1, 121.2, 118.6, 118.0, 65.9, 53.4, 31.4, 28.6, 25.7, 14.0. HRMS C_16_H_19_O_3_ [M + H]^+^ cald. 259.1329, found 259.1327.

**II-1i** (5-(Decyloxy)naphthalene-1,4-dione): Yellow green solid, yield 59%, mp 50–52 °C. ^1^H NMR (400 MHz, CDCl_3_) *δ* 8.93 (d, *J* = 10.1 Hz, 1H), 7.96 (dd, *J* = 7.6, 1.2 Hz, 1H), 7.60 (t, *J* = 8.0 Hz, 1H), 7.53 (d, *J* = 7.7 Hz, 1H), 6.55 (d, *J* = 10.1 Hz, 1H), 4.39 (t, *J* = 6.7 Hz, 2H), 1.87–1.76 (m, 2H), 1.51–1.43 (m, 2H), 1.40–1.26 (m, 12H), 0.90 (t, *J* = 6.8 Hz, 3H). ^13^C NMR (100 MHz, CDCl_3_) *δ* 165.7, 159.8, 154.7, 141.3, 130.8, 128.1, 127.1, 121.2, 118.6, 118.0, 66.0, 31.9, 29.5, 29.3, 29.3, 28.6, 26.1, 22.7, 14.1. HRMS C_20_H_27_O_3_ [M + H]^+^ cald. 315.1955, found 315.1956.

**II-1j** (5-(Benzyloxy)naphthalene-1,4-dione): Red brown solid, yield 78%, mp 108–110 °C. ^1^H NMR (400 MHz, CDCl_3_) *δ* 8.91 (d, *J* = 10.1 Hz, 1H), 7.97 (dd, *J* = 7.5, 0.9 Hz, 1H), 7.30–7.65 (m, 7H), 6.51 (d, *J* = 10.1 Hz, 1H), 5.40 (s, 2H). ^13^C NMR (100 MHz, CDCl_3_) *δ* 165.4, 159.6, 154.8, 141.1, 135.3, 130.8, 128.8, 128.6, 128.4, 127.6, 127.3, 121.4, 118.7, 118.1, 67.5.

#### 3.1.6. Preparation of 2-(2,4-Dihydroxyphenyl)-8-hydroxynaphthalene-1,4-dione (**II-1k**)

A mixture of juglone (104 mg, 0.6 mmol), resorcinol (66 mg, 0.6 mmol) and H_2_SO_4_ (2 mol/L, 2 mL) in acetic acid (10 mL) was stirred under argon for 2 h at room temperature, quenched with water (50 mL) and then extracted with ethyl acetate (30 mL × 3). The combined organic layer was washed with brine, dried over anhydrous Na_2_SO_4_ and then concentrated. The residue was subjected to column chromatography (petroleum ether: ethyl acetate, 2:1, *v*/*v*) to obtain the product (0.1 g, 60%) as a brown oil. ^1^H NMR (400 MHz, DMSO-*d*_6_) *δ* 11.95 (s, 1H), 9.72 (d, *J* = 6.7 Hz, 2H), 7.77 (t, *J* = 7.9 Hz, 1H), 7.54 (d, *J* = 7.4 Hz, 1H), 7.36 (d, *J* = 8.3 Hz, 1H), 7.09 (d, *J* = 8.4 Hz, 1H), 6.98 (s, 1H), 6.40 (s, 1H), 6.32 (d, *J* = 8.3 Hz, 1H). ^13^C NMR (100 MHz, DMSO-*d*_6_) δ 189.1, 184.1, 167.1, 160.4, 160.0, 156.7, 147.1, 141.4, 136.6, 135.8, 132.2, 131.9, 131.4, 126.9, 123.8, 120.7, 117.8, 117.7, 115.3, 111.2, 106.6, 102.6.

#### 3.1.7. Preparation of Compounds **II-1l** and **II-1m**

Substrate **II** (0.80 g), silver nitrate (0.3 equiv.), ammonium persulfate (3 equiv.), solvent PhCF_3_:H_2_O = 1:1 (30 mL) and boric acid (2 equiv.) were added to a 50 mL round bottom flask. The mixture was stirred at room temperature for 8 h, monitored by TLC, quenched with water (50 mL) and the resulting mixture was extracted with dichloromethane (30 mL × 3). The combined organic layer was washed with brine, dried over anhydrous Na_2_SO_4_ and then concentrated. The residue was subjected to column chromatography (petroleum ether: ethyl acetate, 8:1, *v*/*v*) to obtain the corresponding product.

**II-1l** (8-Hydroxy-2-(*p*-tolyl)naphthalene-1,4-dione): Orange oil, yield 11%. ^1^H NMR (400 MHz, CDCl_3_) *δ* 12.18 (s, 1H), 7.65 (d, *J* = 4.8 Hz, 2H), 7.47 (d, *J* = 7.9 Hz, 2H), 7.30 (d, *J* = 7.2 Hz, 3H), 7.01 (s, 1H), 2.43 (s, 3H). ^13^C NMR (100 MHz, CDCl_3_) *δ* 190.1, 184.4, 162.0, 148.3, 140.7, 136.6, 135.7, 132.2, 129.8, 129.4, 129.3, 124.6, 118.7, 21.4.

**II-1m** (8-Hydroxy-2-(4-methylcyclohexyl)naphthalene-1,4-dione): Orange oil, yield 23%. ^1^H NMR (400 MHz, CDCl_3_) *δ* 11.99 (s, 1H), 7.65–7.58 (m, 2H), 7.25 (s, 1H), 6.69 (s, 1H), 2.90 (t, *J* = 11.9 Hz, 1H), 1.85 (d, *J* = 10.4 Hz, 6H), 1.45 (d, *J* = 13.2 Hz, 2H), 1.28–1.16 (m, 4H). ^13^C NMR (100 MHz, CDCl_3_) *δ* 190.9, 184.1, 161.1, 157.8, 136.1, 132.9, 132.4, 124.0, 119.4, 114.9, 36.8, 32.3, 32.2, 26.4, 26.0. HRMS C_17_H_19_O_3_ [M + H]^+^ cald. 271.1329, found 271.1333.

#### 3.1.8. Preparation of Compounds **III-1b**–**III-1e**

A mixture of **III** (0.6 mmol), corresponding substituted aniline (1 equiv.), Pd(PPh_3_)_4_ (0.1 equiv.) and triethylamine (1 equiv.) in tetrahydrofuran (50 mL) was reacted under argon at 60 °C for 12 h, and the tetrahydrofuran was removed. The residue was taken into water (100 mL) and extracted with dichloromethane (50 mL × 2). The organic layer was washed with brine, dried over anhydrous Na_2_SO_4_ and concentrated. The residue was subjected to column chromatography (petroleum ether: ethyl acetate, 5:1, *v*/*v*) to obtain the corresponding product.

**III-1b** (2-((2,5-Dimethoxyphenyl)amino)-8-hydroxynaphthalene-1,4-dione): Red brown crystals, yield 50%, mp 65–67 °C. ^1^H NMR (400 MHz, CDCl_3_) *δ* 12.92 (s, 1H), 8.16 (s, 1H), 7.69 (dd, *J* = 7.5, 1.0 Hz, 1H), 7.57–7.52 (m, 1H), 7.31 (dd, *J* = 4.2, 1.1 Hz, 1H), 7.03 (d, *J* = 2.9 Hz, 1H), 6.91 (d, *J* = 9.0 Hz, 1H), 6.70 (dd, *J* = 8.9, 2.9 Hz, 1H), 6.44 (s, 1H), 3.90 (s, 3H), 3.83 (s, 3H). ^13^C NMR (100 MHz, CDCl_3_) *δ* 189.9, 184.2, 161.0, 153.8, 145.5, 144.4, 134.3, 130.4, 127.2, 125.9, 119.4, 111.8, 110.1, 109.5, 108.2, 102.9, 56.2, 55.9. HRMS C_18_H_16_NO_5_ [M + H]^+^ cald. 326.1023, found 326.1021.

**III-1c** (2-((3-(*Tert*-butyl)phenyl)amino)-8-hydroxynaphthalene-1,4-dione): Red brown crystals, yield 70%, mp 120–122 °C. ^1^H NMR (400 MHz, CDCl_3_) *δ* 12.91 (d, *J* = 2.3 Hz, 1H), 7.73–7.65 (m, 2H), 7.55–7.50 (m, 1H), 7.41–7.35 (m, 1H), 7.30 (s, 2H), 7.24 (s, 1H), 7.13 (d, *J* = 7.6 Hz, 1H), 6.27 (d, *J* = 2.3 Hz, 1H), 1.35 (d, *J* = 2.4 Hz, 9H). ^13^C NMR (100 MHz, CDCl_3_) *δ* 189.9, 181.6, 161.0, 153.4, 145.7, 136.7, 134.4, 134.3, 130.3, 129.4, 126.1, 123.3, 120.2, 119.9, 119.4, 102.2, 34.9, 31.3. HRMS C_20_H_20_NO_3_ [M + H]^+^ cald. 322.1438, found 322.1435.

**III-1d** (2-((3-(*Tert*-butyl)phenyl)amino)-8-hydroxynaphthalene-1,4-dione): Red brown crystals, yield 80%, mp 175–177 °C. ^1^H NMR (400 MHz, CDCl_3_) *δ* 12.89 (s, 1H), 7.73 (d, *J* = 5.4 Hz, 1H), 7.66 (d, *J* = 7.5 Hz, 1H), 7.52 (t, *J* = 7.9 Hz, 1H), 7.33–7.27 (m, 2H), 7.10–7.04 (m, 3H), 6.30 (s, 1H), 2.39 (s, 3H). ^13^C NMR (100 MHz, CDCl_3_) *δ* 189.9, 181.4, 161.0, 145.5, 139.9, 137.0, 134.3, 129.6, 126.9, 126.1, 123.3, 119.8, 119.4, 102.3, 21.5. HRMS C_17_H_14_NO_3_ [M + H]^+^ cald. 280.0968, found 280.0968.

**III-1e** (2-((4-(*Tert*-butyl)phenyl)amino)-8-hydroxynaphthalene-1,4-dione): Red brown crystals, yield 70%, mp 130–132 °C. ^1^H NMR (400 MHz, CDCl_3_) *δ* 12.92 (s, 1H), 7.67 (d, *J* = 6.8 Hz, 2H), 7.52 (t, *J* = 8.0 Hz, 1H), 7.45 (d, *J* = 8.5 Hz, 2H), 7.29 (s, 1H), 7.21 (d, *J* = 8.5 Hz, 2H), 6.27 (s, 1H), 1.34 (s, 9H). ^13^C NMR (100 MHz, CDCl_3_) *δ* 189.8, 181.4, 161.0, 151.0, 145.7, 134.2, 130.3, 126.7, 126.1, 122.6, 119.3, 114.8, 106.2, 102.1, 34.7, 31.3. HRMS C_20_H_20_NO_3_ [M + H]^+^ cald. 322.1438, found 322.1437.

#### 3.1.9. Preparation of Compound **III-1f**

A mixture of compound **III** (0.6 mmol), phenol (1 equiv.) and potassium carbonate (1 equiv.) in *N,N*-dimethylformamide (10 mL) was stirred at room temperature overnight, quenched with water (120 mL) and extracted with dichloromethane (30 mL × 3). The organic layer was washed with brine, dried over anhydrous Na_2_SO_4_ and then concentrated. The residue was subjected to column chromatography (petroleum ether: ethyl acetate, 5:1, *v*/*v*) to obtain the product as a reddish-brown oil (0.1 g, 60%). ^1^H NMR (400 MHz, CDCl_3_) *δ* 11.80 (s, 1H), 7.62 (dd, *J* = 16.3, 7.5 Hz, 2H), 7.48 (t, *J* = 7.7 Hz, 2H), 7.36–7.28 (m, 2H), 7.14 (d, *J* = 7.9 Hz, 2H), 5.94 (s, 1H). ^13^C NMR (100 MHz, CDCl_3_) *δ* 184.8, 184.1, 162.0, 160.2, 152.6, 137.3, 132.0, 130.5, 126.8, 124.1, 121.0, 119.0, 114.4, 114.0.

### 3.2. Biological Assay

Each test was repeated 2–3 times at 25 ± 1 °C. Active effect was expressed in a percentage scale of 0–100 (0: no activity; 100: total inhibited).

Specific steps for the fungicidal activity, anti-TMV activity and insecticidal activity tests were carried out using a literature method [20,21], which also can be seen in the Supplementary Materials.

## 4. Conclusions

In summary, using natural products plumbagin (**I**) and juglone (**II**) as the parent structures, a series of quinone derivatives were designed, synthesized and systematically evaluated for their fungicidal activities, antiviral activities and insecticidal activities. These compounds were found to have broad-spectrum bio-activities and showed different structure-activity relationships to different pathogens and pests. For *Cercospora arachidicola Hori*: the natural product plumbagin (**I**) displayed excellent fungicidal activity, and the demethylation in the 2-position of **I** or introduction of bromine at the 3-position of **II** led to extremely reduced activity; the overall activities of the derivatives containing bromine at the 3-position of **II** (**III** and **III-1a**) were poor, indicating that the introduction of bromine atom at the 3-position of **II** is very unfavorable to the activity; finally, compounds **I**, **I-1e** and **II-1a** with significantly higher fungicidal activities than pyrimethanil emerged as new antifungal lead compounds for further research. For TMV: the introduction of benzenesulfonyl on the 5-hydroxyl was favorable to the antiviral activities of these compounds; **I-1f** and **II-1f** with excellent anti-TMV activities emerged as new antiviral candidates. For *Plutella xylostella*: the introduction of *p*-methylbenzoyl, cyclopropyl, phenylpropionyl, *n*-hexyl or benzyl onto the 5-hydroxyl of juglone (**II**) was beneficial to the improvement of activity; **II-1d** and **III-1c** with a similar level of larvacidal activities as hexaflumuron can be used as new insecticide candidates. During in vivo anti-TMV activity testing, we found that these compounds were safe for tobacco leaves at a concentration of 500 µg/mL. The fungicidal and insecticidal activities are currently at the stage of in vitro testing and do not involve the tested plants. After identifying promising compounds, the field trials will be conducted and then the toxicity studies on bees, birds and fish will be carried out to comprehensively evaluate their environmental behavior. This work opens the prelude to the application of naphthoquinone compounds in the field of agricultural protection. We are conducting chemical biological experiments to verify the action mode of these compounds.

## Figures and Tables

**Table 1:** In vitro fungicidal activities of compounds **I**, **II**, **III**, **I-1a**–**I-1h**, **II-1a**–**II-1m**, **III-1a**–**III-1f**, chlorothalonil, carbendazim and pyrimethanil against 14 types of fungi ^a^.

Compd	Fungicidal Activities (%) at 50 μg/mL													
	F.O	C.H	P.P	R.C	B.M	W.A	F.M	A.S	F.G	P.I	P.C	S.S	B.C	R.S
**I**	89 ± 2	100	85 ± 3	37 ± 3	73 ± 3	83 ± 3	90 ± 3	31 ± 2	78 ± 3	78 ± 2	100	97 ± 2	83 ± 2	100
**I-1a**	41 ± 3	40 ± 4	50 ± 3	64 ± 4	49 ± 3	50 ± 4	56 ± 3	31 ± 4	44 ± 4	33 ± 4	42 ± 3	34 ± 4	61 ± 1	76 ± 3
**I-1b**	57 ± 3	33 ± 2	36 ± 1	58 ± 3	61 ± 4	52 ± 3	49 ± 4	25 ± 1	36 ± 1	44 ± 1	36 ± 5	17 ± 1	22 ± 1	46 ± 1
**I-1c**	30 ± 1	27 ± 1	46 ± 2	64 ± 3	26 ± 2	43 ± 1	37 ± 2	6 ± 1	50 ± 2	33 ± 2	22 ± 1	28 ± 2	46 ± 2	83 ± 1
**I-1d**	46 ± 2	43 ± 2	14 ± 1	70 ± 1	53 ± 1	47 ± 1	56 ± 1	38 ± 2	50 ± 2	67 ± 2	44 ± 2	35 ± 1	54 ± 3	79 ± 2
**I-1e**	96 ± 2	100	97 ± 2	97 ± 2	78 ± 2	79 ± 2	92 ± 2	50 ± 1	53 ± 2	78 ± 2	67 ± 2	44 ± 2	85 ± 2	100
**I-1f**	20 ± 2	30 ± 2	19 ± 1	50 ± 2	25 ± 1	36 ± 2	36 ± 2	6 ± 1	27 ± 2	11 ± 1	6 ± 1	34 ± 1	47 ± 2	22 ± 2
**I-1g**	26 ± 2	23 ± 2	50 ± 1	57 ± 3	23 ± 1	34 ± 2	48 ± 2	37 ± 2	27 ± 2	33 ± 1	22 ± 1	34 ± 2	52 ± 3	22 ± 1
**I-1h**	64 ± 2	80 ± 2	18 ± 1	74 ± 4	52 ± 2	60 ± 3	80 ± 3	31 ± 2	50 ± 1	55 ± 2	61 ± 2	29 ± 2	54 ± 2	60 ± 3
**II**	25 ± 1	20 ± 1	16 ± 1	23 ± 1	25 ± 1	37 ± 1	43 ± 1	43 ± 2	16 ± 1	33 ± 1	22 ± 1	17 ± 1	30 ± 1	37 ± 2
**II-1a**	66 ± 2	100	63 ± 2	37 ± 2	70 ± 3	75 ± 2	80 ± 1	25 ± 1	44 ± 2	33 ± 2	63 ± 3	32 ± 2	39 ± 2	63 ± 2
**II-1b**	62 ± 3	70 ± 2	29 ± 2	57 ± 2	54 ± 3	44 ± 2	65 ± 3	56 ± 1	67 ± 2	66 ± 2	75 ± 2	48 ± 1	32 ± 2	87 ± 4
**II-1c**	67 ± 2	73 ± 2	44 ± 1	82 ± 3	78 ± 3	58 ± 3	61 ± 2	56 ± 2	64 ± 2	89 ± 2	77 ± 3	62 ± 3	28 ± 1	69 ± 2
**II-1d**	42 ± 2	50 ± 1	36 ± 1	61 ± 3	41 ± 2	55 ± 2	61 ± 3	18 ± 1	27 ± 1	55 ± 1	77 ± 2	39 ± 2	37 ± 2	72 ± 1
**II-1e**	75 ± 2	83 ± 2	48 ± 2	71 ± 3	60 ± 2	58 ± 1	63 ± 2	18 ± 1	33 ± 2	44 ± 2	66 ± 2	29 ± 1	54 ± 2	66 ± 3
**II-1f**	26 ± 1	23 ± 1	22 ± 1	16 ± 1	21 ± 2	37 ± 2	36 ± 3	31 ± 2	19 ± 1	22 ± 1	13 ± 1	11 ± 1	17 ± 2	30 ± 2
**II-1g**	26 ± 1	33 ± 2	8 ± 1	78 ± 4	37 ± 2	43 ± 2	51 ± 2	43 ± 3	25 ± 2	33 ± 1	38 ± 2	34 ± 1	32 ± 2	51 ± 3
**II-1h**	26 ± 1	26 ± 1	89 ± 2	50 ± 2	25 ± 1	37 ± 2	29 ± 3	6 ± 1	22 ± 2	33 ± 2	27 ± 2	46 ± 2	21 ± 1	27 ± 1
**II-1i**	9 ± 1	13 ± 1	29 ± 1	23 ± 2	10 ± 1	36 ± 2	22 ± 2	18 ± 1	11 ± 1	11 ± 1	19 ± 1	34 ± 2	21 ± 2	30 ± 2
**II-1j**	23 ± 1	30 ± 2	29 ± 2	71 ± 2	23 ± 2	37 ± 3	17 ± 1	12 ± 1	27 ± 2	22 ± 2	22 ± 3	72 ± 3	50 ± 4	48 ± 4
**II-1k**	44 ± 2	53 ± 3	78 ± 2	37 ± 2	43 ± 3	51 ± 2	58 ± 1	31 ± 2	41 ± 1	44 ± 2	75 ± 2	46 ± 2	45 ± 3	60 ± 4
**II-1l**	32 ± 2	46 ± 2	85 ± 2	37 ± 1	37 ± 2	55 ± 3	56 ± 3	43 ± 2	44 ± 2	22 ± 1	22 ± 1	61 ± 2	43 ± 3	71 ± 2
**II-1m**	19 ± 2	40 ± 1	90 ± 2	34 ± 2	51 ± 2	60 ± 3	53 ± 3	25 ± 2	41 ± 1	78 ± 2	19 ± 1	54 ± 2	56 ± 2	68 ± 3
**III**	12 ± 1	20 ± 2	26 ± 1	53 ± 2	25 ± 1	32 ± 1	34 ± 3	12 ± 1	17 ± 1	11 ± 1	13 ± 1	23 ± 2	21 ± 2	38 ± 2
**III-1a**	23 ± 2	30 ± 2	31 ± 2	78 ± 2	39 ± 3	37 ± 3	58 ± 4	18 ± 1	22 ± 2	22 ± 1	27 ± 2	11 ± 1	37 ± 2	21 ± 1
**III-1b**	17 ± 1	23 ± 2	36 ± 2	27 ± 1	29 ± 2	34 ± 3	7 ± 1	12 ± 1	22 ± 2	22 ± 1	22 ± 3	34 ± 3	52 ± 3	53 ± 4
**III-1c**	14 ± 1	20 ± 1	30 ± 3	21 ± 2	17 ± 1	34 ± 2	29 ± 2	6 ± 2	17 ± 2	11 ± 2	11 ± 2	23 ± 1	26 ± 2	30 ± 3
**III-1d**	16 ± 1	80 ± 2	22 ± 2	23 ± 2	29 ± 2	34 ± 3	36 ± 3	12 ± 1	11 ± 1	11 ± 1	5 ± 1	23 ± 2	43 ± 2	22 ± 3
**III-1e**	9 ± 1	17 ± 2	33 ± 2	23 ± 2	15 ± 1	29 ± 2	24 ± 1	25 ± 1	13 ± 2	11 ± 2	11 ± 1	17 ± 1	30 ± 2	22 ± 2
**III-1f**	37 ± 2	40 ± 2	68 ± 3	23 ± 3	43 ± 1	43 ± 2	61 ± 3	12 ± 1	33 ± 2	55 ± 2	61 ± 2	23 ± 1	52 ± 2	68 ± 3
Chlorothalonil ^b^	95 ± 2	19 ± 1	98 ± 2	98 ± 1	97 ± 1	98 ± 1	83 ± 3	38 ± 2	100	73 ± 2	88 ± 2	100	92 ± 2	100
Carbendazim ^b^	100	28 ± 1	98 ± 2	98 ± 1	97 ± 1	98 ± 2	90 ± 3	13 ± 1	100	48 ± 2	44 ± 1	100	42 ± 1	100
Pyrimethanil ^b^	19 ± 1	83 ± 2	71 ± 3	84 ± 2	28 ± 1	21 ± 1	27 ± 1	88 ± 2	59 ± 1	79 ± 3	100	100	100	100

^a^ Average of three replicates; all results are expressed as mean ± SD. Abbreviations: F.O, *Fusarium oxysporum* f. sp. *cucumeris*; C.H, *Cercospora arachidicola Hori*; P.P, *Physalospora piricola*; R.C, *Rhizoctonia cerealis*; B.M, *Bipolaris maydis*; W.A, *Watermelon anthracnose*; F.M, *Fusarium moniliforme*; A.S, *Alternaria solani*; F.G, *Fusarium graminearum*; P.I, *Phytophthora infestans*; P.C, *Phytophthora capsici*; S.S, *Sclerotinia sclerotiorum*; B.C, *Botrytis cinereal*; R.S, *Rhizoctonia solani*. Activity data with prominence are presented in blue or red. ^b^ The commercial agricultural fungicides were used for comparison of antifungal activity.

**Table 2:** EC_50_ values of compounds **I**, **I-1e**, **II-1a** and pyrimethanil against *Cercospora arachidicola Hori*.

Compd.	Regression Equation	*r* ^2^	Protection Effect EC_50_ (µg/mL)
**I**	y = 2.71 + 1.99x	0.9808	17.70
**I-1e**	y = 2.64 + 2.29x	0.9987	11.35
**II-1a**	y = 2.78 + 2.18x	0.9861	12.56
Pyrimethanil	y = 2.78 + 1.84x	0.9885	19.17

**Table 3:** In vivo antiviral activities of compounds **I**, **II**, **III**, **I-1a**–**I-1h**, **II-1a**–**II-1m**, **III-1a**–**III-1f** and ribavirin against TMV ^a^.

Compd.	Concn (μg/mL)	Inactive Effect (%)	Curative Effect (%)	Protective Effect (%)
**I**	500	13 ± 3	**–**	**–**
**I-1a**	500	15 ± 2	**–**	**–**
**I-1b**	500	26 ± 3	**–**	**–**
**I-1c**	500	4 ± 1	**–**	**–**
**I-1d**	500	24 ± 1	**–**	**–**
**I-1e**	500	10 ± 1	**–**	**–**
**I-1f**	500	43 ± 4	37 ± 2	36 ± 2
	100	7 ± 1	9 ± 2	0
**I-1g**	500	19 ± 4	**–**	**–**
**I-1h**	500	14 ± 3	**–**	**–**
**II**	500	18 ± 3	**–**	**–**
**II-1a**	500	16 ± 2	**–**	**–**
**II-1b**	500	14 ± 4	**–**	**–**
**II-1c**	500	16 ± 4	**–**	**–**
**II-1d**	500	0	**–**	**–**
**II-1e**	500	18 ± 1	**–**	**–**
**II-1f**	500	44 ± 2	36 ± 2	39 ± 3
	100	5 ± 1	8 ± 1	10 ± 2
**II-1g**	500	37 ± 5	**–**	**–**
**II-1h**	500	0	**–**	**–**
**II-1i**	500	22 ± 2		
**II-1j**	500	25 ± 3	**–**	**–**
**II-1k**	500	28 ± 3	**–**	**–**
**II-1l**	500	35 ± 4	**–**	**–**
**II-1m**	500	32 ± 4	**–**	**–**
**III**	500	24 ± 4	**–**	**–**
**III-1a**	500	8 ± 3	**–**	**–**
**III-1b**	500	39 ± 3	**–**	**–**
	100	6 ± 1	**–**	**–**
**III-1c**	500	17 ± 4	**–**	**–**
**III-1d**	500	35 ± 3	**–**	**–**
	100	3 ± 1	**–**	**–**
**III-1e**	500	26 ± 1	**–**	**–**
**III-1f**	500	31 ± 1	**–**	**–**
Ribavirin ^b^	500	38 ± 2	36 ± 2	40 ± 3
	100	11 ± 1	13 ± 1	11 ± 2

^a^ Average of three replicates; All results are expressed as mean ± SD; Activity data with prominence are presented in blue; “**–**”: not tested. ^b^ The commercial agricultural antiviral agent were used for comparison of antiviral activity.

**Table 4:** Insecticidal activities of compounds **I**, **II**, **III**, **I-1a**–**I-1h**, **II-1a**–**II-1m**, **III-1a**–**III-1f**, matrine, hexaflumuron and rotenone against eight common crop pests ^a^.

Compd.	Insecticidal Activities (%)/ Concn (μg/mL)												
	M.S		H.A	S.F	O.N	C.P	A.C	T.C		P.X			
	600	200	600	600	600	10	600	600	200	600	200	100	10
**I**	100	50 ± 0	35 ± 5	30 ± 10	40 ± 0	60 ± 0	0	0	**–**	90 ± 10	60 ± 10	**–**	**–**
**I-1a**	0	**–**	0	0	0	0	0	50 ± 0	**–**	70 ± 10	**–**	**–**	**–**
**I-1b**	50 ± 10	**–**	20 ± 0	20 ± 0	0	0	0	0	**–**	30 ± 0	**–**	**–**	**–**
**I-1c**	40 ± 10	**–**	15 ± 5	10 ± 0	20 ± 0	0	0	0	**–**	80 ± 10	**–**	**–**	**–**
**I-1d**	30 ± 10	**–**	10 ± 0	0	0	0	0	0	**–**	0	**–**	**–**	**–**
**I-1e**	10 ± 0	**–**	0	0	0	0	0	0	**–**	55 ± 5	**–**	**–**	**–**
**I-1f**	10 ± 0	**–**	10 ± 0	10 ± 0	15 ± 5	0	0	0	**–**	70 ± 10	**–**	**–**	**–**
**I-1g**	20 ± 10	**–**	25 ± 5	0	0	0	0	0	**–**	85 ± 5	65 ± 5	**–**	**–**
**I-1h**	0	**–**	0	0	0	0	0	100	80 ± 10	80 ± 0	**–**	**–**	**–**
**II**	50 ± 10	**–**	15 ± 5	0	0	0	0	0	**–**	85 ± 5	55 ± 5	**–**	**–**
**II-1a**	10 ± 0	**–**	0	0	15 ± 5	0	0	0	**–**	90 ± 0	70 ± 10	**–**	**–**
**II-1b**	0	**–**	0	0	0	0	40 ± 10	75 ± 5	0	100	70 ± 0	**–**	**–**
**II-1c**	30 ± 0	**–**	0	0	0	0	0	0	**–**	0	**–**	**–**	**–**
**II-1d**	0	**–**	0	0	0	0	0	0	**–**	100	100	85 ± 5	60 ± 0
**II-1e**	0	**–**	0	0	0	0	0	75 ± 5	0	80 ± 10	45 ± 5	**–**	**–**
**II-1f**	30 ± 0	**–**	15 ± 5	10 ± 0	25 ± 5	0	50 ± 0	0	**–**	70 ± 0	**–**	**–**	**–**
**II-1g**	0	**–**	0	0	10 ± 0	0	0	0	**–**	75 ± 5	**–**	**–**	**–**
**II-1h**	30 ± 0	**–**	20 ± 10	20 ± 0	40 ± 0	0	0	70 ± 10	0	100	85 ± 5	50 ± 0	**–**
**II-1i**	20 ± 10	**–**	0	0	0	0	0	0	**–**	80 ± 10	50 ± 10	**–**	**–**
**II-1j**	20 ± 10	**–**	0	0	0	0	0	100	65 ± 5	90 ± 0	65 ± 5	**–**	**–**
**II-1k**	20 ± 0	**–**	0	0	0	0	0	0	**–**	60 ± 10	**–**	**–**	**–**
**II-1l**	10 ± 0	**–**	15 ± 5	15 ± 5	30 ± 10	0	0	0	**–**	75 ± 5	**–**	**–**	**–**
**II-1m**	15 ± 5	**–**	0	15 ± 5	10 ± 0	0	0	0	**–**	60 ± 10	**–**	**–**	**–**
**III**	30 ± 10	**–**	0	15 ± 5	0	0	0	0	**–**	40 ± 10	**–**	**–**	**–**
**III-1a**	10 ± 0	**–**	0	0	0	0	0	0	**–**	70 ± 10	**–**	**–**	**–**
**III-1b**	20 ± 10	**–**	15 ± 5	15 ± 5	0	0	0	0	**–**	80 ± 10	40 ± 10	**–**	**–**
**III-1c**	0	**–**	0	0	0	0	45 ± 5	60 ± 0	**–**	100	100	70 ± 0	30 ± 0
**III-1d**	30 ± 10	**–**	0	15 ± 5	0	0	0	50 ± 0	**–**	100	75 ± 5	**–**	**–**
**III-1e**	30 ± 0	**–**	10 ± 0	0	0	0	0	0	**–**	75 ± 5	**–**	**–**	**–**
**III-1f**	0	**–**	0	0	0	40 ± 10	0	0	**–**	85 ± 5	50 ± 0	**–**	**–**
Matrine ^b^	80 ± 10	45 ± 5	45 ± 5	50 ± 0	35 ± 5	25 ± 5	35 ± 5	55 ± 5	50 ± 0	35 ± 5	30 ± 0	25 ± 5	15 ± 5
Hexaflumuron ^b^	100	100	100	100	45 ± 5	55 ± 5	45 ± 5	100	75 ± 5	100	95 ± 5	85 ± 5	55 ± 5
Rotenone ^b^	100	35 ± 5	50 ± 10	55 ± 5	95 ± 5	100	100	35 ± 5	50 ± 0	100	35 ± 5	20 ± 0	20 ± 0

^a^ Average of three replicates; all results are expressed as mean ± SD. Abbreviations: M.S, *Mythimna separata*; H.A, *Helicoverpa armigera*; S.F, *Spodoptera frugiperda*; O.N, *Ostrinia nubilalis*; C.P, *Culex pipiens*; A.C, *Aphis craccivora*; T.C, *Tetranychus cinnabarinus*; P.X, *Plutella xylostella*; Activity data with prominence are presented in blue or red; “**–**”: not tested. ^b^ The commercial agricultural insecticides were used for comparison of insecticidal activity.

## Data Availability

All data used to support the findings of this study are included within the article and Supplementary Materials.

## Supplementary Materials

The following supplementary materials can be downloaded at: https://www.mdpi.com/article/10.3390/molecules28083328/s1, Section S1: detailed bio-assay procedures for the fungicidal activity, anti-TMV activity and insecticidal activity; Section S2: copies of ^1^H & ^13^C NMR spectra (Figures S1–S58). References [26,27,28,29,30,31,32,33,34] were cited in supplementary materials.
