# Supplementary material for: Natural Products for Pesticides Discovery: Structural Diversity Derivation and Biological Activities of Naphthoquinones Plumbagin and Juglone
Source: Molecules. 2023 Apr 9;28(8):3328. doi: 10.3390/molecules28083328 (PMC10141837; doi:10.3390/molecules28083328)
Supplement: Supplementary file 1 [file molecules-28-03328-s001.zip › molecules-2279188-supplementary.pdf]

Supplementary Materials

Natural products for pesticides discovery: Structural diversity derivation and biological activities of naphthoquinones plumbagin and juglone

Kaihua Wang[1], Beibei Wang[1], Henan Ma[1], Ziwen Wang[2,*], Yuxiu Liu[1], Qingmin Wang[1,*]

[1]State Key Laboratory of Elemento-Organic Chemistry, Research Institute of Elemento-Organic Chemistry, College of Chemistry, Frontiers Science Center for New Organic Matter, Nankai University, Tianjin 300071, China;

[2]Tianjin Key Laboratory of Structure and Performance for Functional Molecules, College of Chemistry, Tianjin Normal University, Tianjin 300387, China.

Ziwen Wang, Tel: 0086-22-23766531; Fax: 0086-22-23766531; e-mail: hxxywzw@tjnu.edu.cn;

Qingmin Wang, Tel.: 0086-22-23503952; Fax: 0086-22-23503952; e-mail: wangqm@nankai.edu.cn.

Contents

1. Biological Assay S3

2. Reference: S6

3. Spectra of all target compounds, intermediates S7

1. Biological Assay

Detailed procedures for determining fungicidal, antiviral and insecticidal activities were described in our and other researchers' published literature.[1-9] According to statistical requirements, each bioassay was repeated 2-3 times.

Detailed bio-assay procedures

Fungicidal activities[1]

The fungicidal activities of compounds were evaluated in mycelial growth tests conducted in artificial media against 14 plant pathogens at a rate of 50 μg/mL. Each test compound was dissolved in a suitable amount of acetone and diluted with water containing 0.1% TW-80 to a concentration of 500 μg/mL. To each petri dish was added 1 mL of the test solution and 9 mL of culture medium to make a 50 μg/mL concentration of the test compound, while in another petri dish was added 1 mL distilled water containing 0.1% TW-80 and 9 mL of culture medium as a blank control. A 4 mm diameter of hyphal growth was cut using a hole puncher on a growing fungal S6 culture and the hyphae were moved to the petri dish containing the test compound. Each assay was performed three times. The dishes were stored in controlled environment cabinets (24±1°C) for 4 days, after which the diameter of mycelial growth was measured and the percentage inhibition was calculated using the following equation: Percentage inhibition (%) = (averaged diameter of mycelia in blank controls – averaged diameter of mycelia in medicated tablets) / (averaged diameter of mycelia in blank controls – 4 mm) × 100.

Antiviral Biological Assay.[2]

Purification of Tobacco Mosaic Virus.

Using Gooding's method[3], the upper leaves of *Nicotiana tabacum* L. inoculated with TMV were selected and ground in phosphate buffer and then filtered through double-layer pledget. The filtrate

was centrifuged at 10000 g, treated with PEG twice, and centrifuged again. The whole experiment was processed at 4 °C. Absorbance value was estimated at 260 nm by ultraviolet spectrophotometer.

$$Virus\ concn = (A_{260} \times dilution\ ratio)E\,/^{0.1\%,\,260nm}_{1cm}$$

Protective Effect of Compounds against TMV in Vivo.

The compound solution was smeared on growing *Nicotiana. tabacum* L. leaves (at least 3 leaves) of the same age. In another pot, the leaves were smeared with the solvent as a control. After 12 h, the leaves were inoculated with TMV with the juice-leaf rubbing method and then washed with water. The total local lesion numbers appearing on the leaves 3−4 days after inoculation were recorded.[4] There are three replicates for each compound.

Inactivation Effect of Compounds against TMV in Vivo.

To test viral inhibition, equal volumes of the virus and the compound solution were mixed together for 30 min. The mixture was then inoculated into the growing *N. tabacum* L leaves of the same age, and another pot was inoculated with the mixture of solvent and the virus as the control. The local lesion numbers were recorded 3−4 days after inoculation.[5] There are three replicates for each compound.

Curative Effect of Compounds against TMV in Vivo.

TMV (concentration of 6.0×10^{-3} μg/mL) was inoculated on the growing leaves of *N. tabacum* L. of the same age. Then, the leaves were washed with water and dried. The compound solution was smeared on the inoculated leaves, while inoculated leaves in another pot were smeared with the solvent as a control. The local lesion numbers were recorded 3−4 days after inoculation.[6] There are three replicates for each compound. The in vitro and in vivo inhibition rates of the compound were then calculated according to the following formula ("av" means average, and controls were not

treated with compound).

Inhibition rate (%) = [(av local lesion no. of control − av local lesion no. of drug-treated)/av local lesion no. of control] × 100%

Detailed bioassay procedures for the insecticidal activities[7-9]

Larvicidal Activities against oriental armyworm (Mythimna separata), cotton bollworm (Helicoverpa armigera), fall armyworm (Spodoptera frugiperda), corn borer (Ostrinia nubilalis), Bean aphid (Aphis craccivora), Spider Mite (Tetranychus cinnabarinus), diamondback moth (Plutella xylostella):

Stock solutions of each tested compound was prepared in dimethylformamide at a concentration of 600 mg L^{-1} and then diluted to the required concentration (200, 100, 10 mg L^{-1}) with water containing TW-20.

Leaf-dip method was used. Leaf discs (5 cm × 3 cm) were cut from fresh cabbage leaves (or other leaves) and then dipped into the test solution for 3 s. After air-drying, the treated leaf discs were placed individually into vertical tube (or Petri dishes) and the discs were infested with 10 larvae (for example: 10 second-instar diamondback moth larvae, 10 fourth-instar oriental armyworm larvae). Percentage mortalities were evaluated 3 days after treatment.

Evaluations were based on a percentage scale of 0–100, where 0 equals no activity and 100 equals total kill. Each treatment was performed at least three times.

Larvicidal Activities against Culex pipiens:

Twenty fourth-instar *Culex pipiens* were placed into the test solution (10 mg·L^{-1}). Percentage mortalities were evaluated 3 days after treatment. Evaluations were based on a percentage scale of 0–100, where 0 equals no activity and 100 equals total kill. Each treatment was performed at least

three times.

2. Reference:

(1) Zhao, H. P.; Liu, Y. X.; Cui, Z. P.; Beattie, D.; Gu, Y. C.; Wang, Q. M. Design, synthesis, and biological activities of arylmethylamine substituted chlorotriazine and methylthiotriazine compounds. *J. Agric. Food Chem*. **2011**, *59*, 11711−11717.

(2) Wang, Z. W.; Wei, P.; Wang, L. Z.; Wang, Q. M. Design, synthesis, and anti-tobacco mosaic virus (TMV) activity of phenanthroindolizidines and their analogues. *J. Agric. Food Chem*. **2012**, *60*, 10212−10219.

(3) Gooding, G. V., Jr.; Hebert, T. T. A simple technique for purification of tobacco mosaic virus in large quantities. *Phytopathology* **1967**, 57, 1285−1290.

(4) Li, S. Z.; Wang, D. M.; Jiao, S. M. In *Pesticide Experiment Methods-Fungicide Sector*; Li, S. Z., Ed.; Agriculture Press of China: Beijing, China, **1991**; 93−94.

(5) Leberman, R. Isolation of plant viruses by means of simple coacervates. *Virol* **1966**, *30*, 341–347.

(6) Fraenkel Conrat, H.; Williams, R. C. Reconstitution of active tobacco mosaic virus fromits inactive protein and nucleic acid components. *PNAS* U S A **1955**, *41*, 690–698.

(7) Ni, W. J.; Li, C. J.; Liu, Y. X.; Song, H. J.; Wang, L. Z.; Song, H. B.; Wang, Q. M. Various bioactivity and relationship of structure−activity of matrine analogues. *J. Agric. Food Chem.* **2017**, *65*, 2039−2047.

(8) Yu, X.L.; Liu, Y. X.; Li, Y. Q.; Wang, Q. M. Design, synthesis, acaricidal/insecticidal activity, and structure−activity relationship studies of novel oxazolines containing sulfone/sulfoxide groups based on the sulfonylurea receptor protein-binding site. *J. Agric. Food Chem.* **2016**, *64*, 3034−3040.

(9) Yang, Y.; Liu, Y. X.; Song, H. J.; Li, Y. Q.; Wang, Q. M. Additive effects on the improvement of insecticidal activity: Design, synthesis, and insecticidal activity of novel pymetrozine derivatives. *Bioorg. Med. Chem.* **2016**, *24*, 391−402.

3. Spectra of all target compounds, intermediates

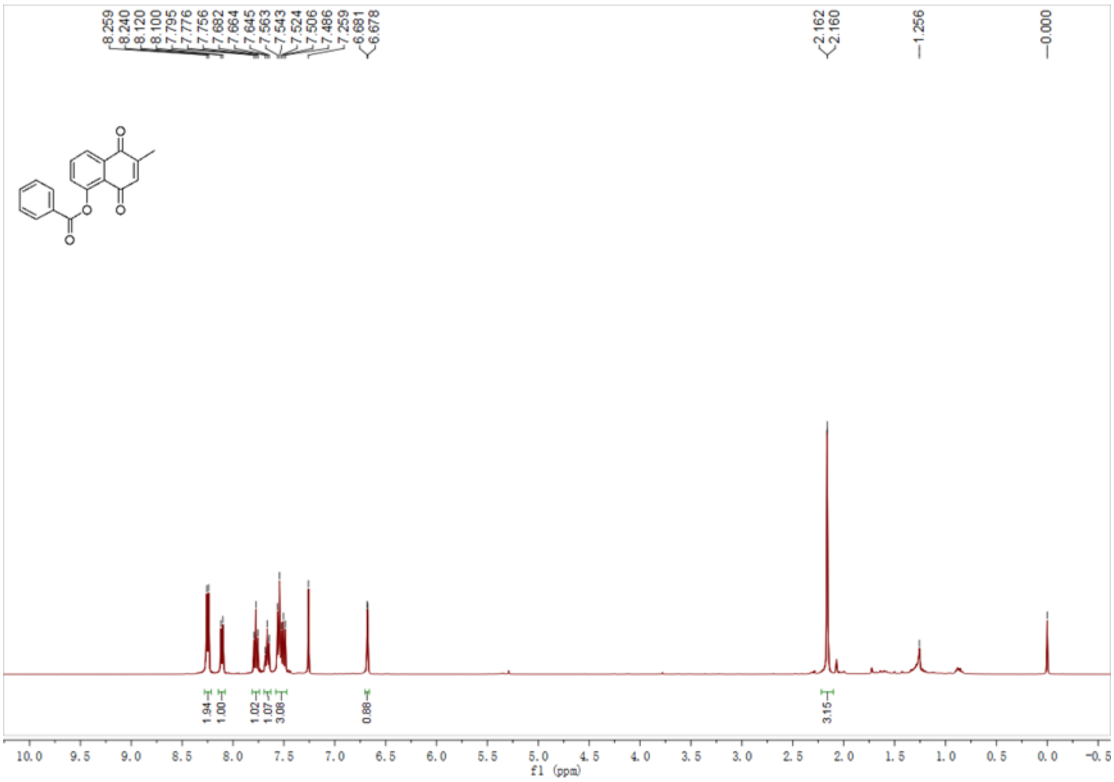

Figure S1. ^{1}H NMR spectrum of **I-1a**

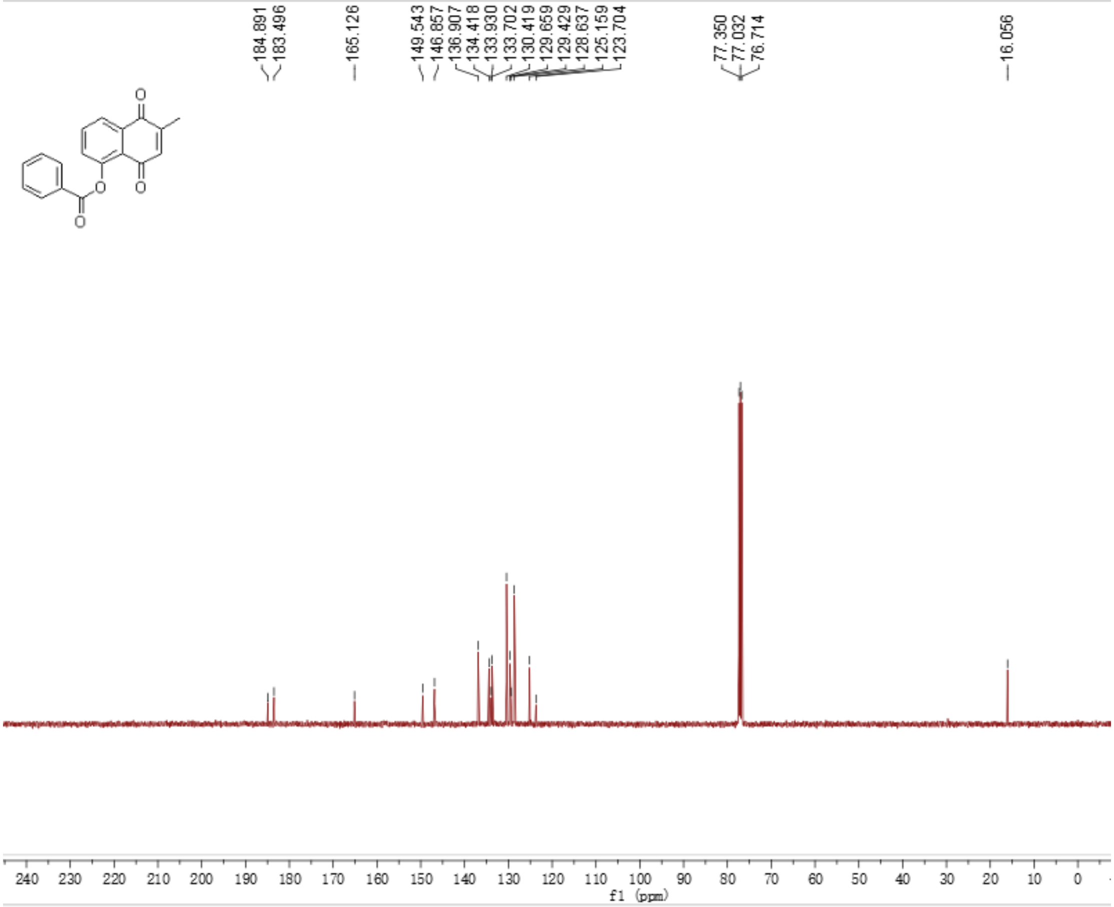

Figure S2. ^{13}C NMR spectrum of **I-1a**

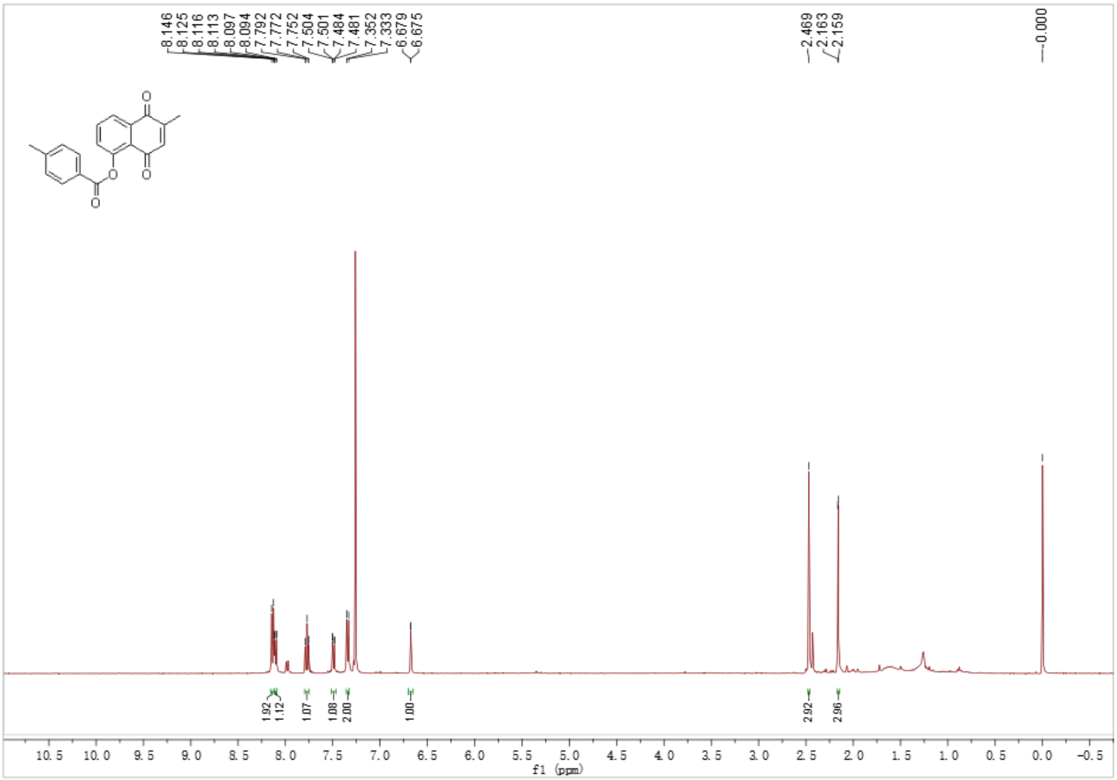

Figure S3. ^{1}H NMR spectrum of **I-1b**

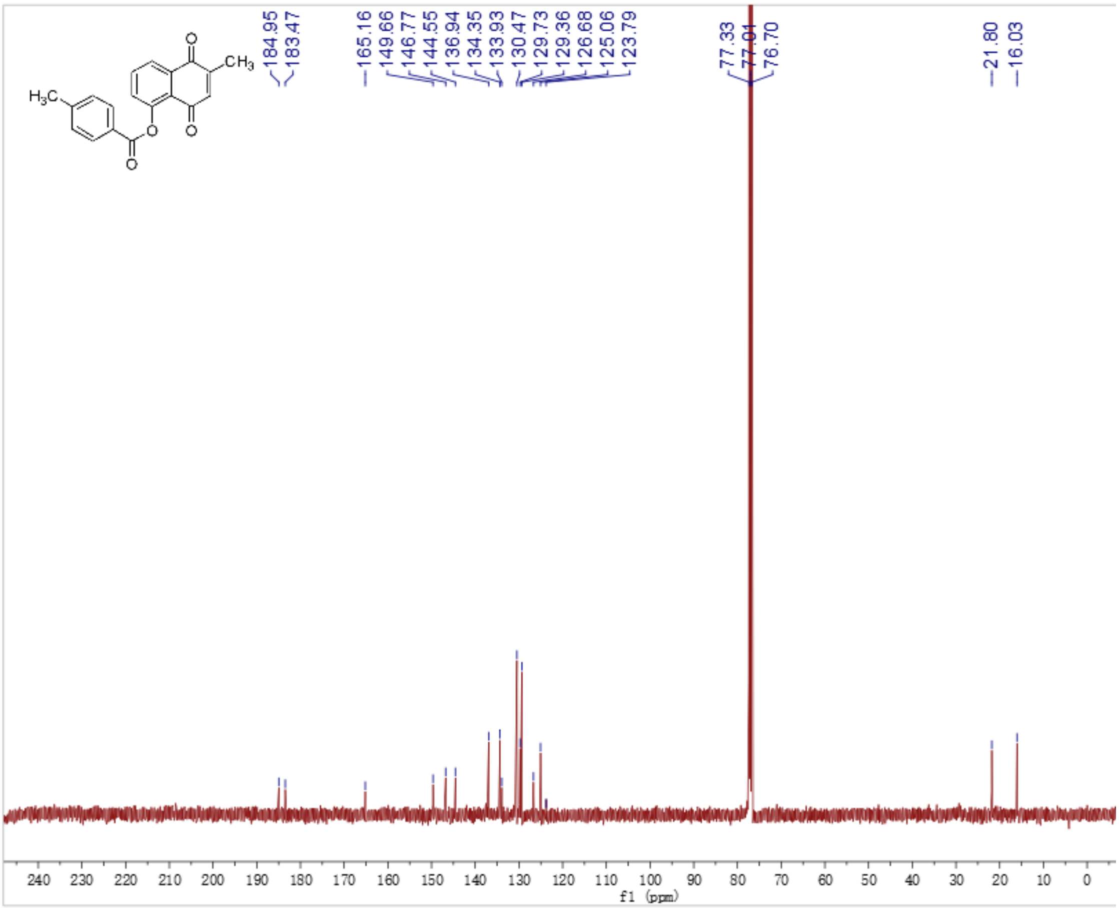

Figure S4. ^{13}C NMR spectrum of **I-1b**

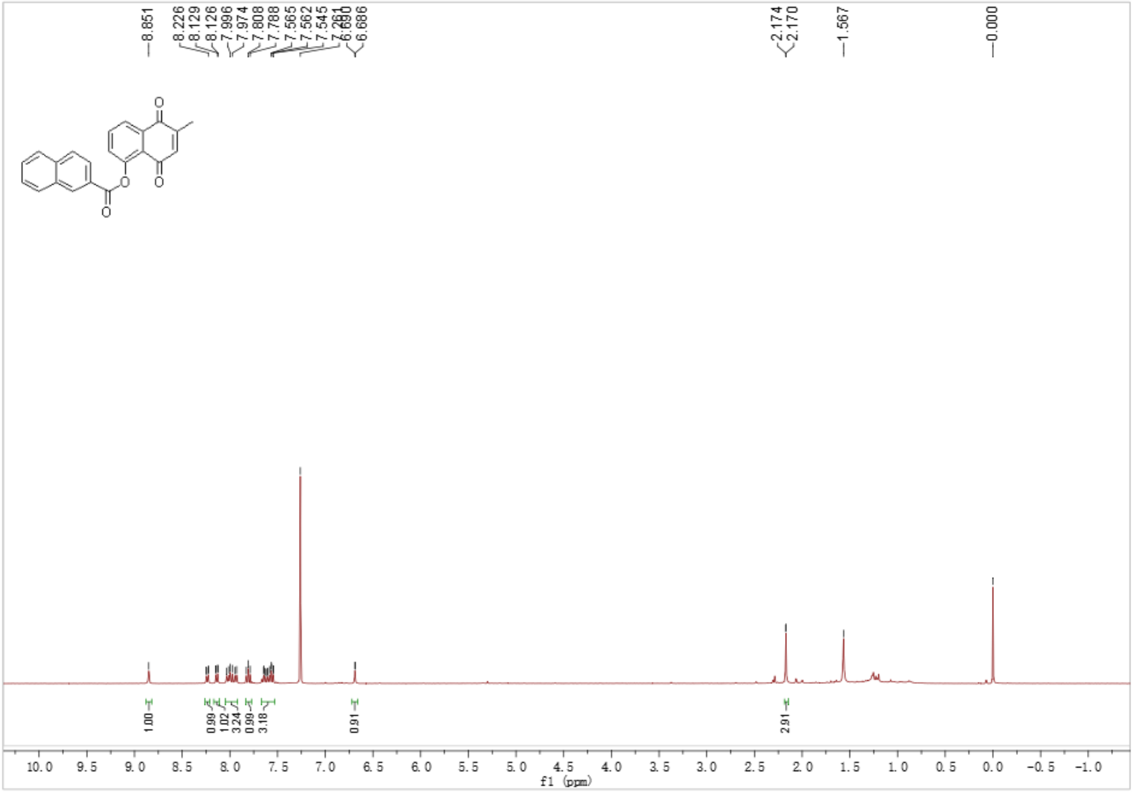

Figure S5. ^{1}H NMR spectrum of **I-1c**

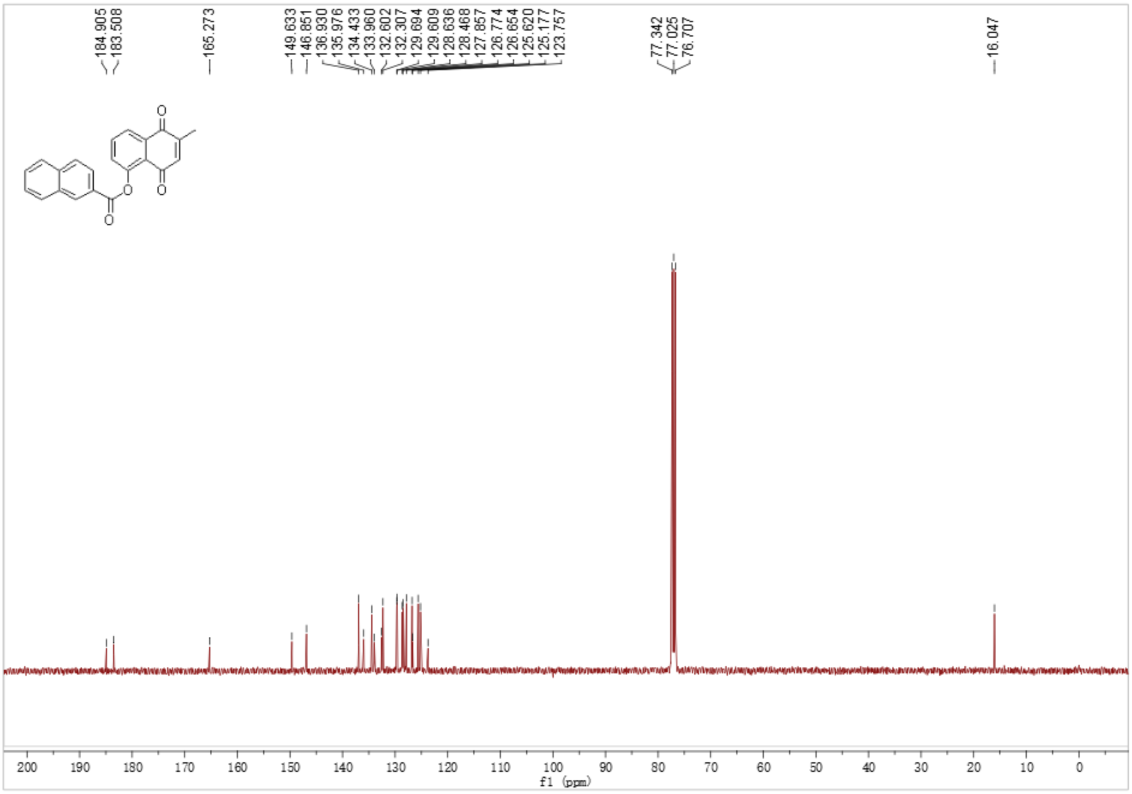

Figure S6. ^{13}C NMR spectrum of **I-1c**

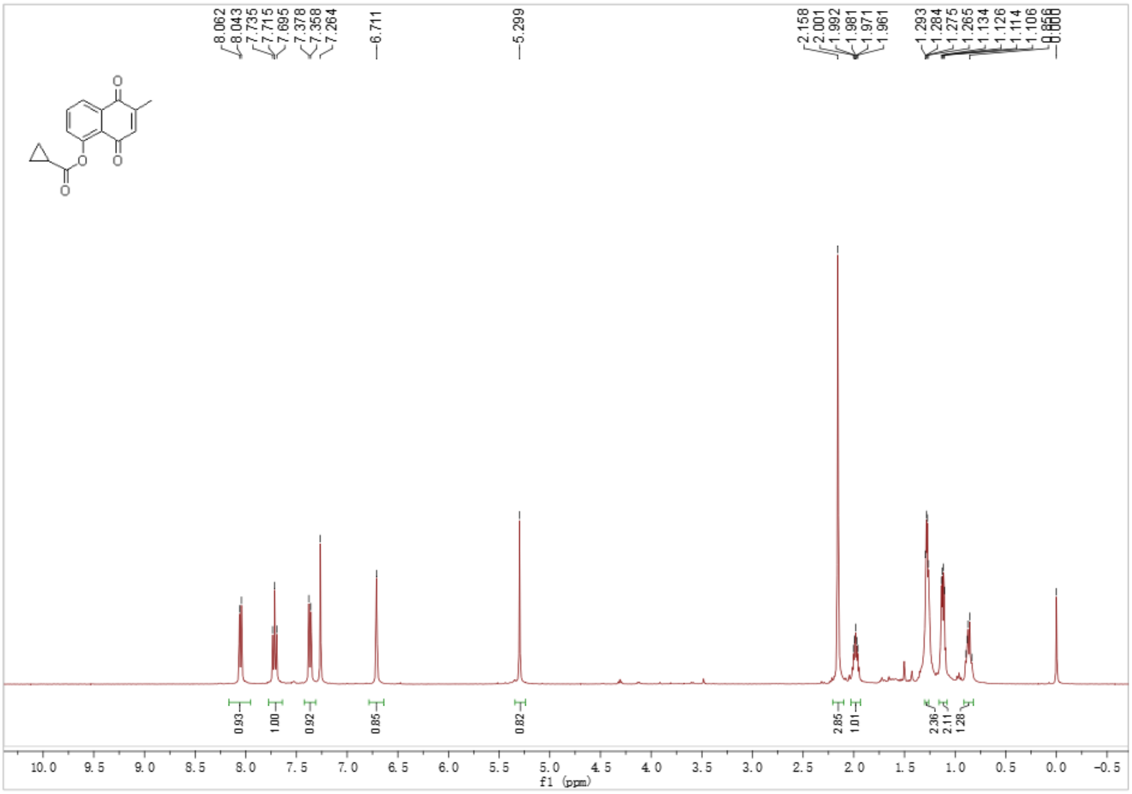

Figure S7. ^{1}H NMR spectrum of **I-1d**

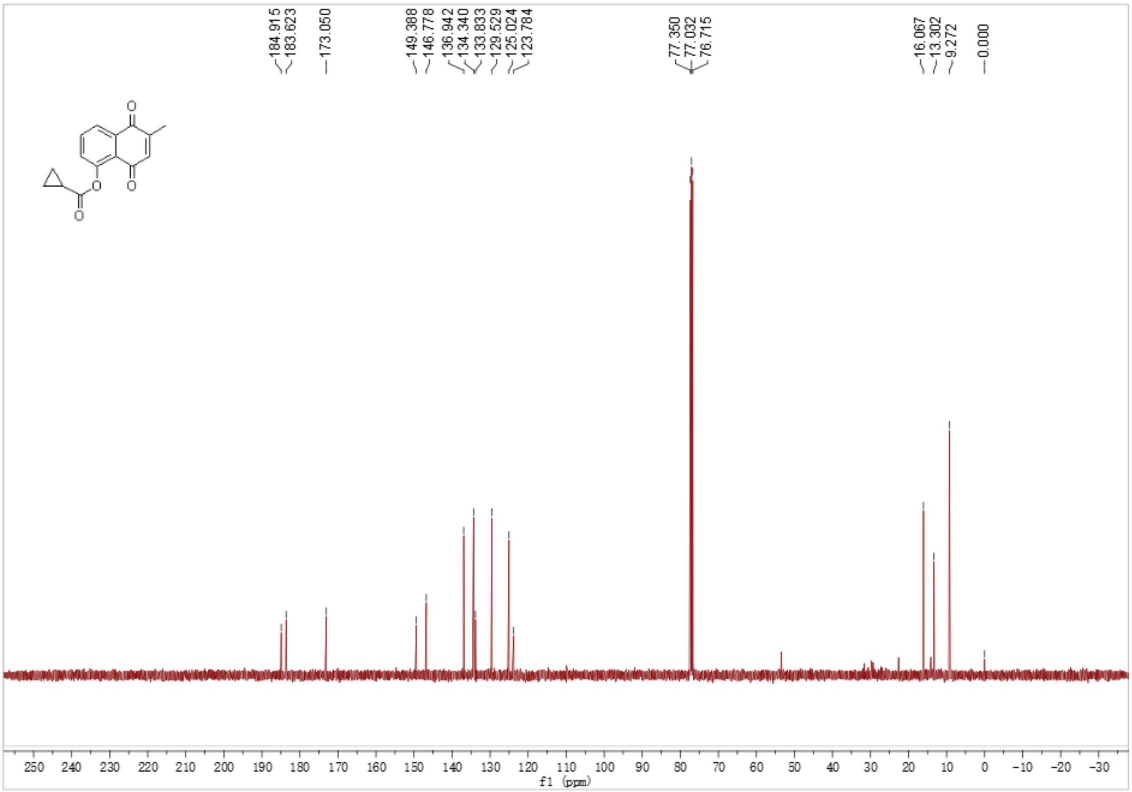

Figure S8. ^{13}C NMR spectrum of **I-1d**

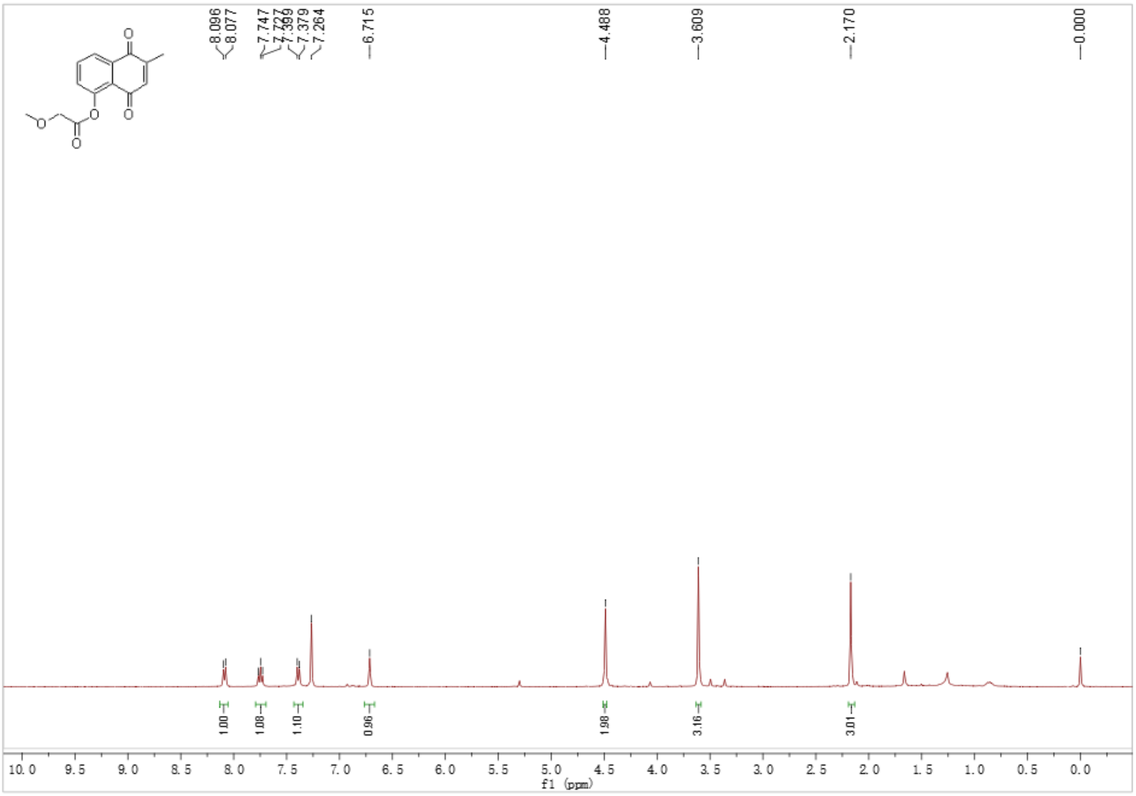

Figure S9. ^{1}H NMR spectrum of **I-1e**

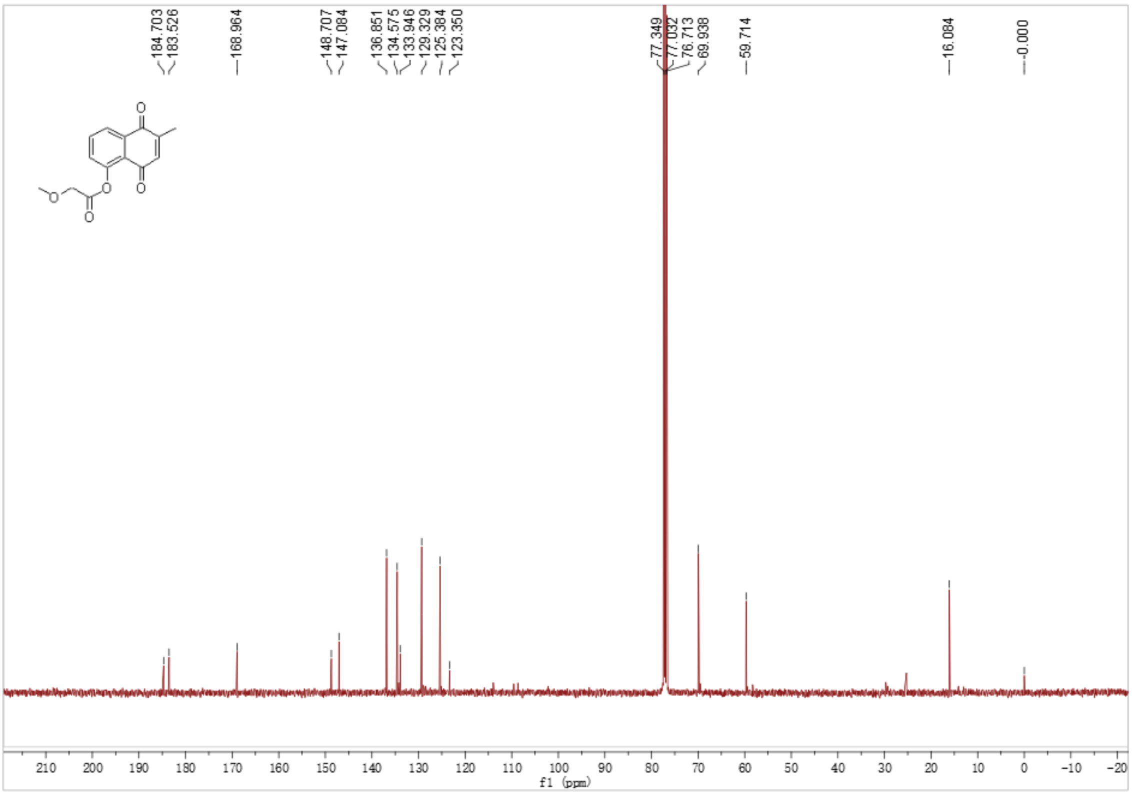

Figure S10. ^{13}C NMR spectrum of **I-1e**

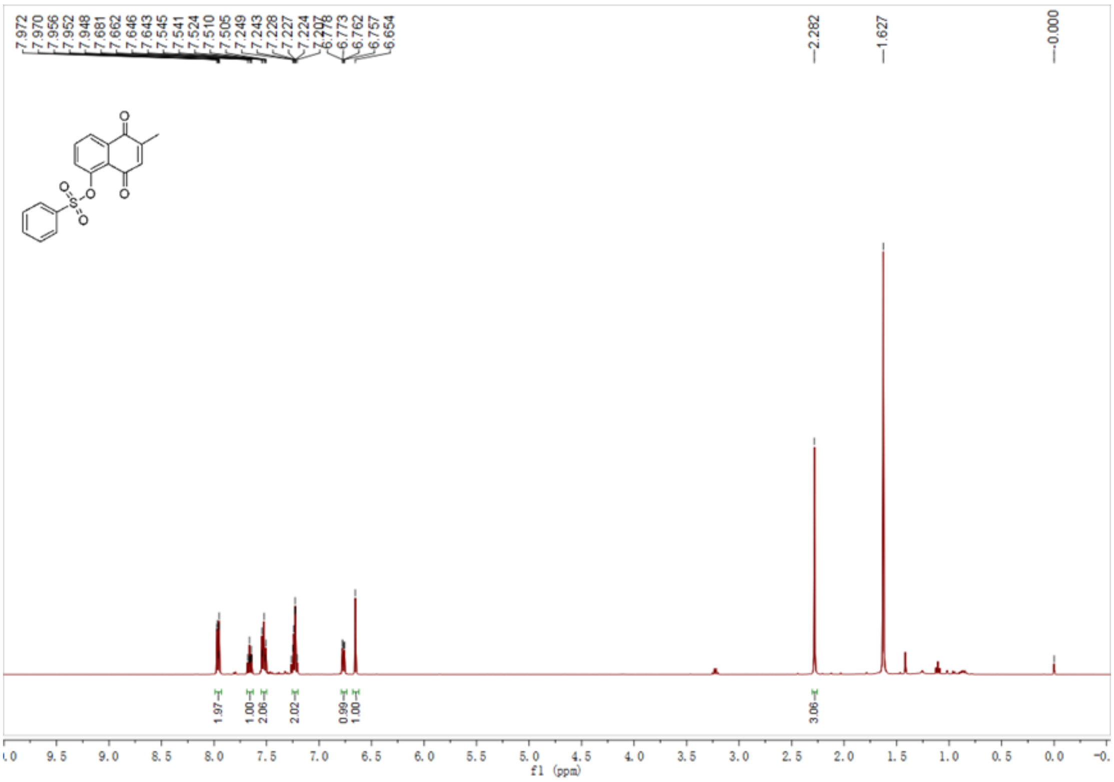

Figure S11. ^{1}H NMR spectrum of **I-1f**

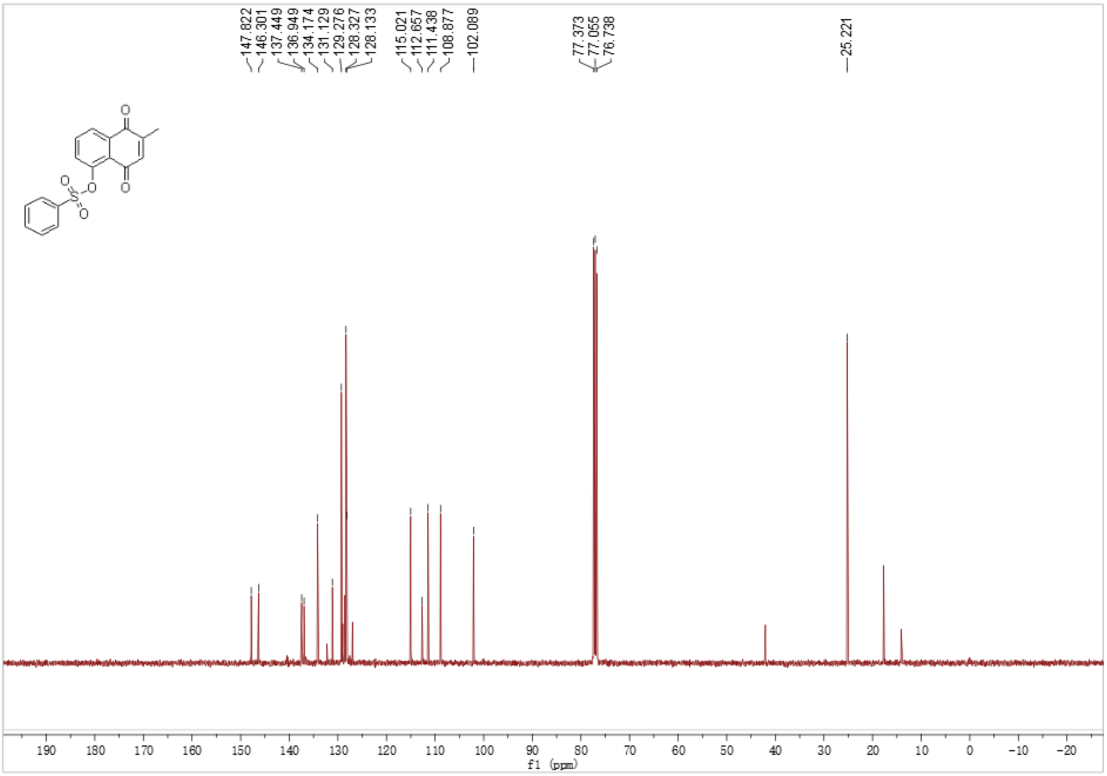

Figure S12. ^{13}C NMR spectrum of **I-1f**

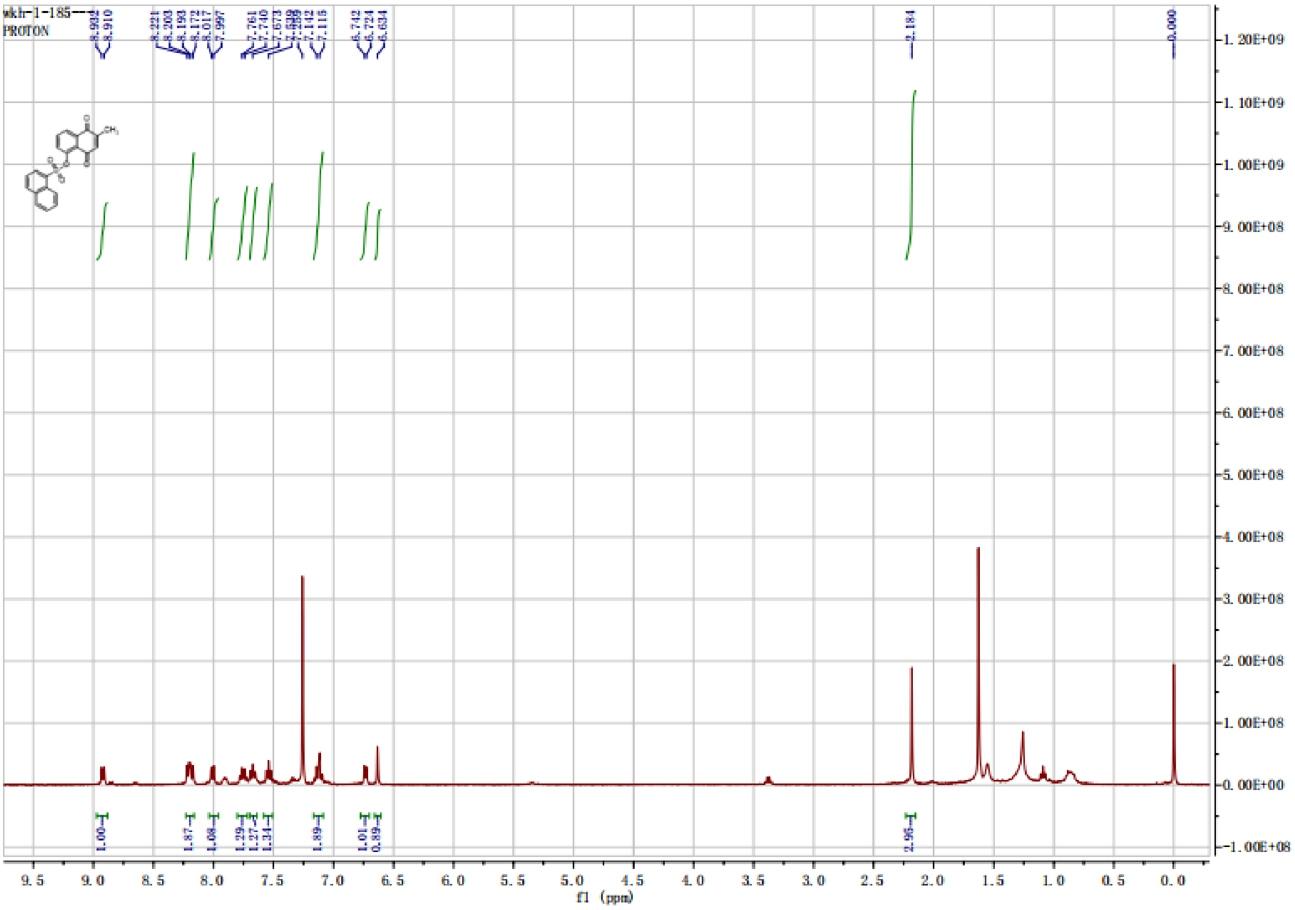

Figure S13. ^{1}H NMR spectrum of **I-1g**

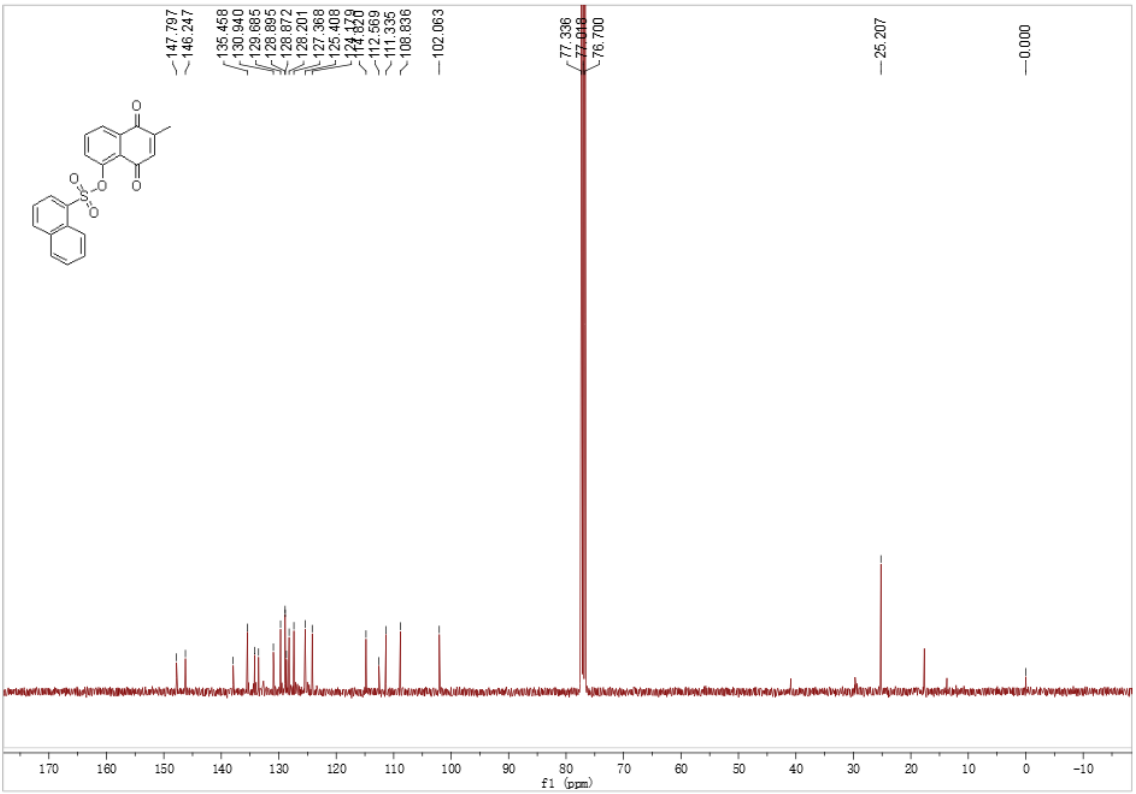

Figure S14. ^{13}C NMR spectrum of **I-1g**

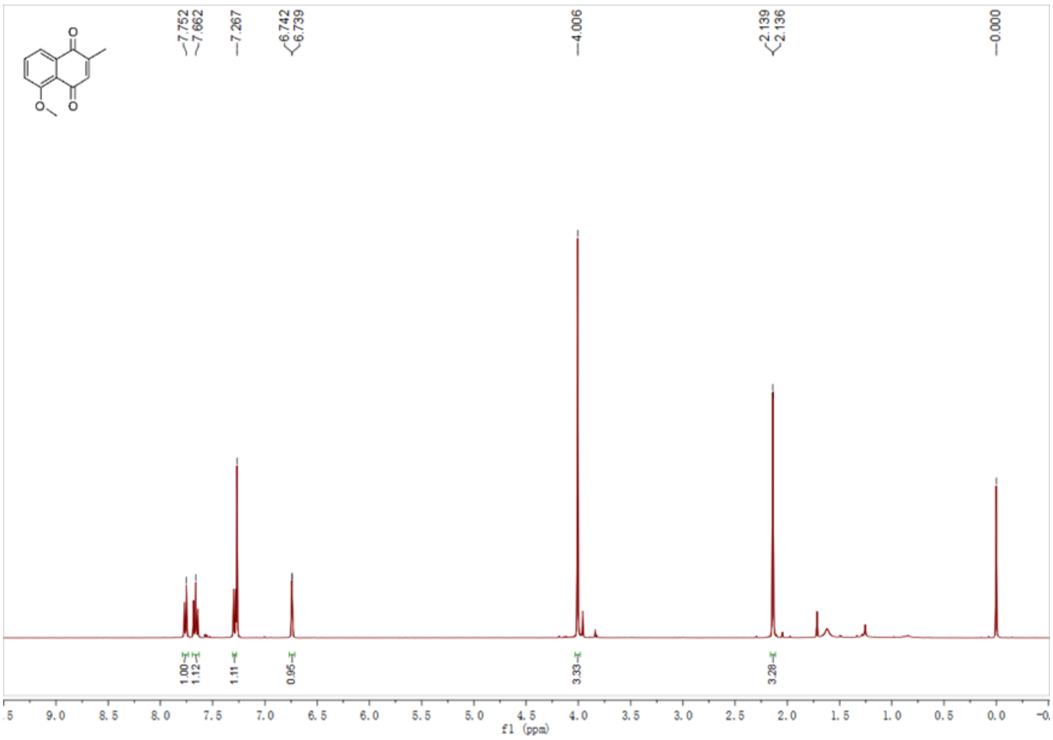

Figure S15. ^{1}H NMR spectrum of **I-1h**

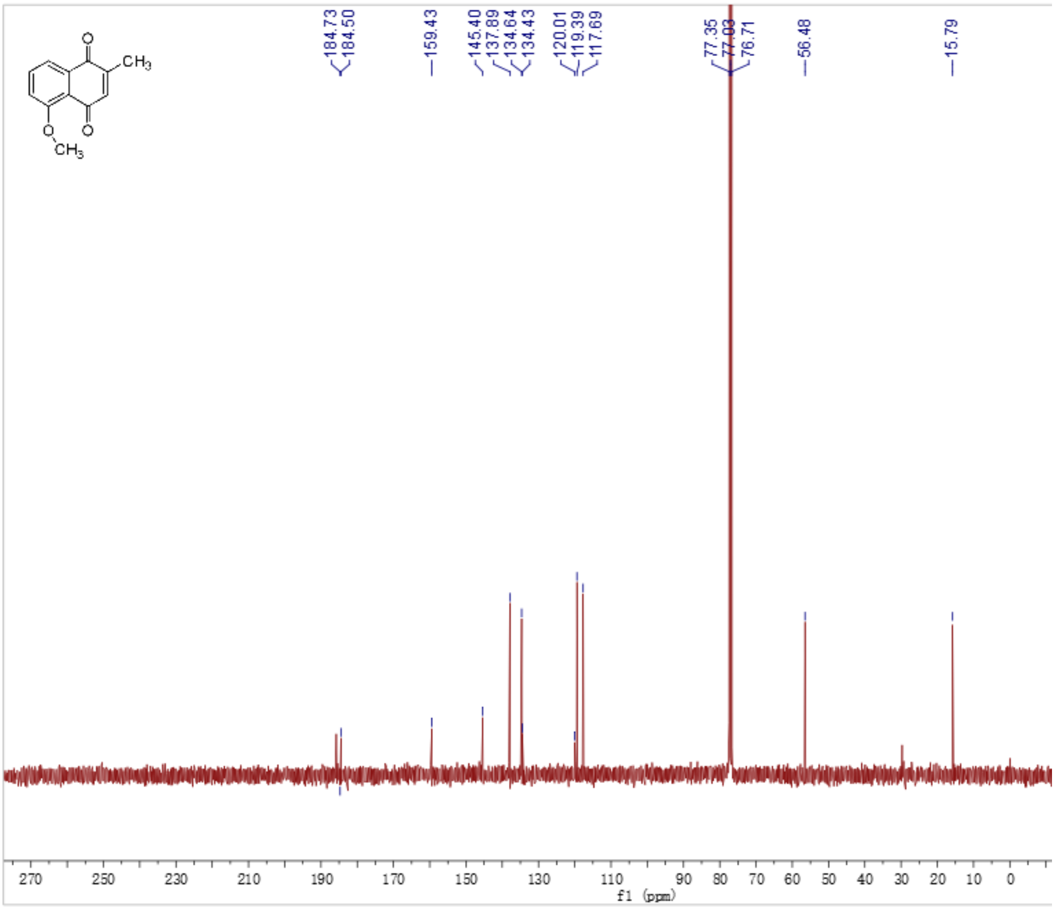

Figure S16. ^{13}C NMR spectrum of **I-1h**

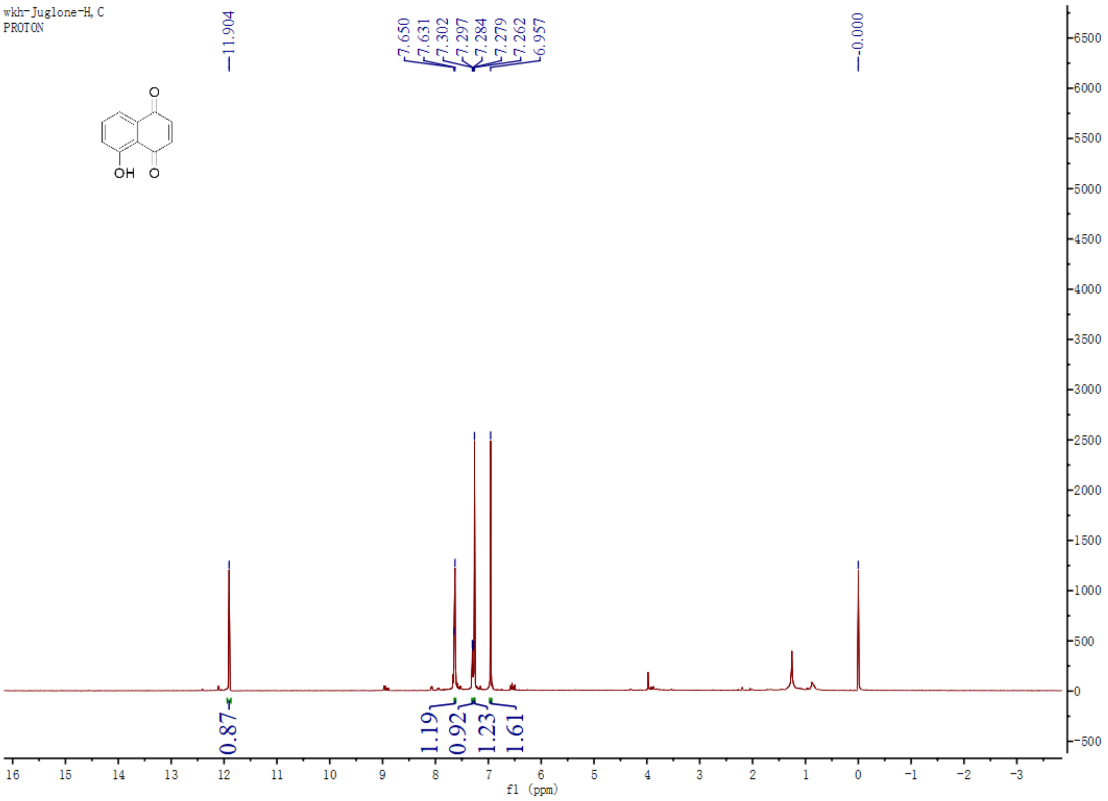

Figure S17. ^{1}H NMR spectrum of **II**

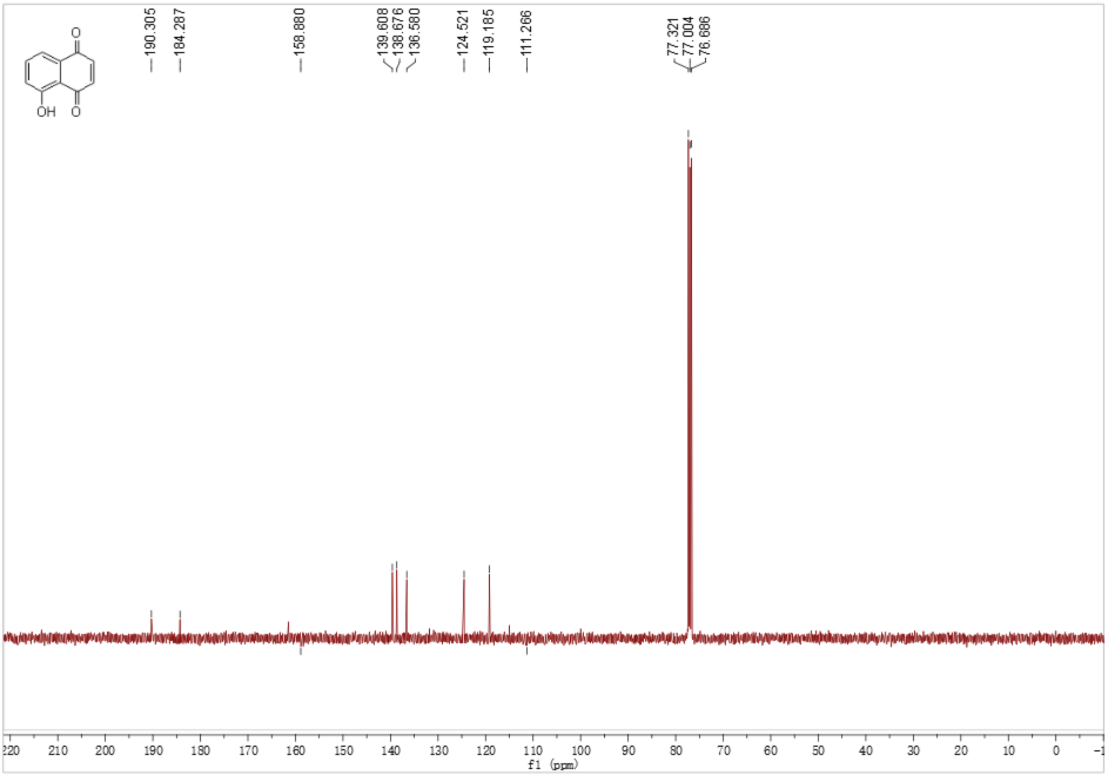

Figure S18. ^{13}C NMR spectrum of **II**

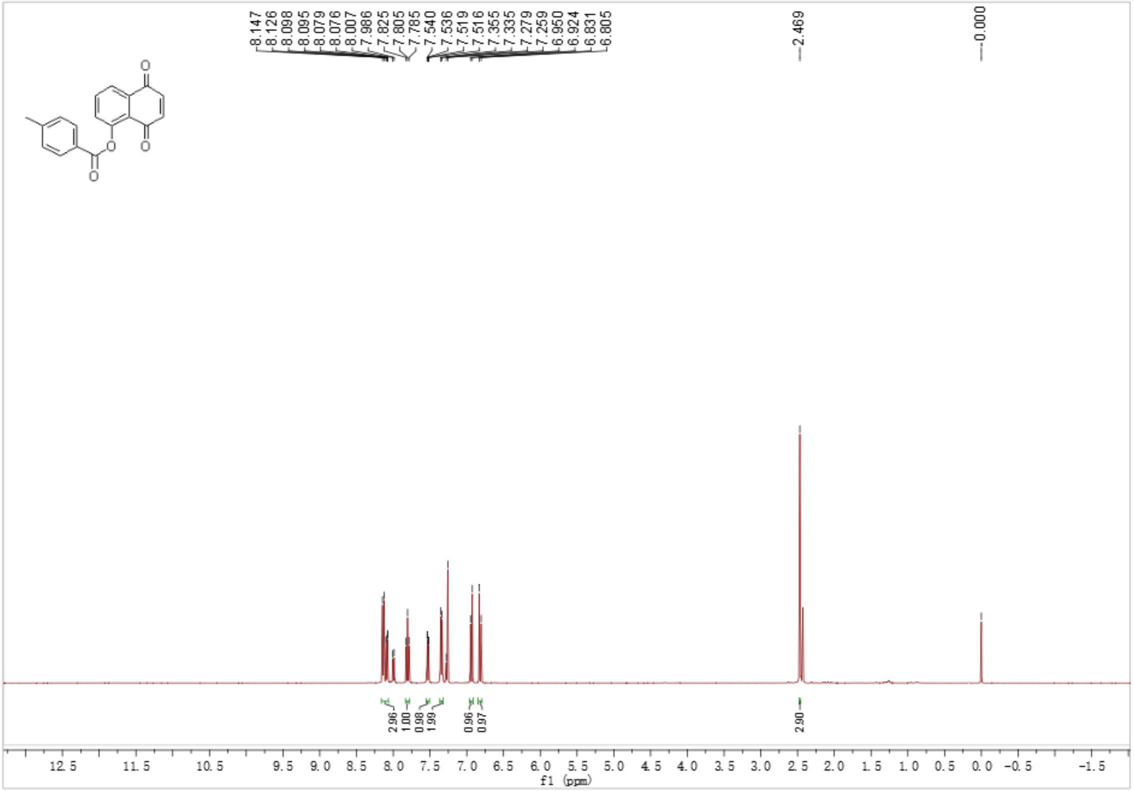

Figure S19. ^{1}H NMR spectrum of **II-1a**

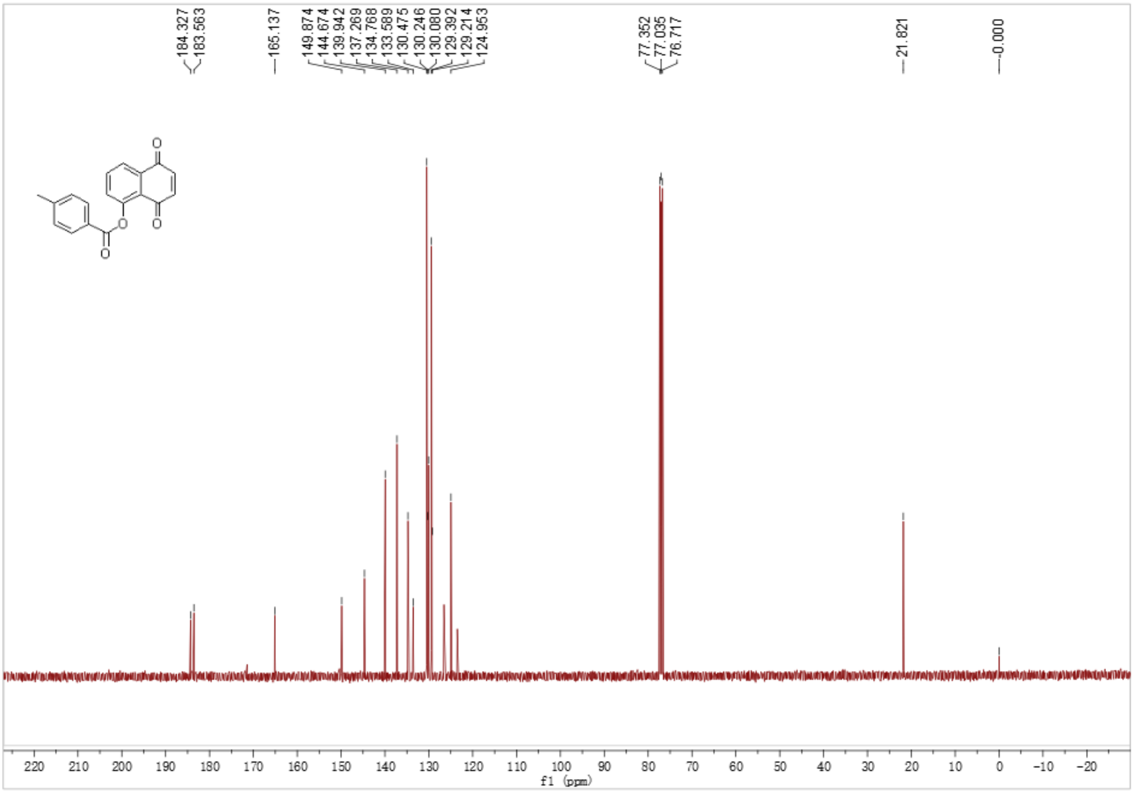

Figure S20. ^{13}C NMR spectrum of **II-1a**

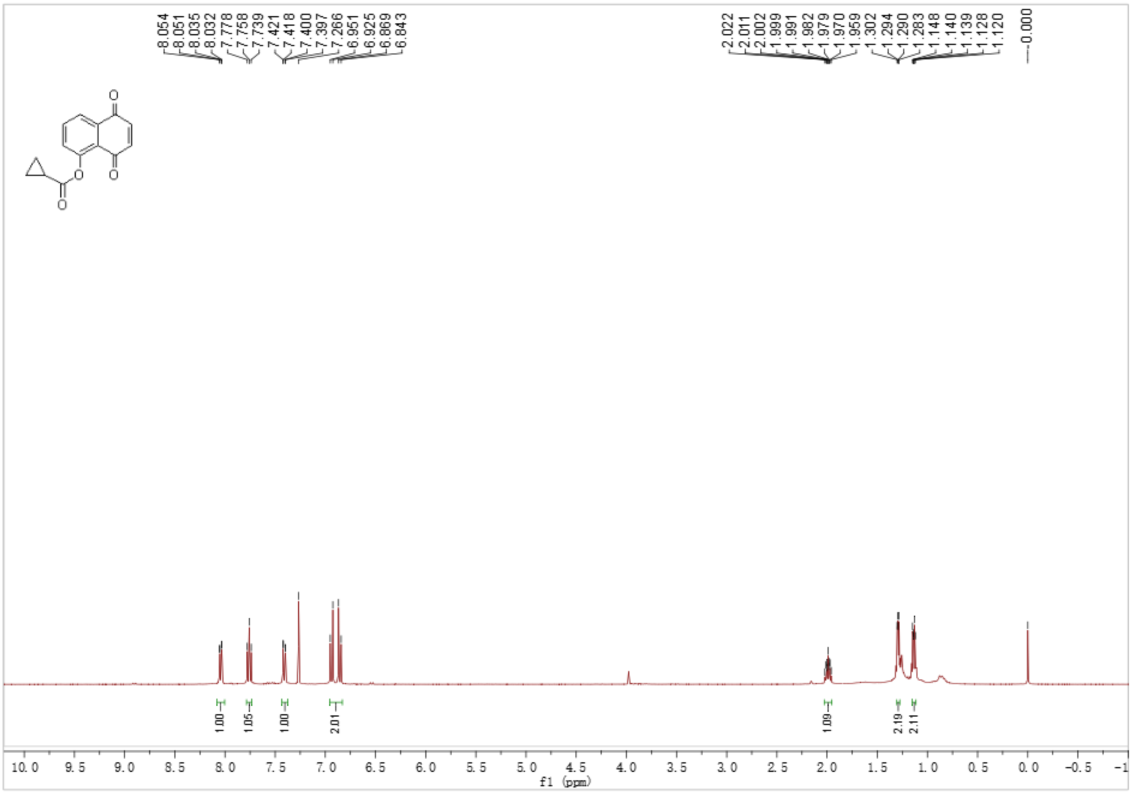

Figure S21. ^{1}H NMR spectrum of **II-1b**

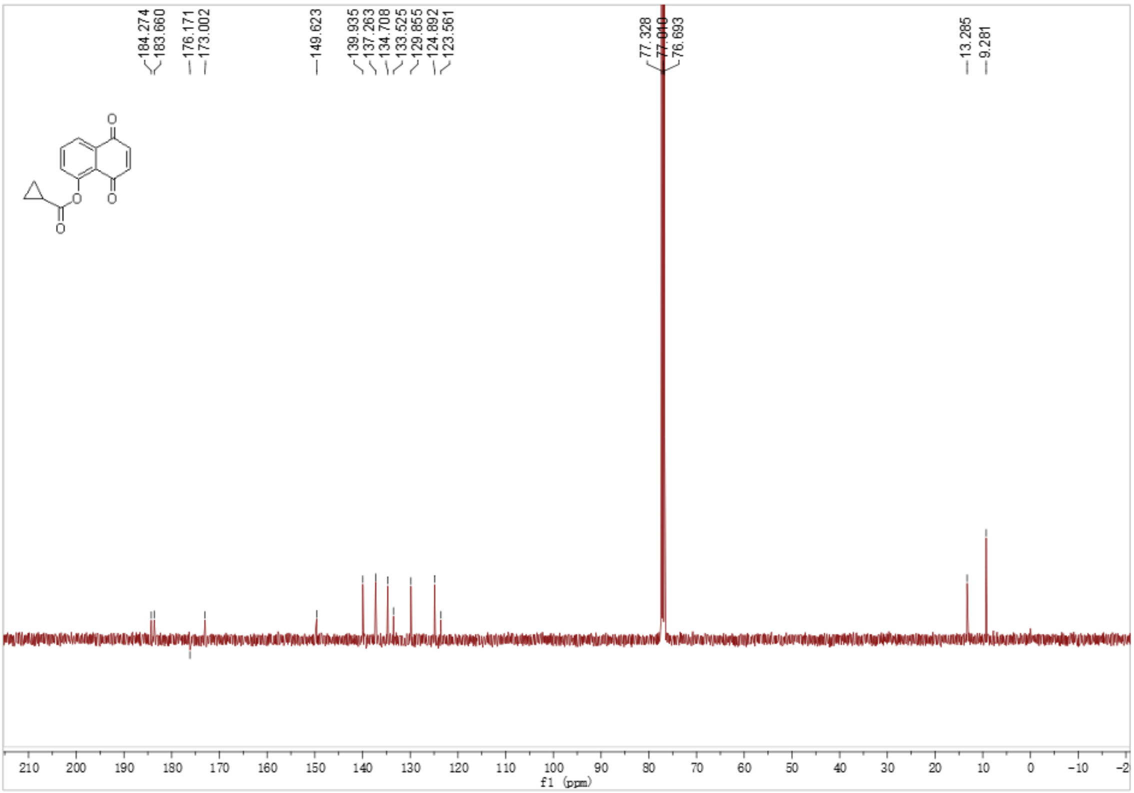

Figure S22. ^{13}C NMR spectrum of **II-1b**

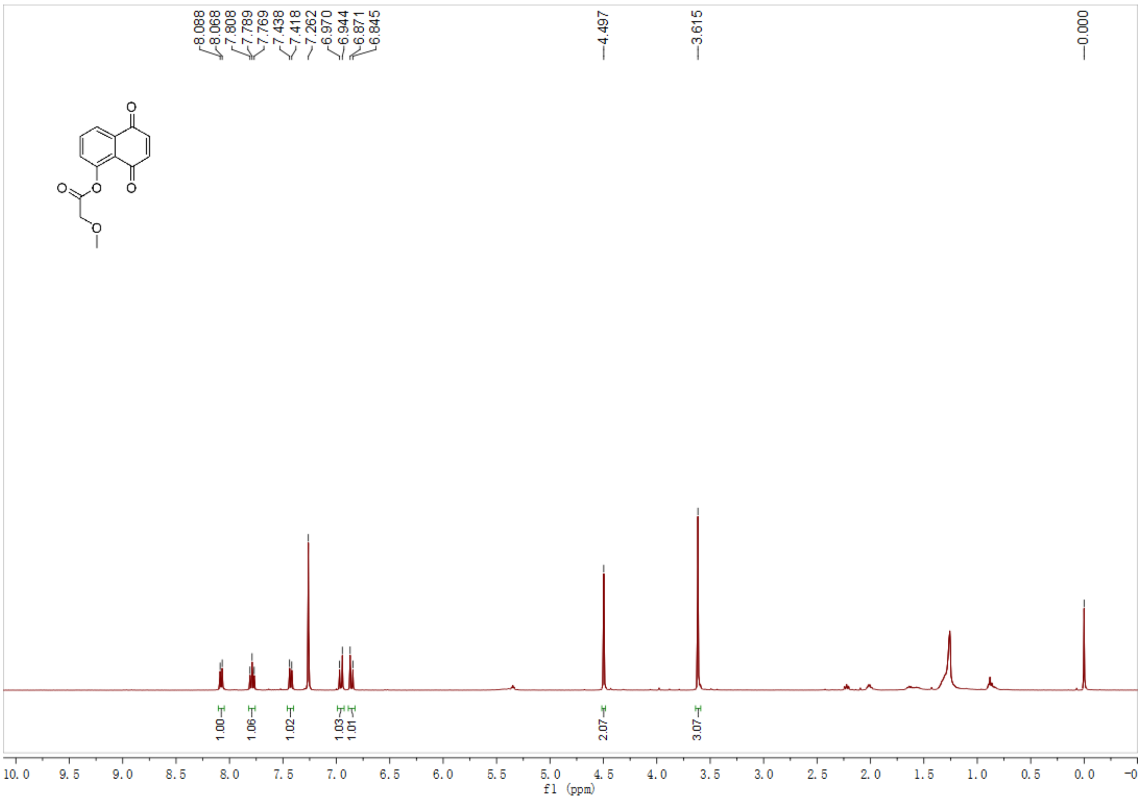

Figure S23. ^{1}H NMR spectrum of **II-1c**

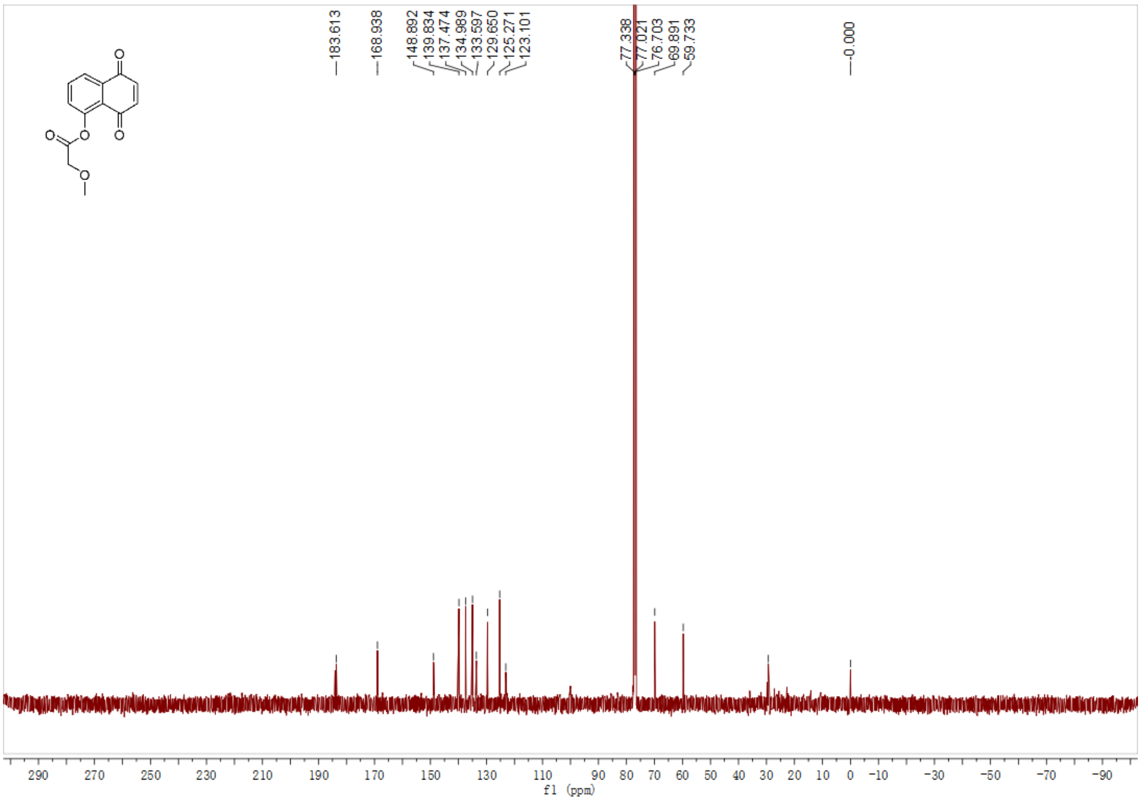

Figure S24. ^{13}C NMR spectrum of **II-1c**

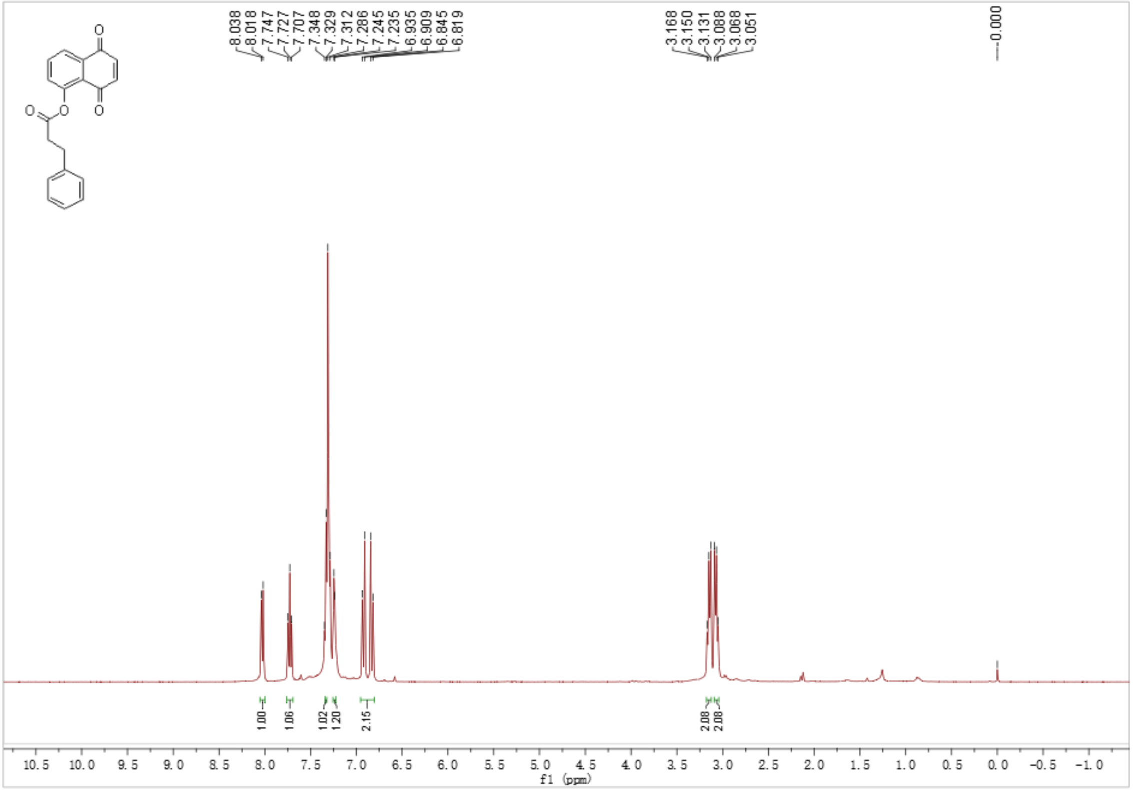

Figure S25. ^{1}H NMR spectrum of **II-1d**

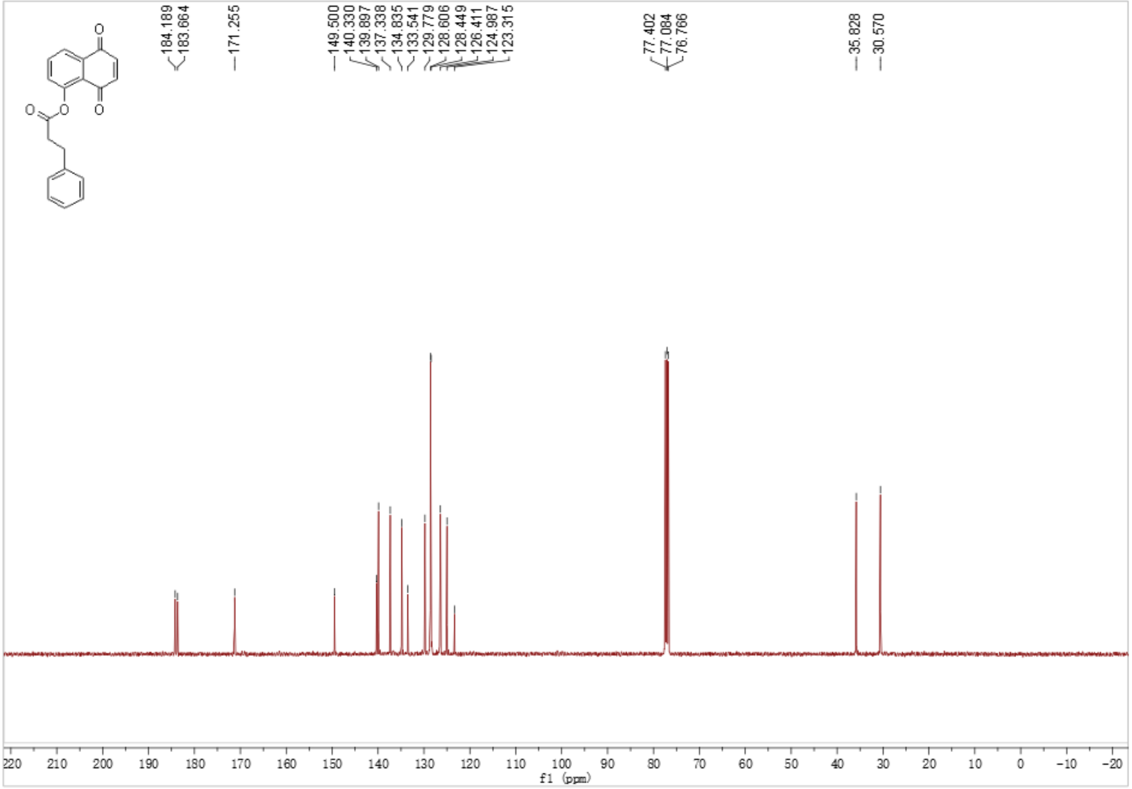

Figure S26. ^{13}C NMR spectrum of **II-1d**

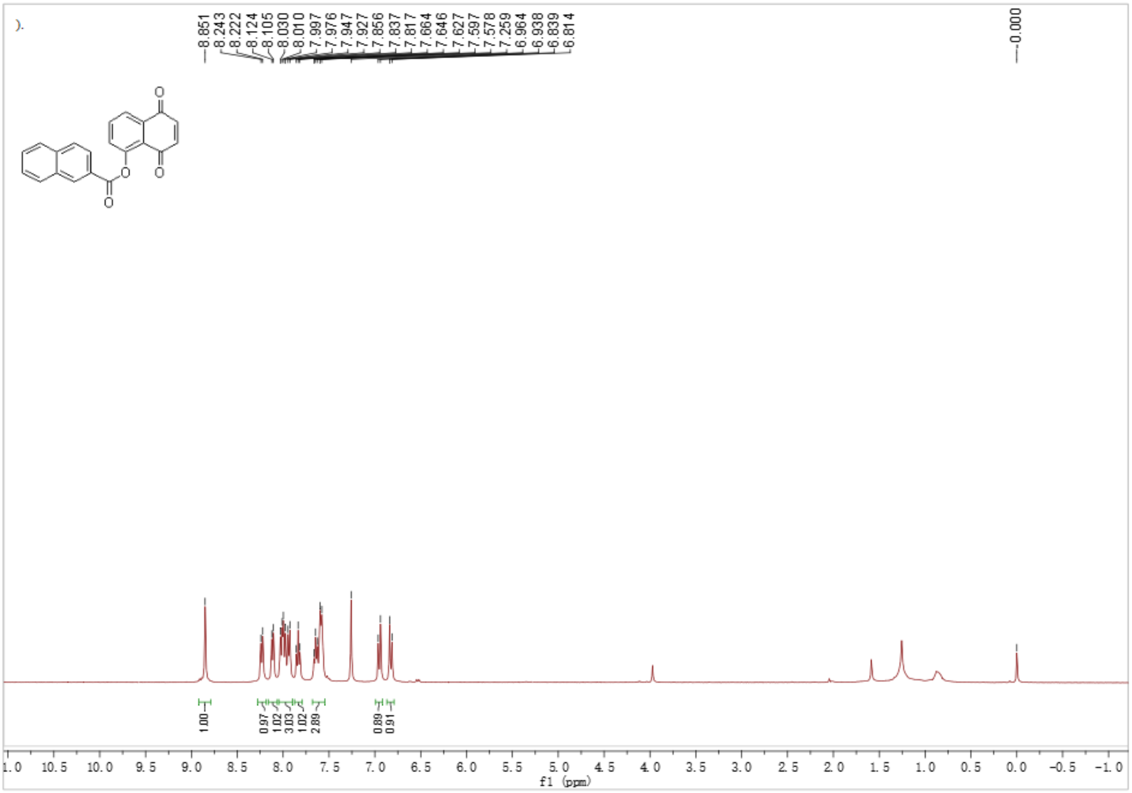

Figure S27. ^{1}H NMR spectrum of **II-1e**

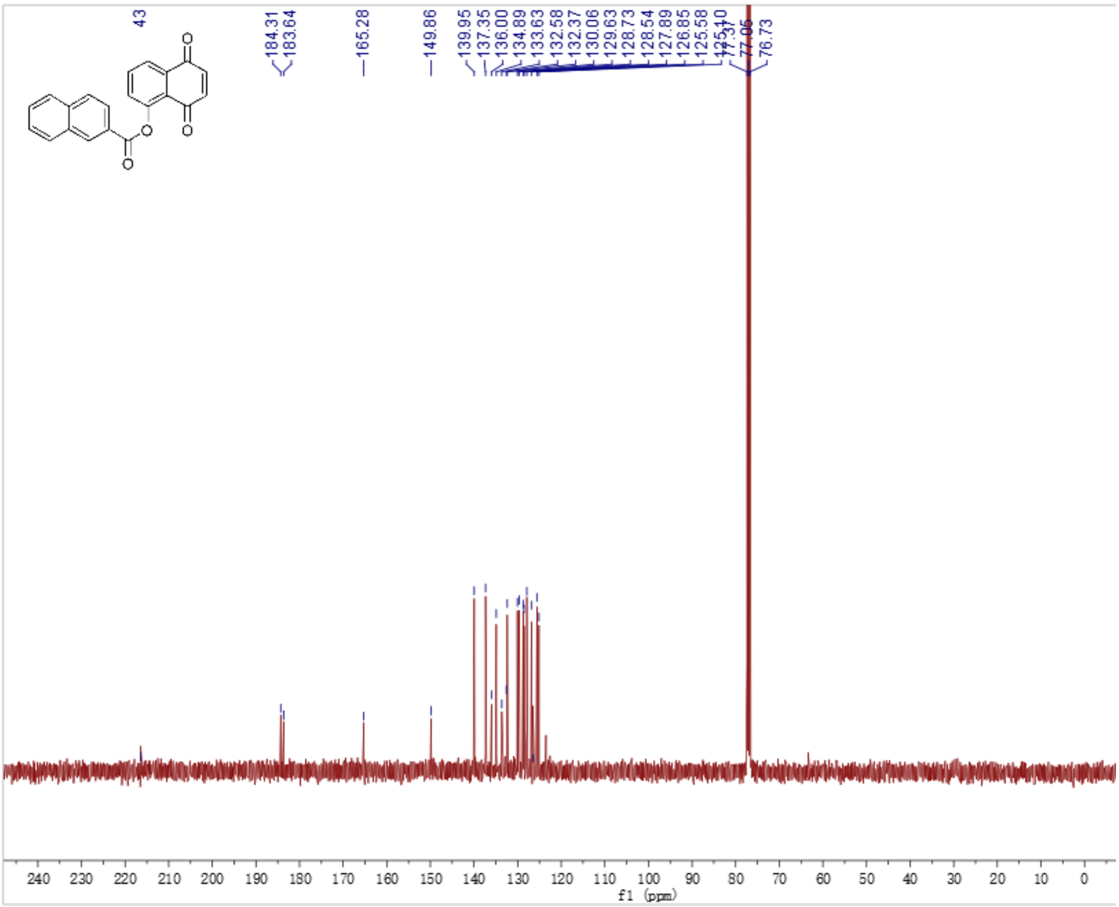

Figure S28. ^{13}C NMR spectrum of **II-1e**

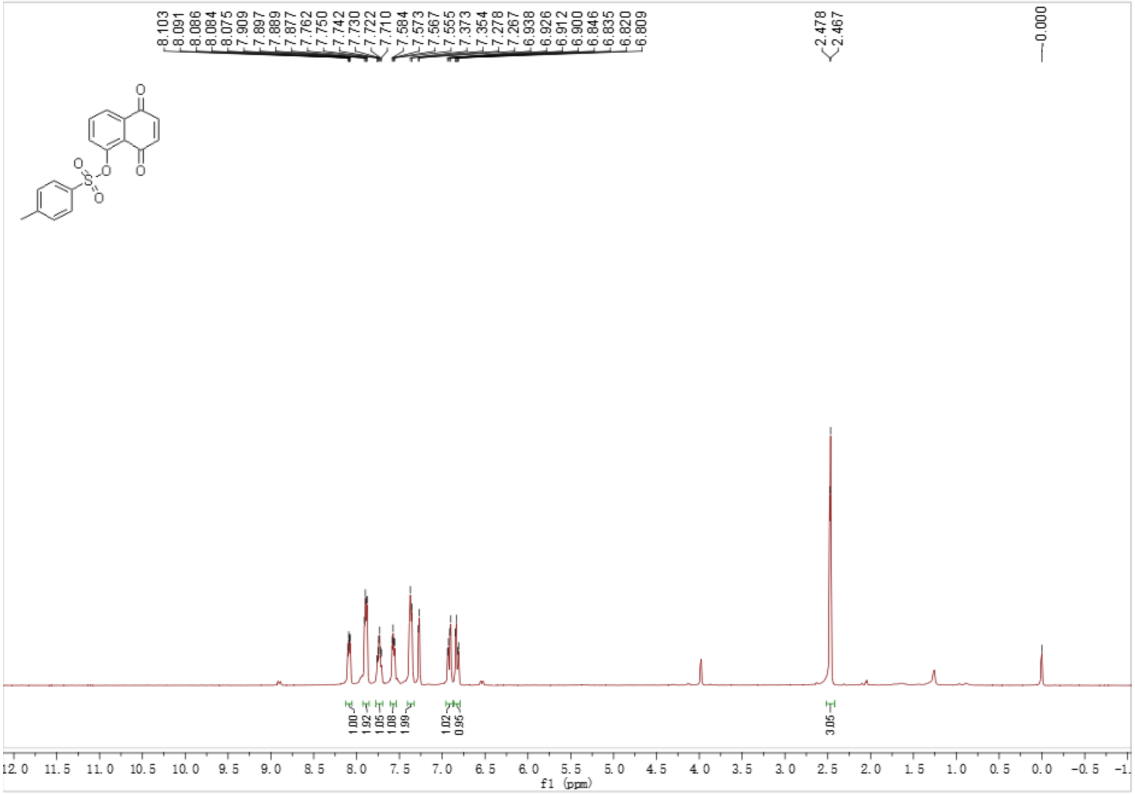

Figure S29. ^{1}H NMR spectrum of **II-1f**

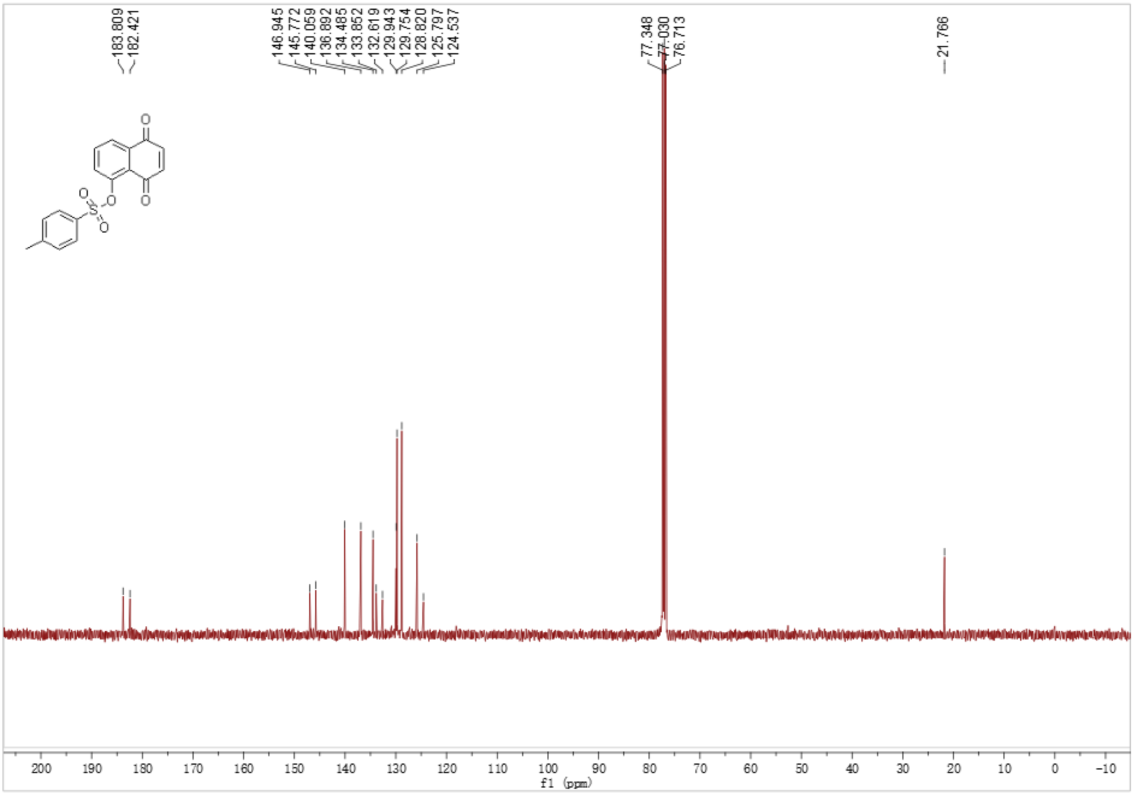

Figure S30. ^{13}C NMR spectrum of **II-1f**

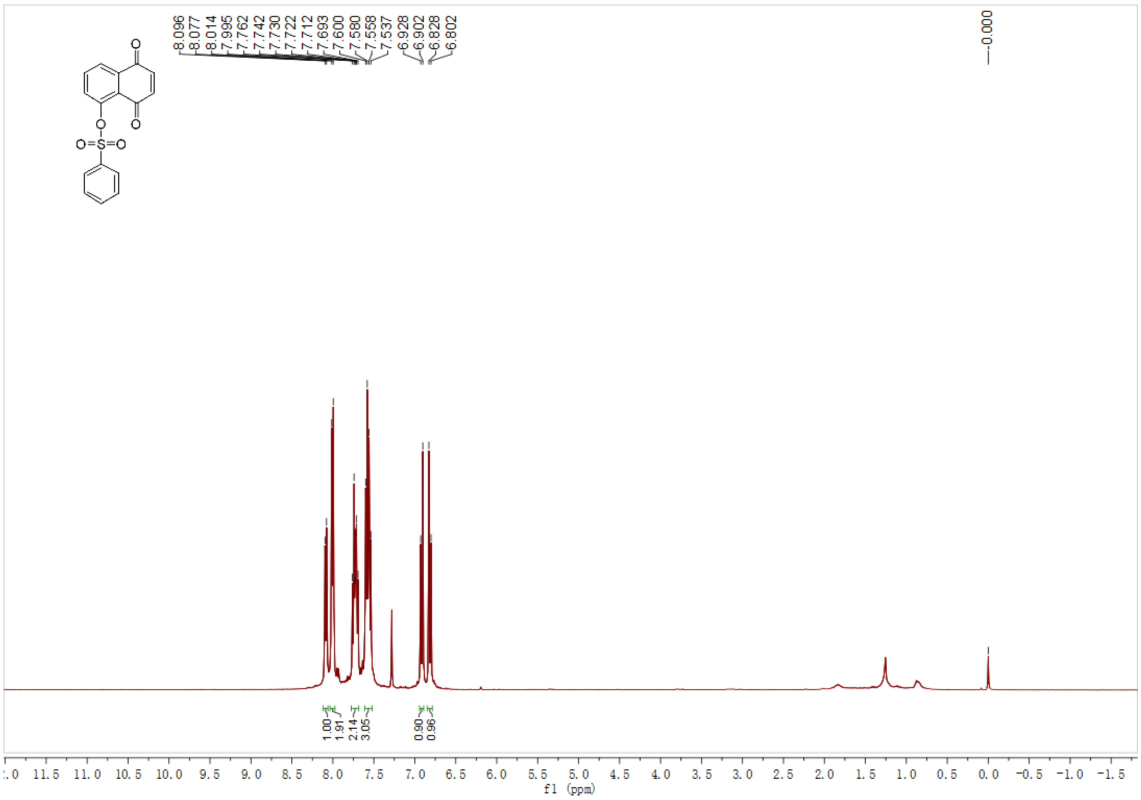

Figure S31. ^{1}H NMR spectrum of **II-1g**

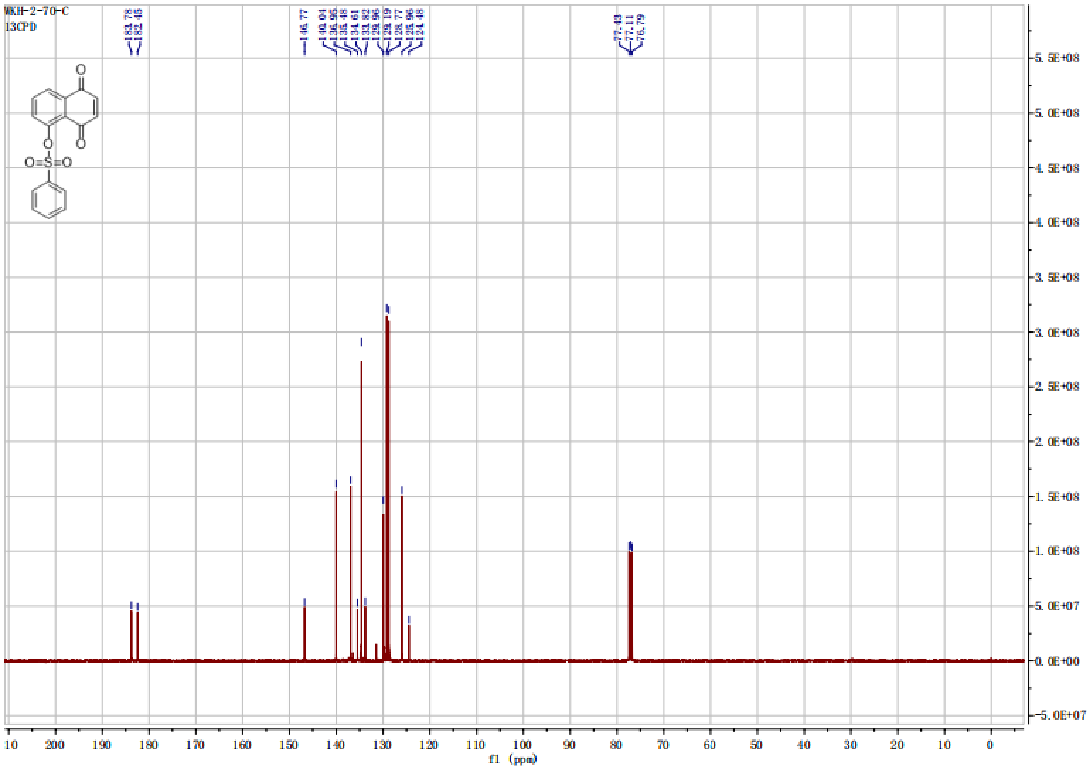

Figure S32. ^{13}C NMR spectrum of **II-1g**

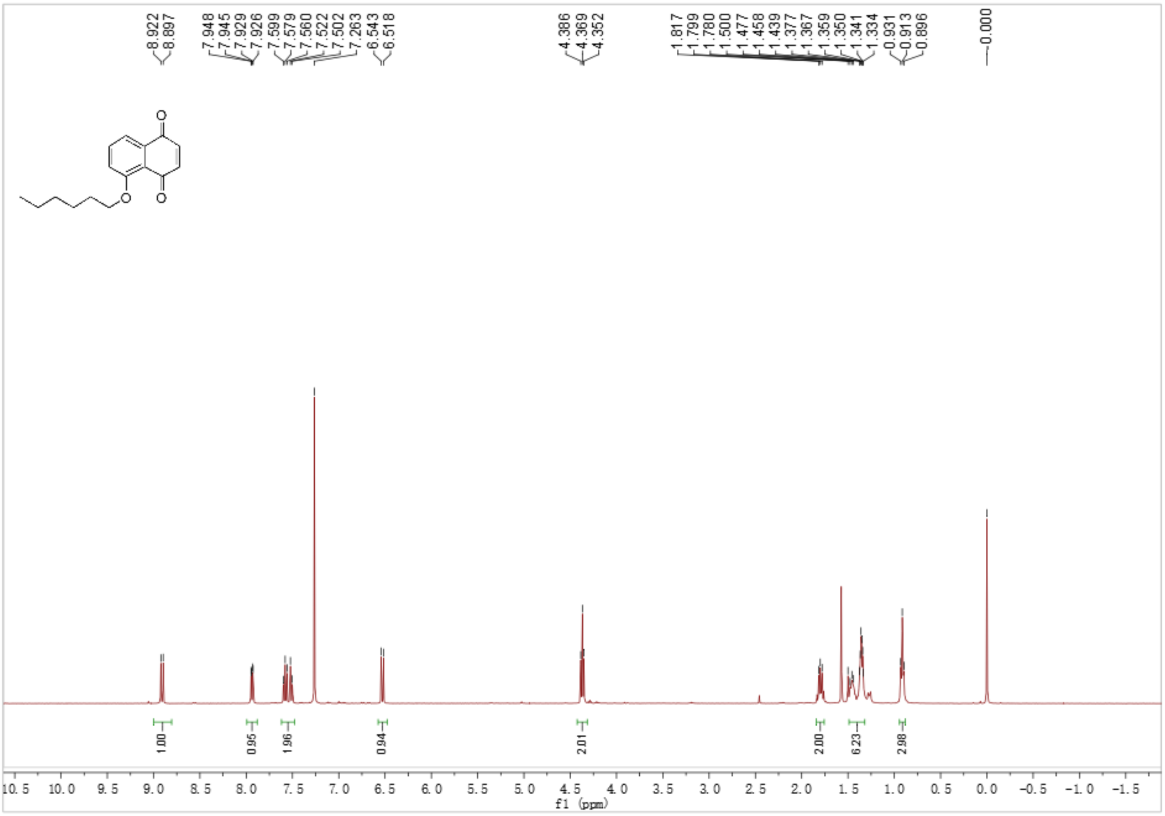

Figure S33. ^{1}H NMR spectrum of **II-1h**

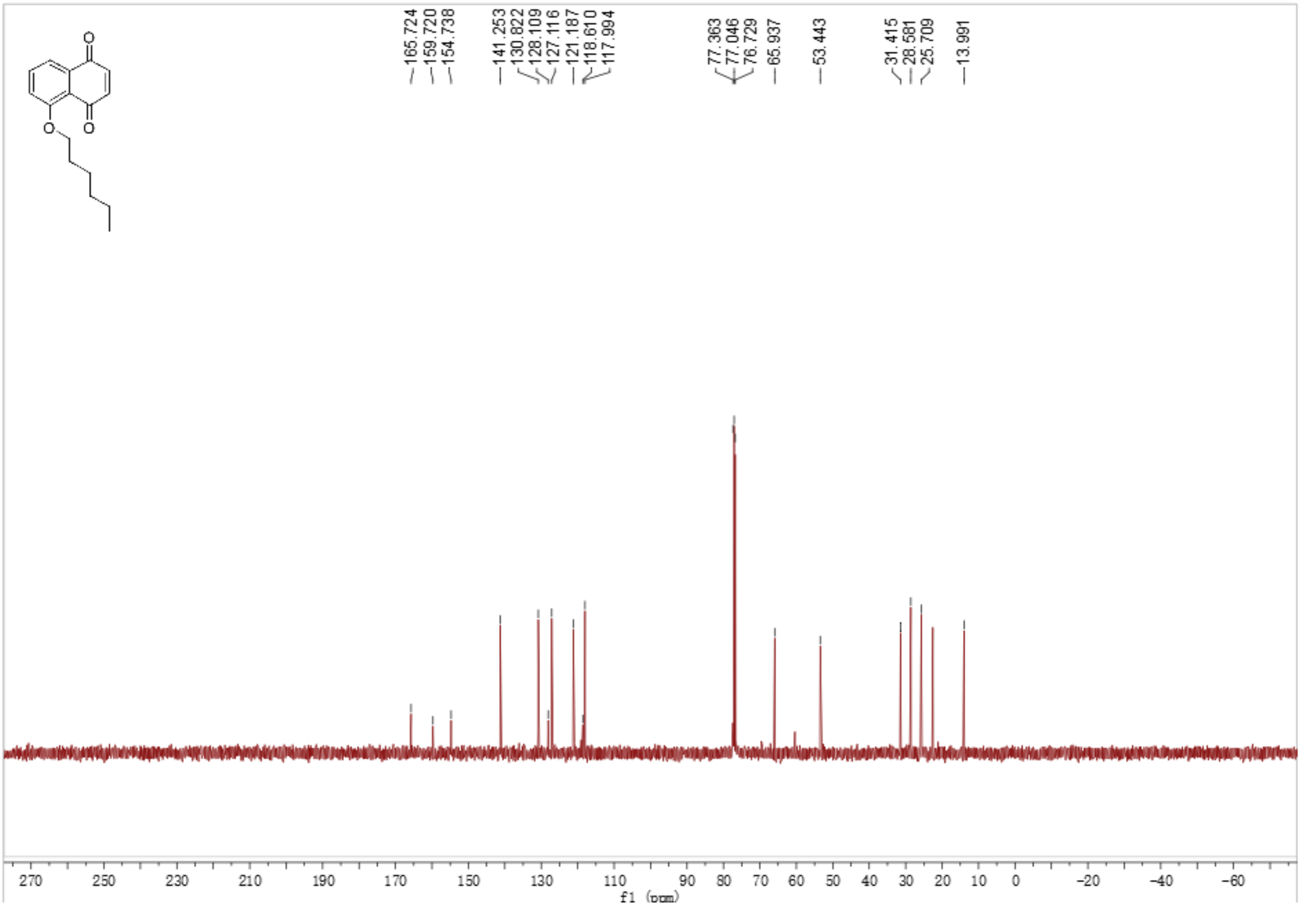

Figure S34. ^{13}C NMR spectrum of **II-1h**

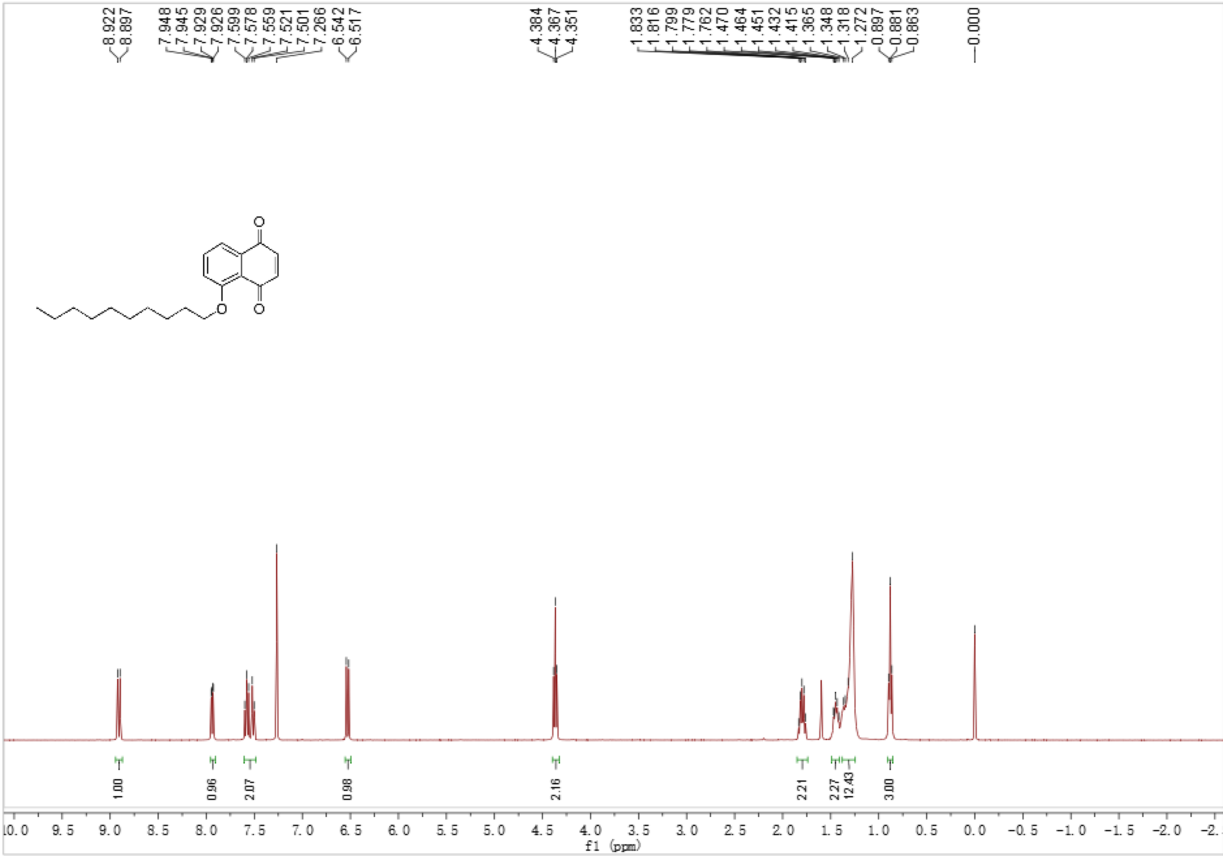

Figure S35. ^{1}H NMR spectrum of **II-1i**

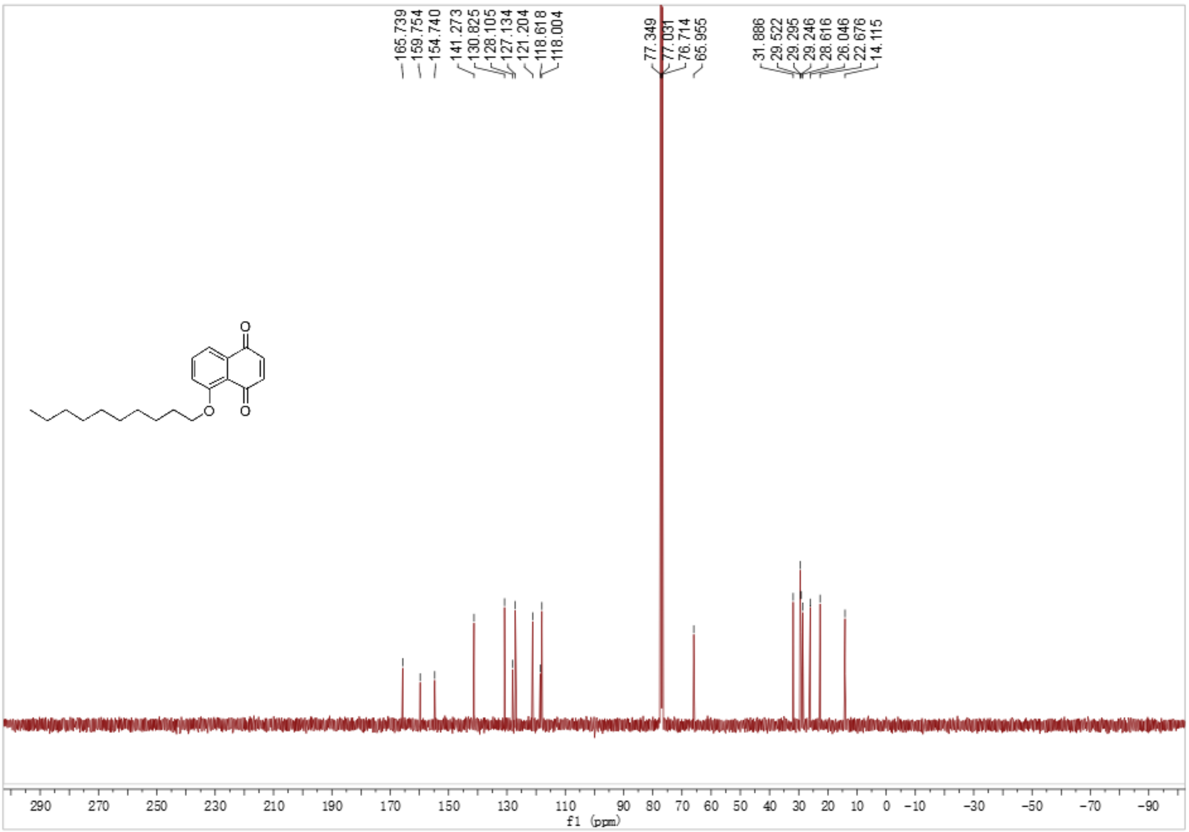

Figure S36. ^{13}C NMR spectrum of **II-1i**

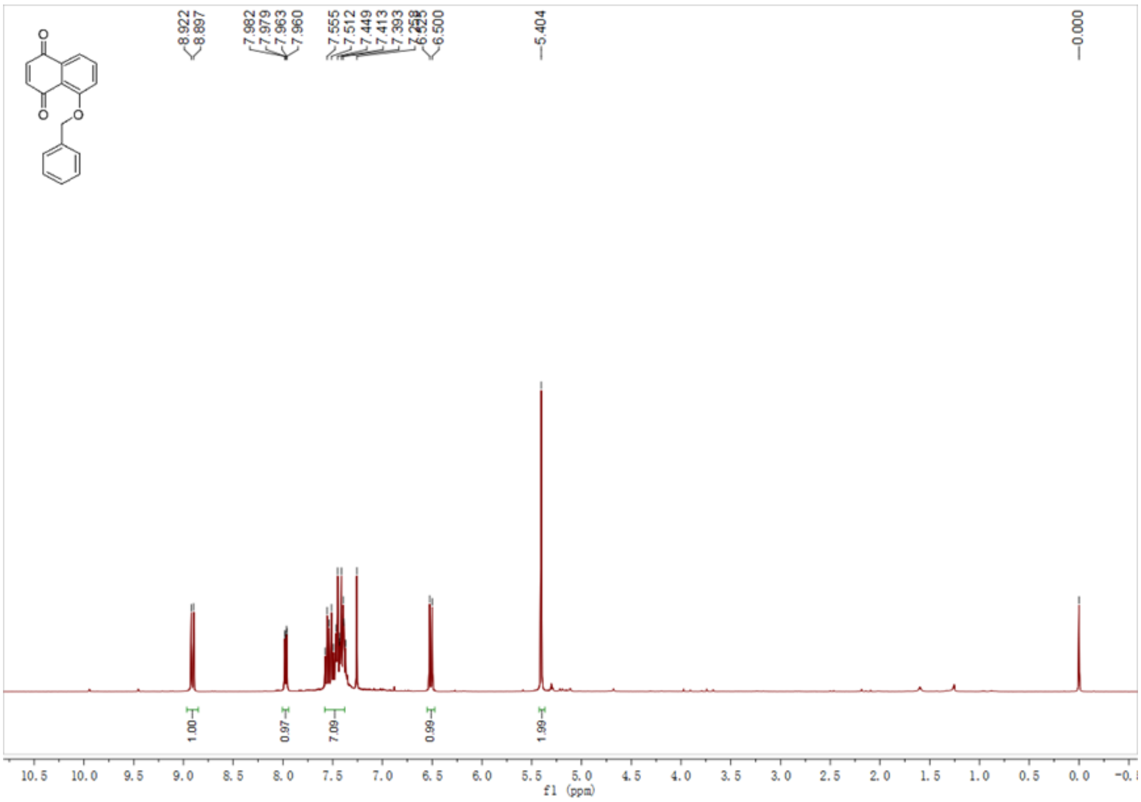

Figure S37. ^{1}H NMR spectrum of **II-1j**

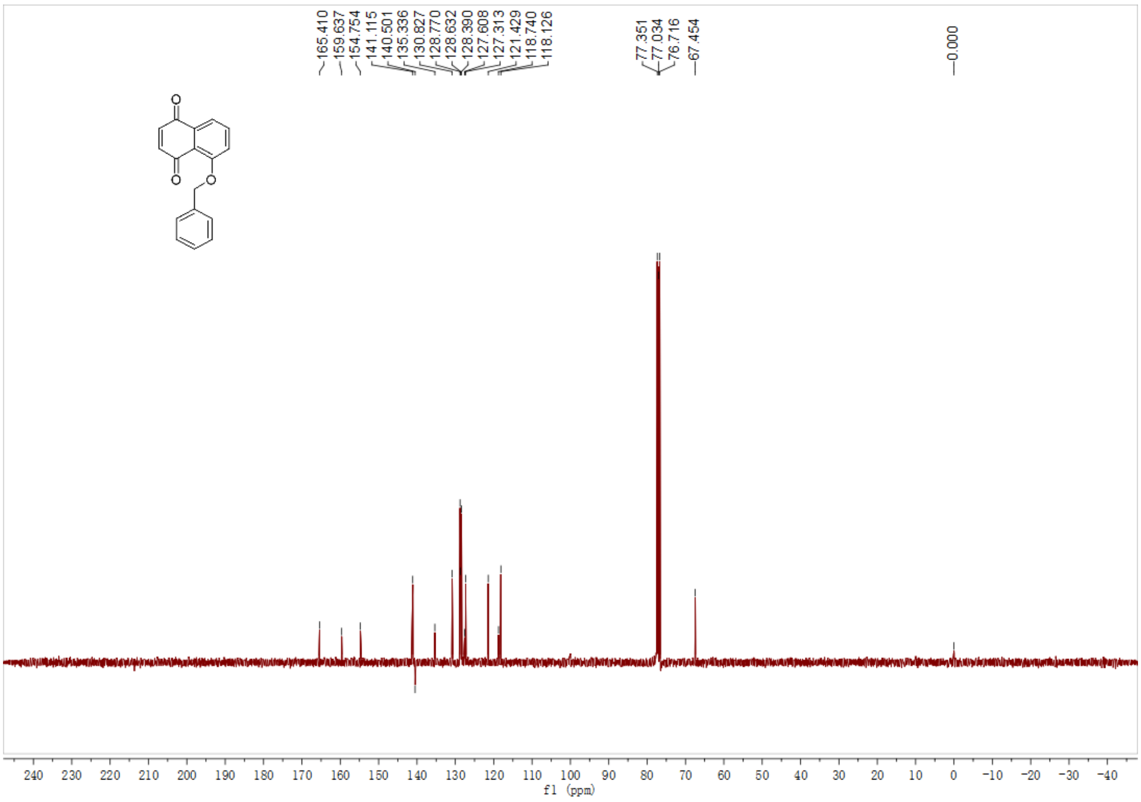

Figure S38. ^{13}C NMR spectrum of **II-1j**

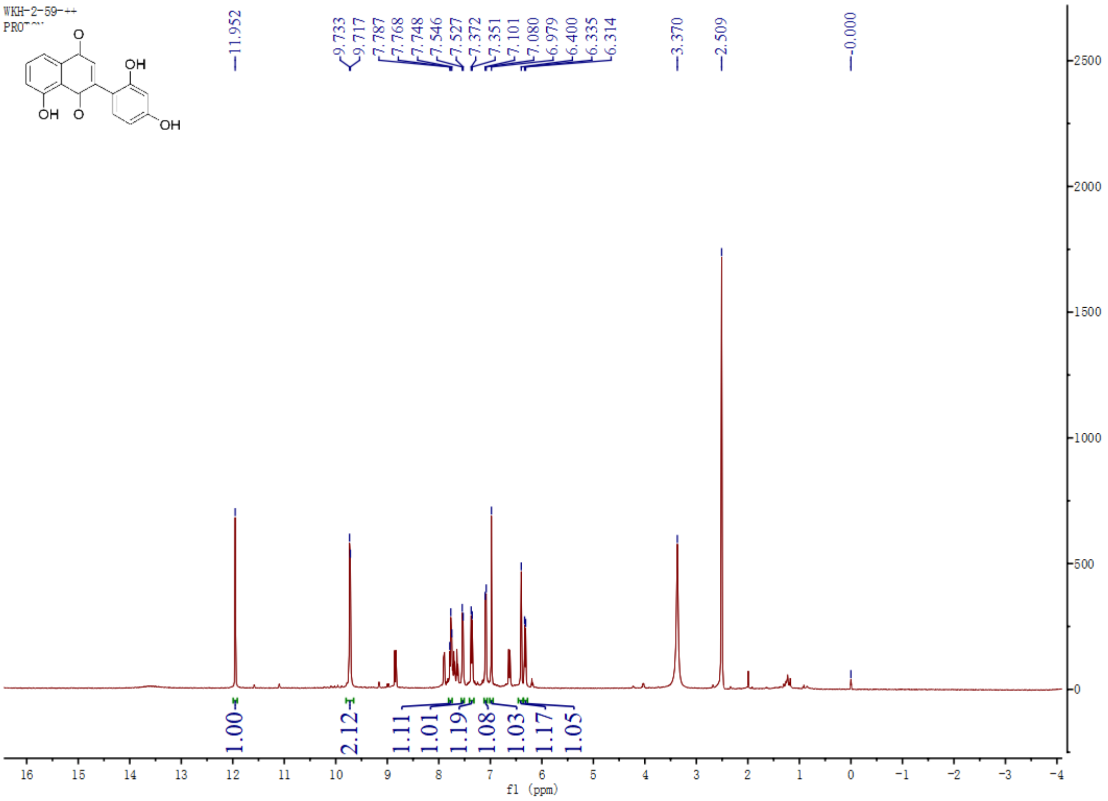

Figure S39. ^{1}H NMR spectrum of **II-1k**

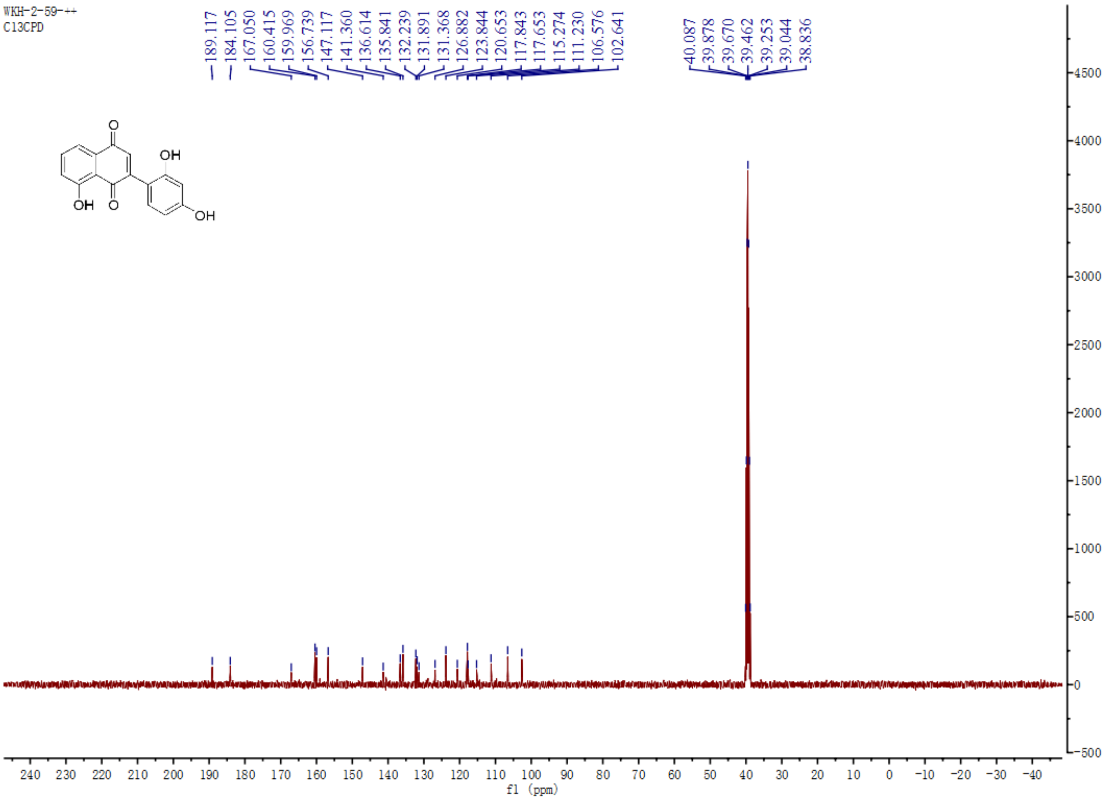

Figure S40. ^{13}C NMR spectrum of **II-1k**

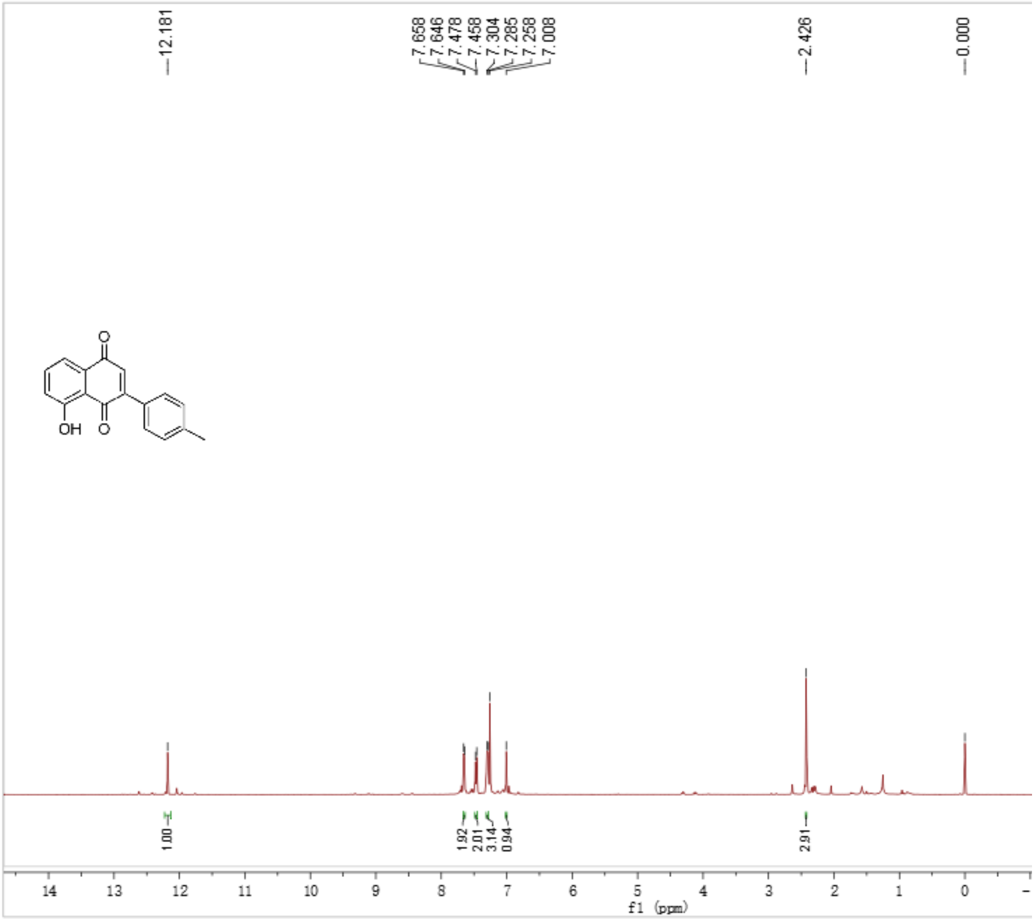

Figure S41. ^{1}H NMR spectrum of **II-1l**

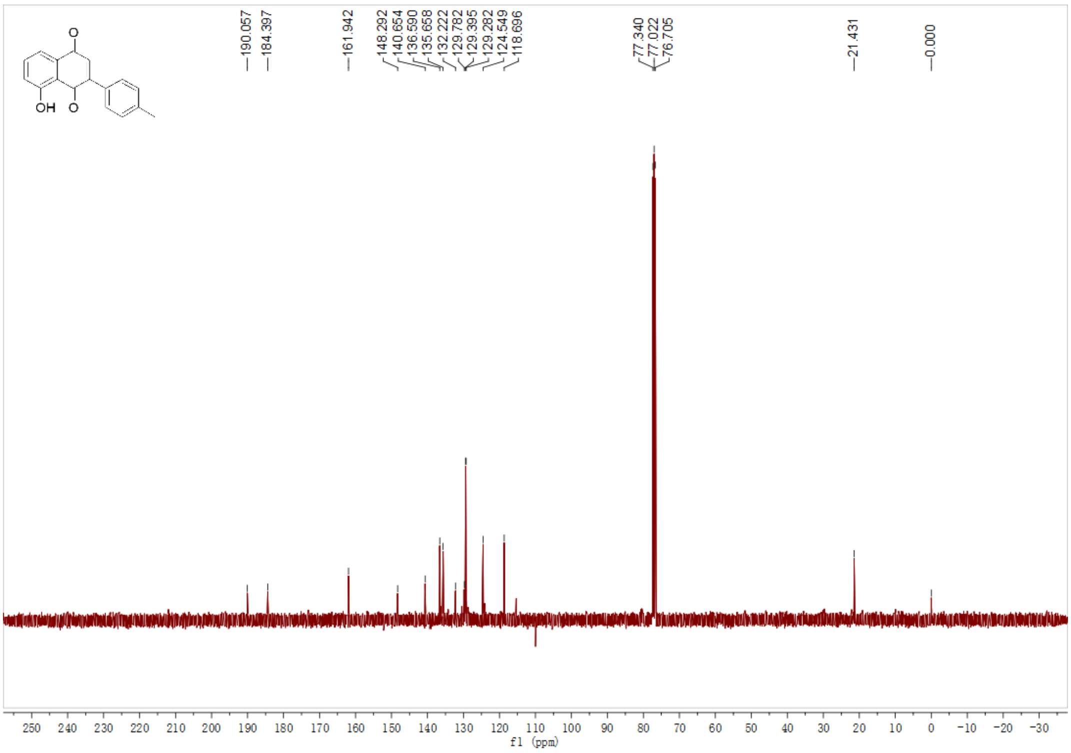

Figure S42. ^{13}C NMR spectrum of **II-1l**

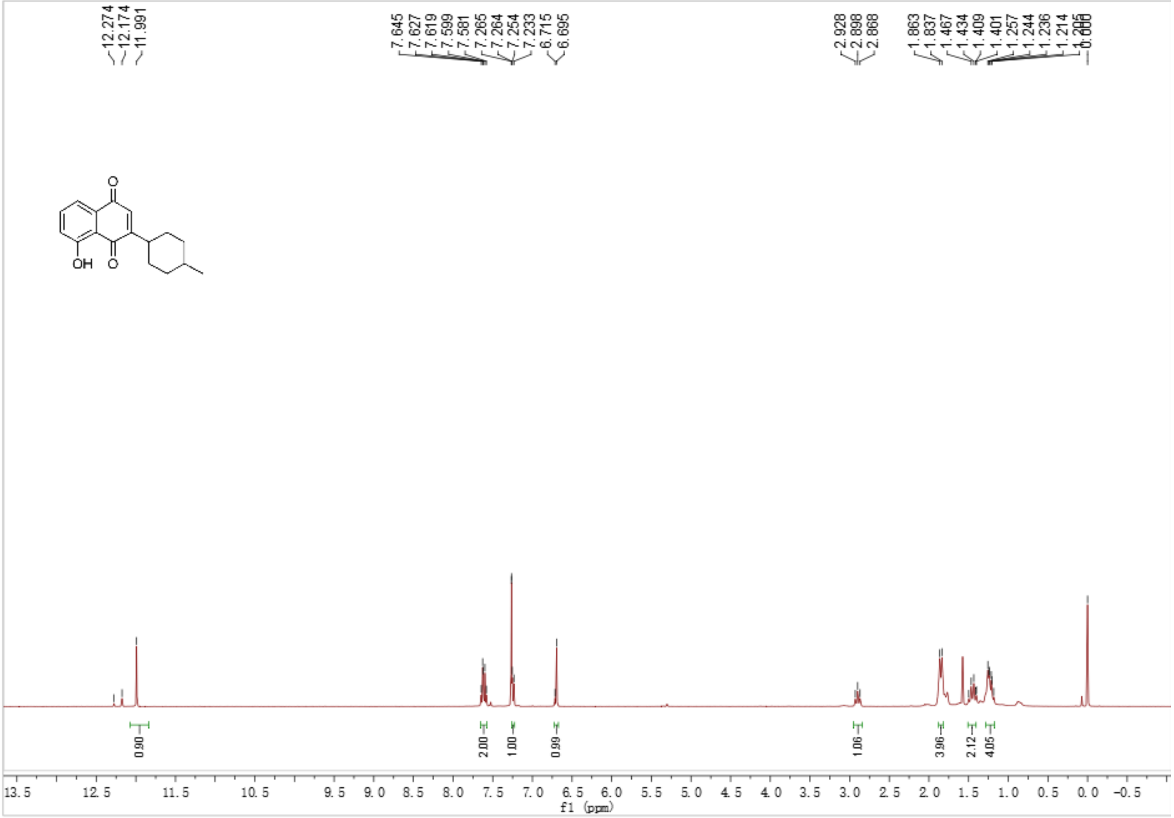

Figure S43. ^{1}H NMR spectrum of **II-1m**

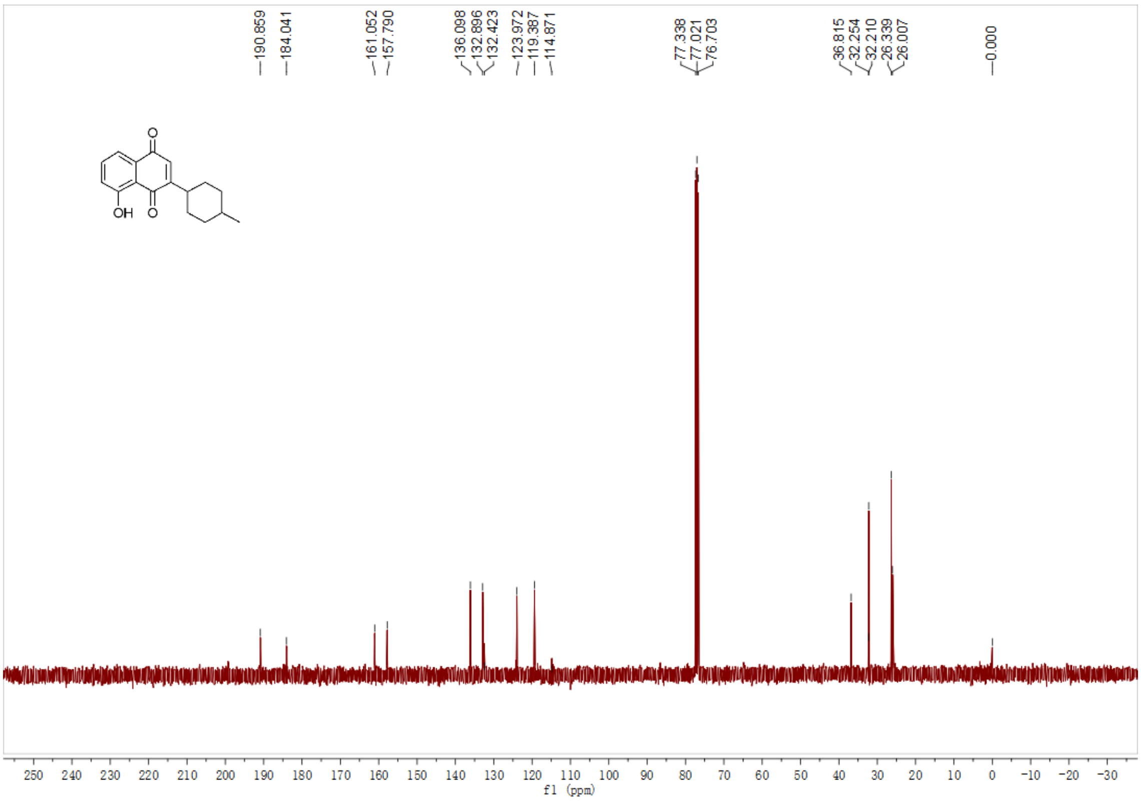

Figure S44. ^{13}C NMR spectrum of **II-1m**

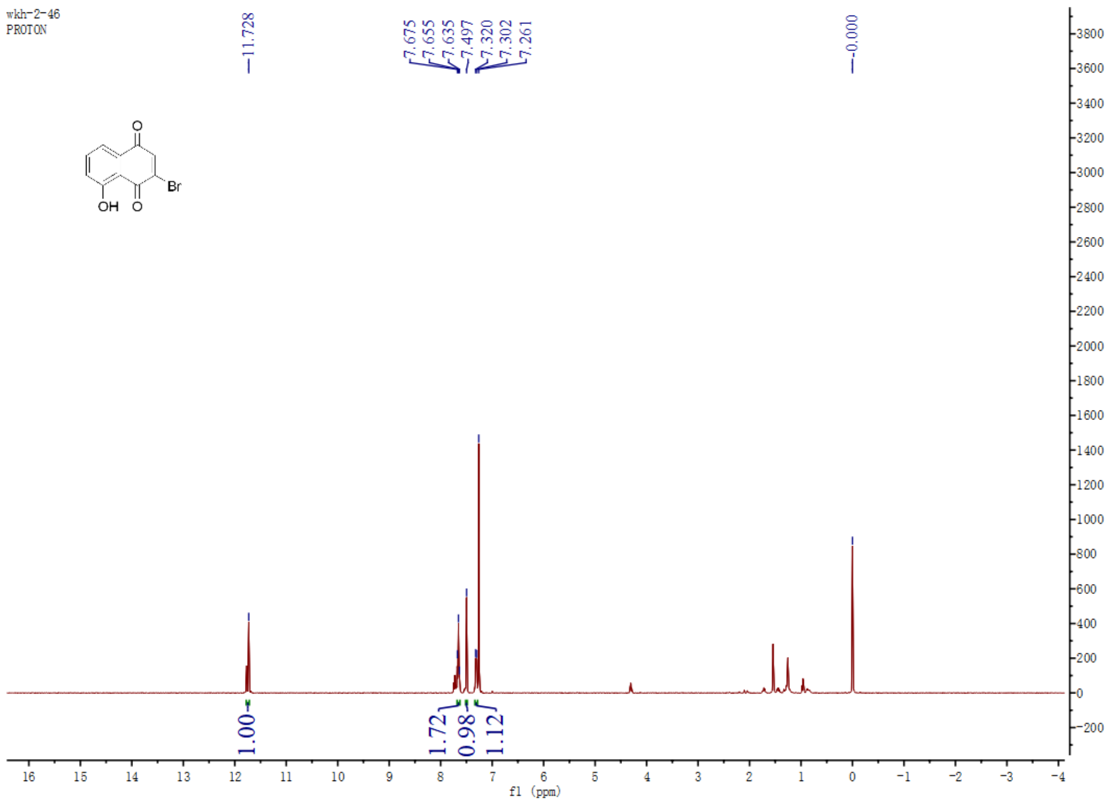

Figure S45. ^{1}H NMR spectrum of **III**

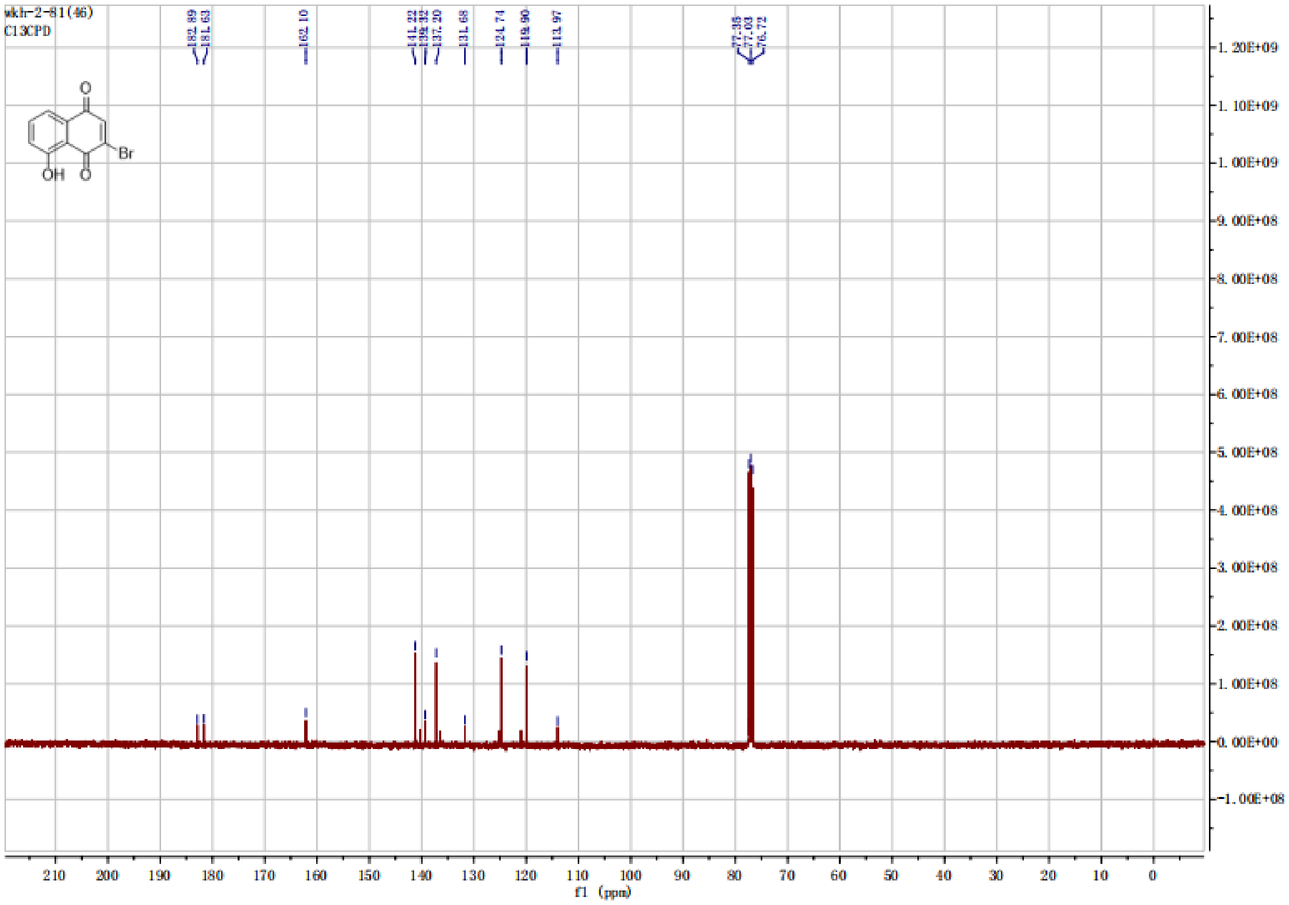

Figure S46. ^{13}C NMR spectrum of **III**

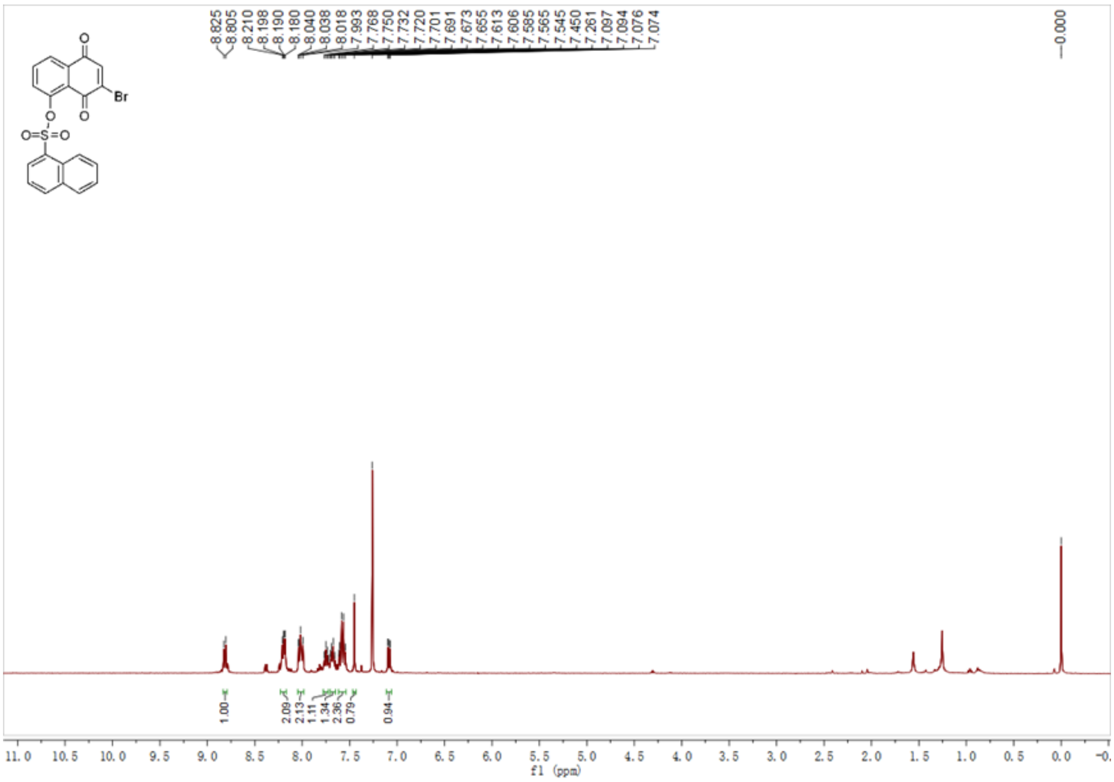

Figure S47. ^{1}H NMR spectrum of **III-1a**

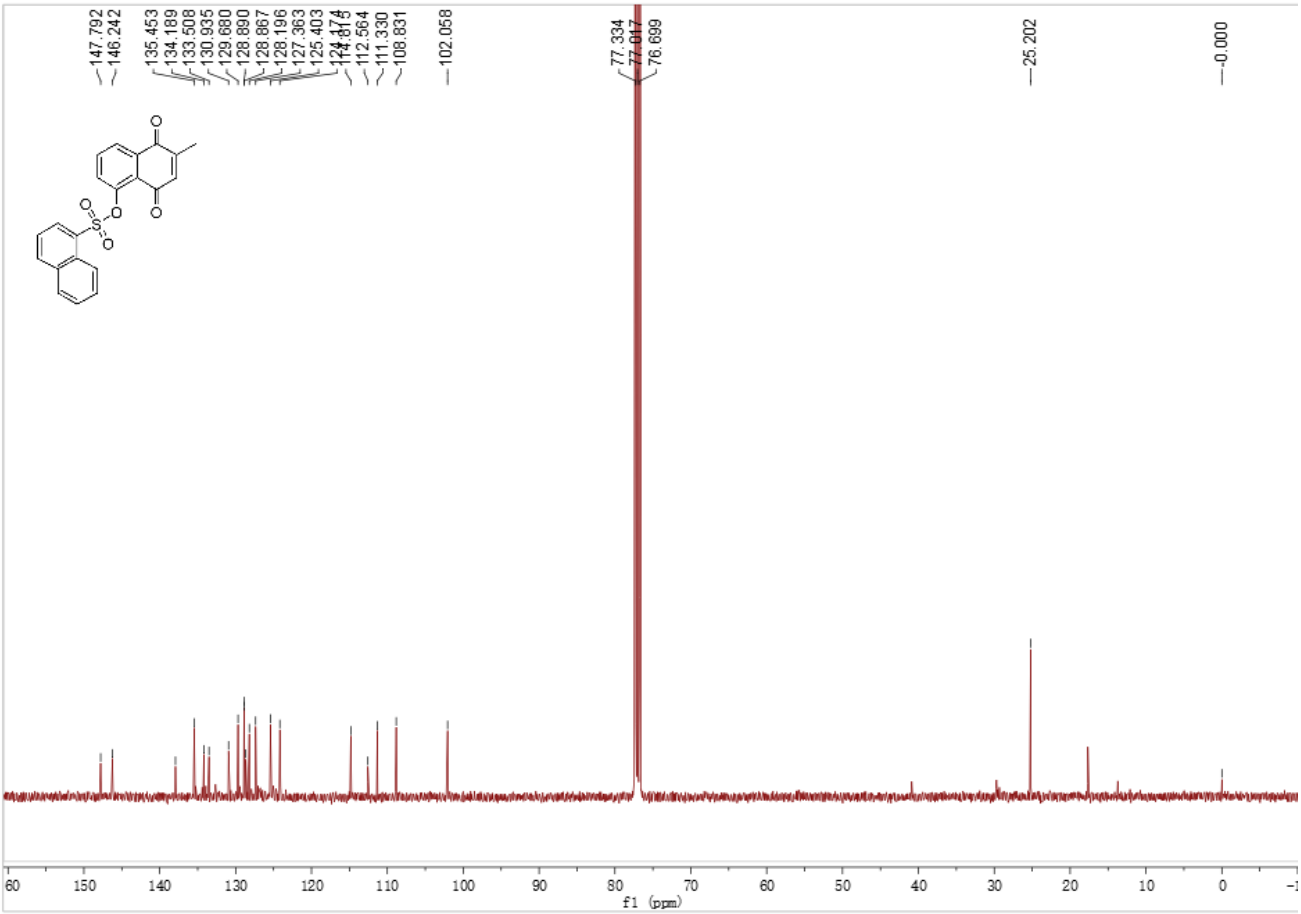

Figure S48. ^{13}C NMR spectrum of **III-1a**

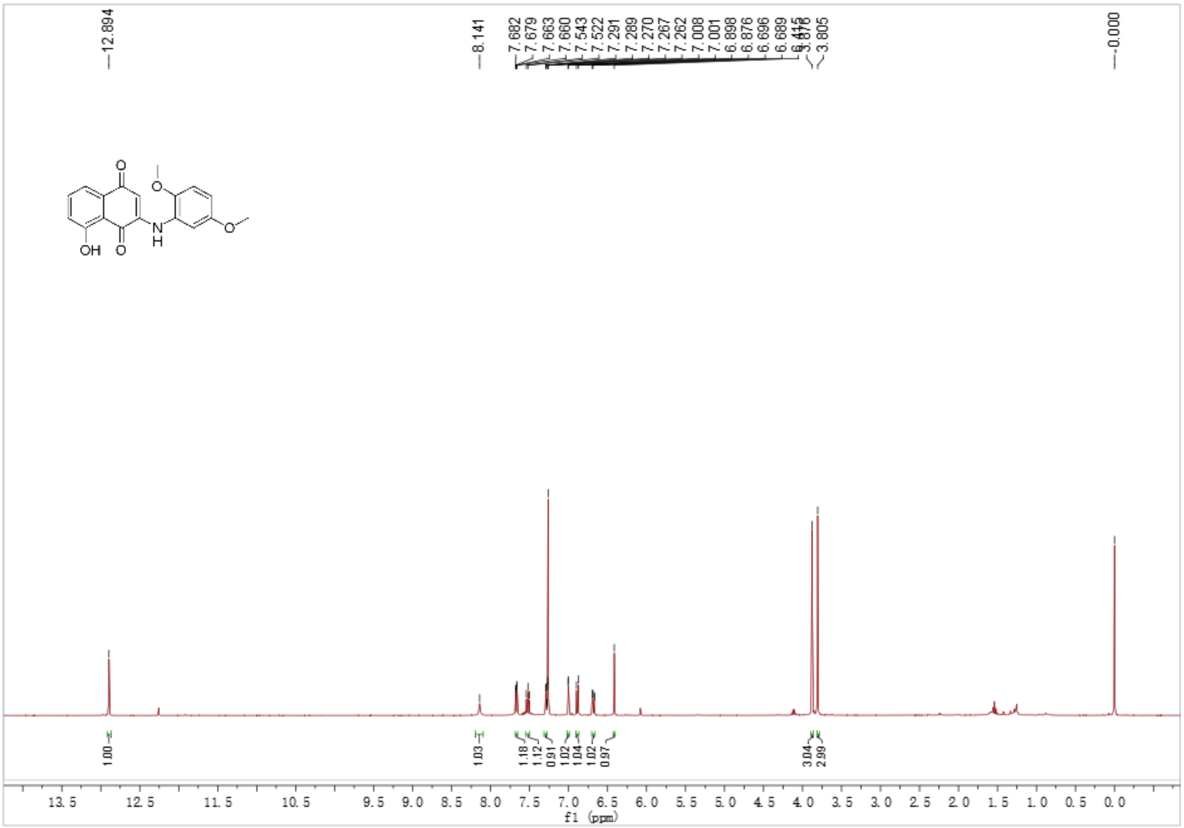

Figure S49. ^{1}H NMR spectrum of **III-1b**

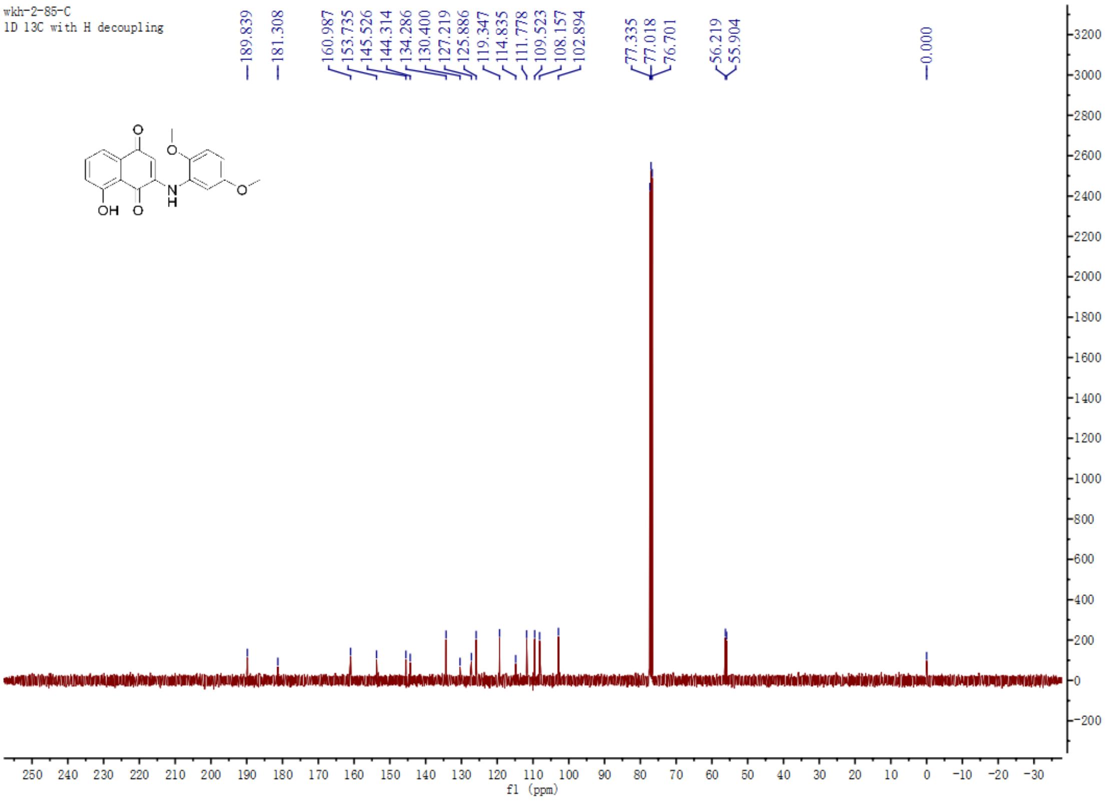

Figure S50. ^{13}C NMR spectrum of **III-1b**

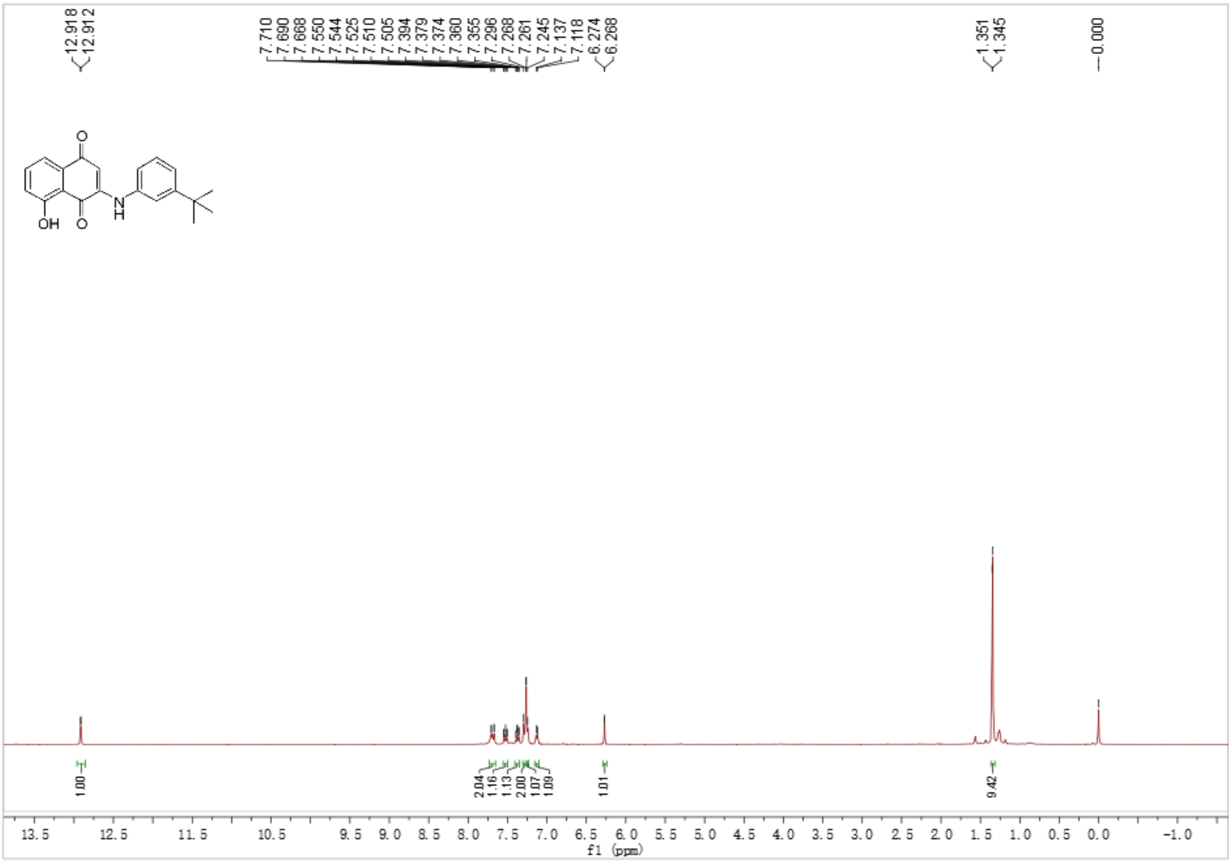

Figure S51. ^{1}H NMR spectrum of **III-1c**

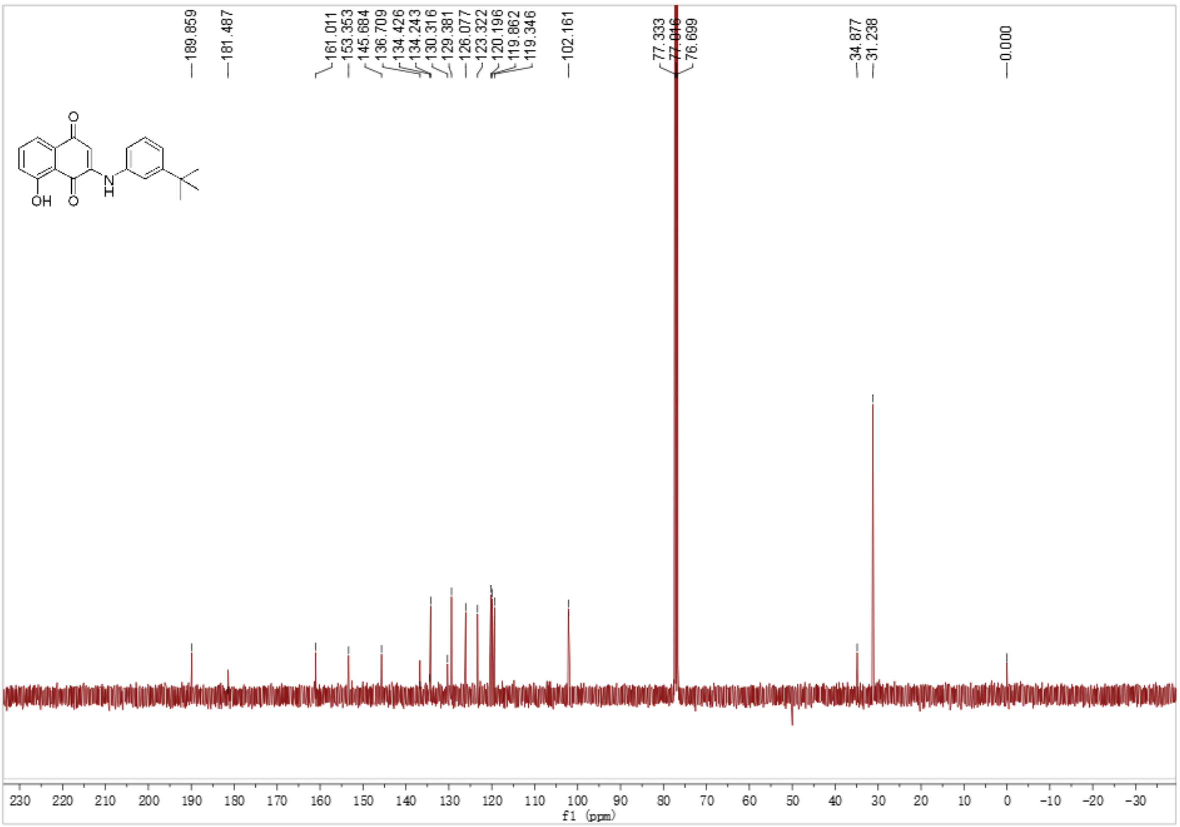

Figure S52. ^{13}C NMR spectrum of **III-1c**

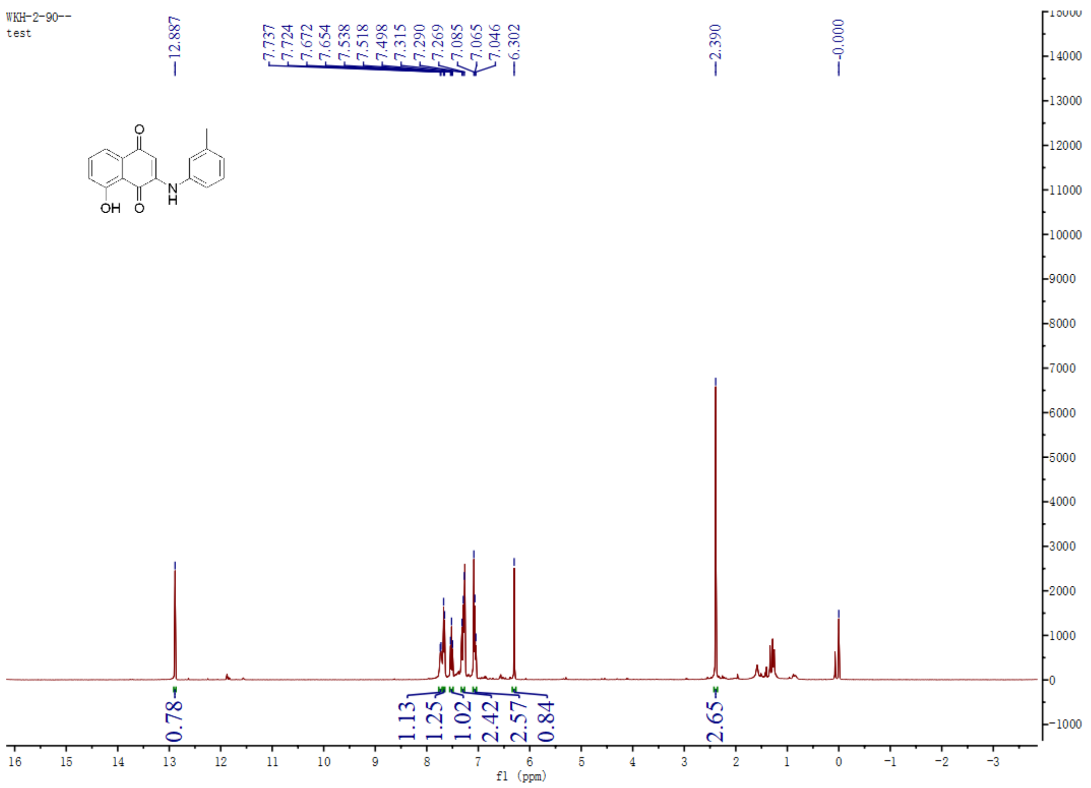

Figure S53. ^{1}H NMR spectrum of **III-1d**

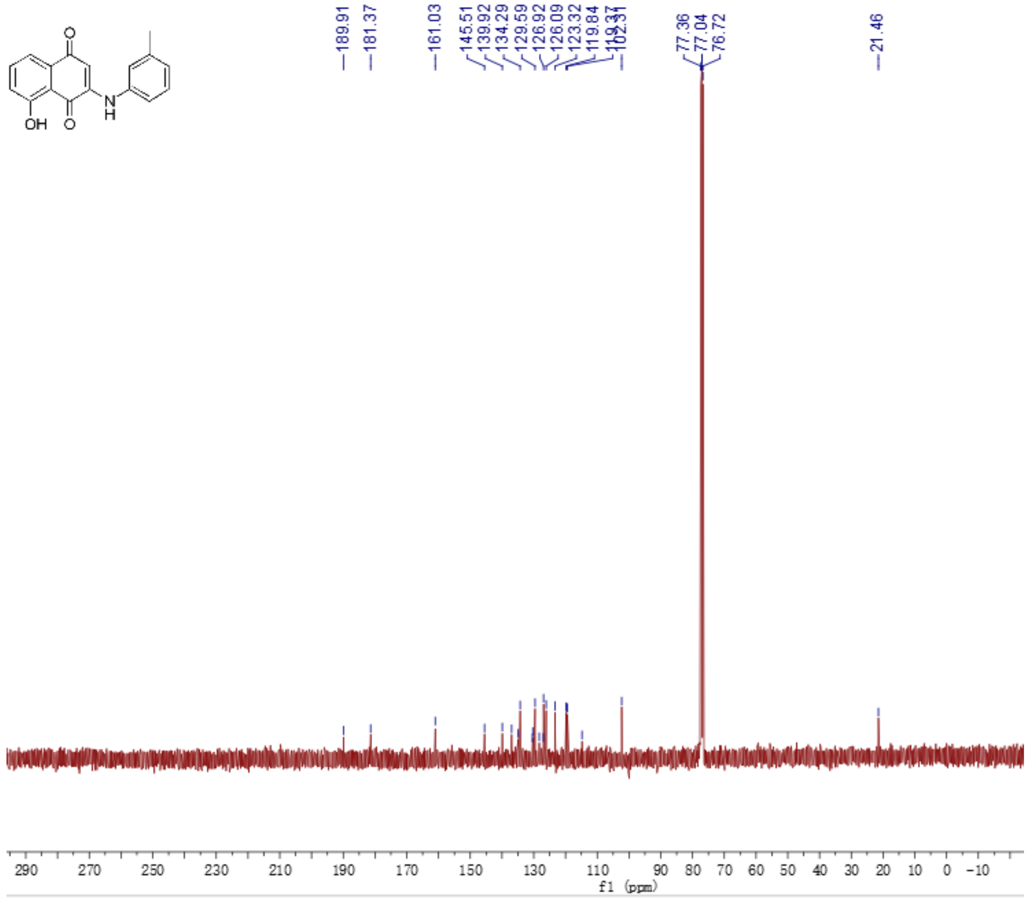

Figure S54. ^{13}C NMR spectrum of **III-1d**

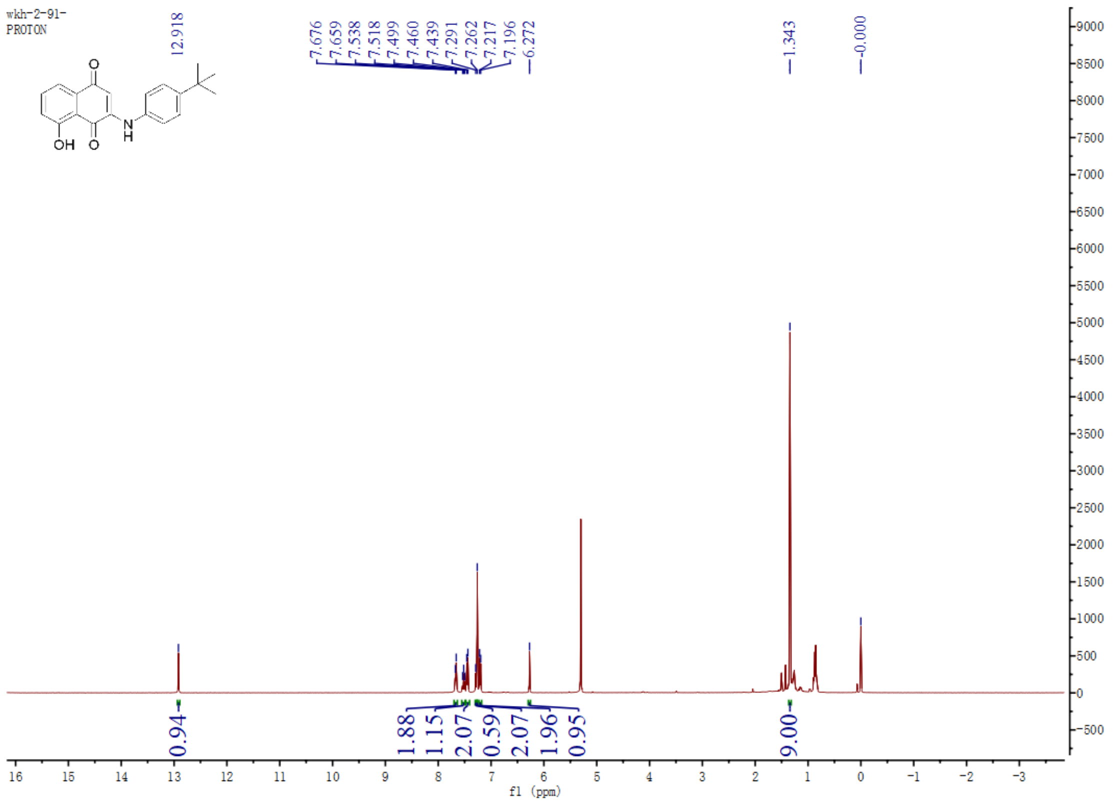

Figure S55. ^{1}H NMR spectrum of **III-1e**

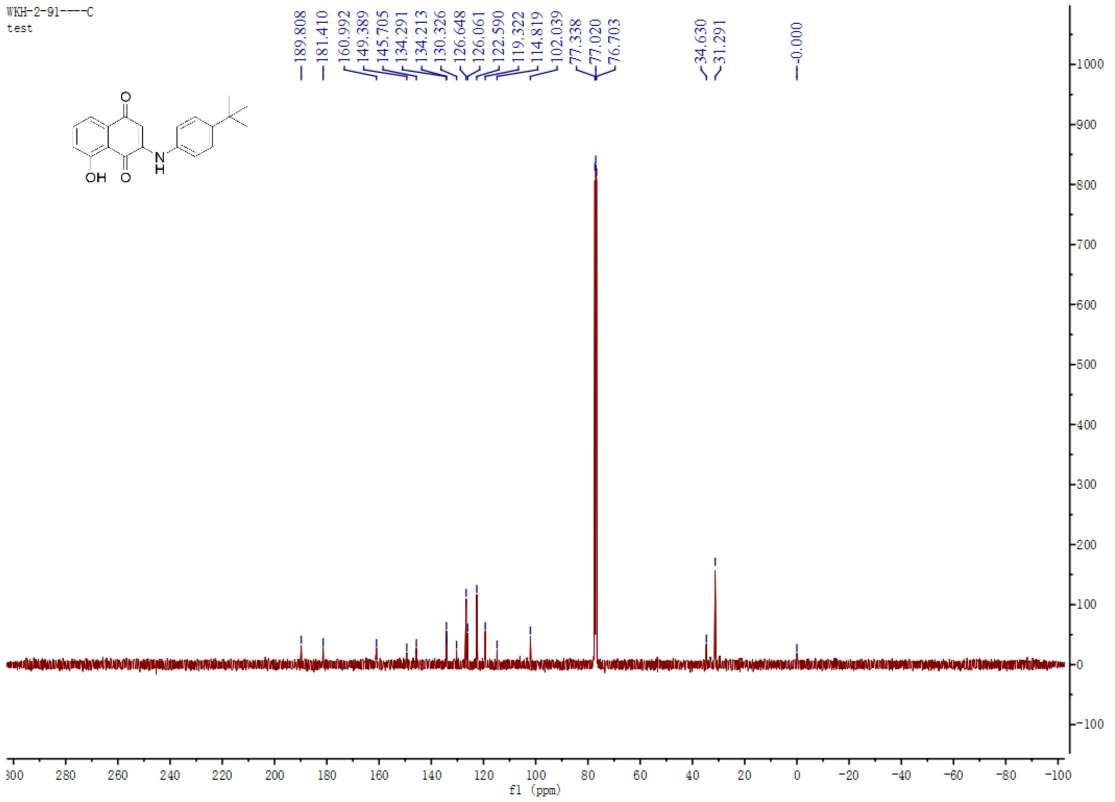

Figure S56. ^{13}C NMR spectrum of **III-1e**

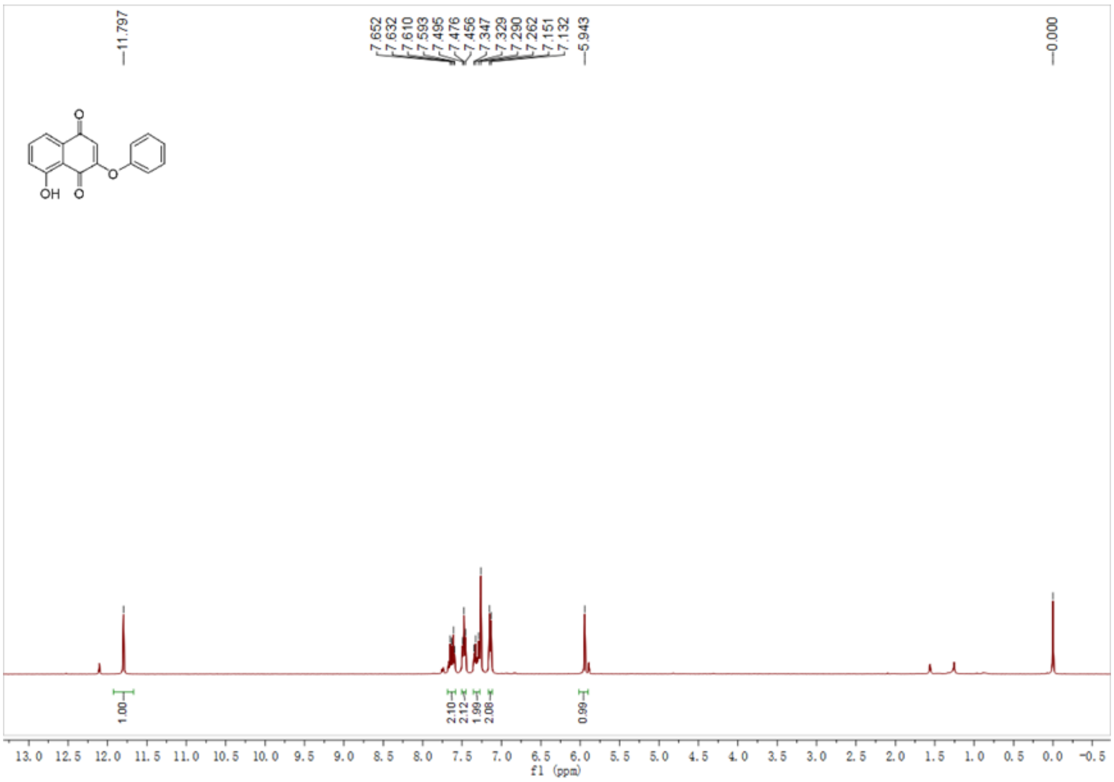

Figure S57. ^{1}H NMR spectrum of **III-1f**

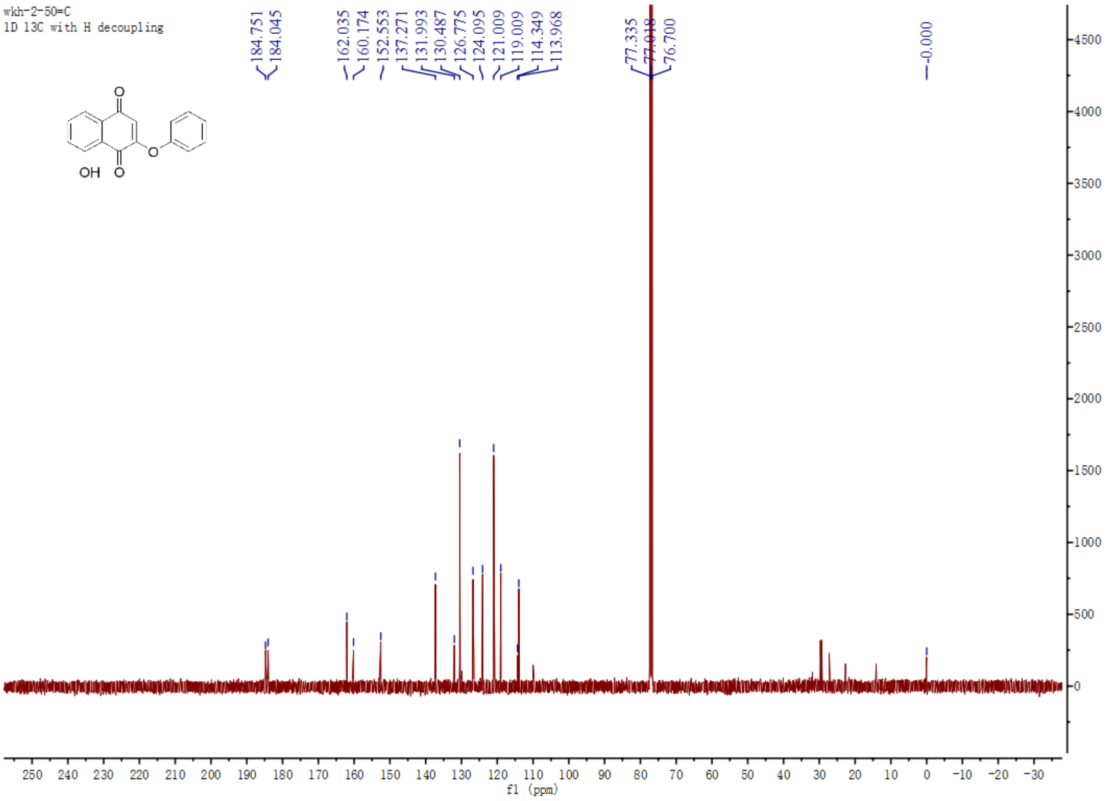

Figure S58. ^{13}C NMR spectrum of **III-1f**
